
JOURNAL INFORMATION
==============================
NLM Title Abbreviation: Insights Imaging
Journal ID: Insights Imaging
NLM Journal ID: 101532453

Insights into Imaging

EISSN: 1869-4101
Publisher: Springer

ARTICLE INFORMATION
==============================
PMCID: PMC11405745
DOI: 10.1186/s13244-024-01789-3
Article ID: 1789
Article version: 1
Subjects: Meeting Abstracts

ESHNR 2024 Book of Abstracts

Electronic publication date: 2024 Sep 12
Publication date: 2024 Sep
Volume: 15
Issue: Suppl 3
Issue sponsor: Publication costs were funded by the European Society of Head and Neck Radiology.
Electronic Location ID: 221
Copyright: © The Author(s) 2024
Copyright year: 2024
License: Open Access This article is licensed under a Creative Commons Attribution 4.0 International License, which permits use, sharing, adaptation, distribution and reproduction in any medium or format, as long as you give appropriate credit to the original author(s) and the source, provide a link to the Creative Commons licence, and indicate if changes were made. The images or other third party material in this article are included in the article's Creative Commons licence, unless indicated otherwise in a credit line to the material. If material is not included in the article's Creative Commons licence and your intended use is not permitted by statutory regulation or exceeds the permitted use, you will need to obtain permission directly from the copyright holder. To view a copy of this licence, visit http://creativecommons.org/licenses/by/4.0/.
License URL: https://creativecommons.org/licenses/by/4.0/

Conference name: ESHNR 2024
Conference location: Malmö, Sweden
Conference date: 12-14 September 2024
issue-copyright-statement: © European Society of Radiology (ESR) 2024

==============================

EUROPEAN SOCIETY OF RADIOLOGY

### Insights into Imaging

ESHNR 2024 Book of Abstracts
Volume 15 / Supplement 3

Springer eshnr european society of
head and neck radiology

### Masterclass in H&N radiology

**November 21-23, 2024**
**Manchester, UK**

**Register now!**

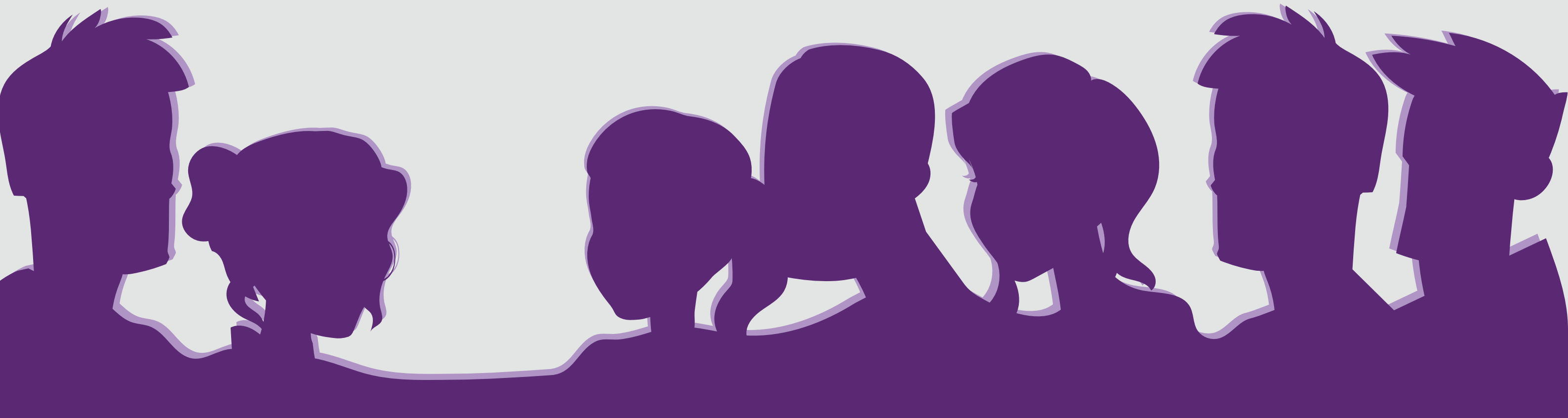

www.eshnr.eu

### Congress Programme

An exciting scientific program will cover the major topics in head and neck radiology with a special focus on modern imaging techniques, salivary glands, paediatric, lower neck and transpatial lesions, the temporal bone and sinonasal pathology. There will be a session on updates in cancer imaging and a tumour board with Swedish experts and junior radiologists from other countries. The refresher courses will cover the temporal bone, sinonasal region and head and neck infections.

The successful cooperation with our sister society ASHNR continues. Dedicated sessions with experts from both sides of the Atlantic will discuss NI-RADS, thyroid pathology and the differences in head and neck radiology education. These will be supplemented by case-presentations from junior faculty. We are very happy to welcome our American colleagues to Sweden!

We are excited about our new cooperation with the European Academy of Dentomaxillofacial Radiology. Specialists from both societies will talk about the imaging of dental and TMJ pain. A session about the crossover problems for head and neck and odontological radiologists completes the list.

### Organising Secretariat

Education Congress Research GmbH/ESHNR
Am Gestade 1
1010 Vienna, Austria
office@eshnr.eu | www.eshnr.eu

### Confirmation of Attendance

Each participant who successfully registered and attended the live event will receive a confirmation of attendance after the meeting.

### Disclaimer

The ESHNR 2024 Book of Abstracts summarises the presentations accepted to be held at the Annual Scientific Meeting – Malmö/Sweden, September 2024. Abstracts were submitted by the authors warranting that good scientific practice, copyrights and data privacy regulations have been observed and relevant conflicts of interest declared. Abstracts reflect the authors' opinions and knowledge. The ESHNR does not give any warranty about the accuracy or completeness of medical procedures, diagnostic procedures or treatments contained in the material included in this publication. The views and opinions presented in all given abstracts and presentations, including scientific, educational and professional matters, do not necessarily reflect the views and opinions of the ESHNR. In no event will the ESHNR be liable for any direct or indirect, special, incidental, consequential, punitive or exemplary damages arising from the use of these abstracts.

In preparing this publication, every effort has been made to provide the most current, accurate, and clearly expressed information possible. Nevertheless, inadvertent errors in information can occur. The ESHNR is not responsible for typographical errors, accuracy, completeness or timeliness of the information contained in this publication.

Insights Imaging (2024) Vol 15 (Suppl 3)
https://doi.org/10.1186/s13244-024-01789-3
Published: 12. September 2024

eshnr

### ESHNR Membership

**The European Society of Head and Neck Radiology, Europe's leading head and heck imaging society, welcomes anyone with a substantial interest in head and neck imaging.**

### Membership benefits

» **Reduced registration fee for**
- the ESHNR Annual Meeting and Refresher Course
- the ESHNR Masterclass
- all additional ESHNR events

» **Eligibility to apply for**
- the ESHNR Mentoring Programme
- the European Board in Head and Neck Radiology Diploma
- the ESOR-ESHNR fellowship programme

» **Free access to**
- live ESHNR webinars (incl. CME certificate)
- live ESHNR Case webinars
- live ESHNR MDT Case webinars
- live ASHNR-ESHNR joint webinars (incl. CME certificate)
- all webinar recordings

**plus** » Membership certificate » ESHNR newsletter » and much more!

**Apply now on www.eshnr.eu!**

eshnr

### ESHNR Mentoring Programme new support initiative

*

one year mentoring programme

**ESHNR calls for mentors and mentees and will match and introduce them**

**Programme coordinators**
Steve Connor, London/UK
Soraya Robinson, Vienna/AT

www.eshnr.eu

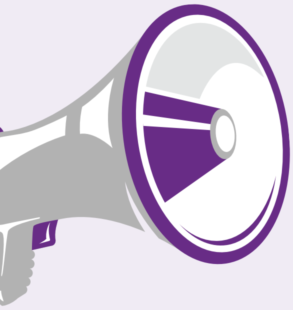

**Become a mentee or mentor**

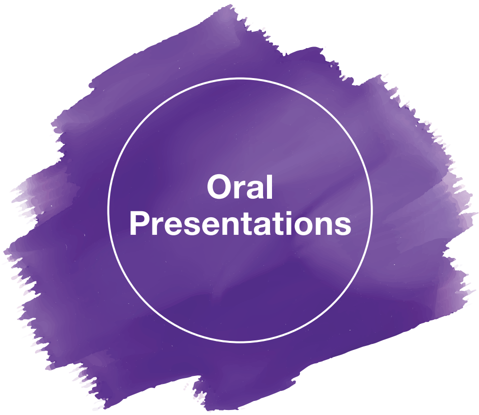

### Redefining Sinonasal Cancer response assessment to induction chemotherapy with tumor volumetry, results from two phase II non randomised controlled prospective multicentric trials: SINTART-1 and SINTART-2

*Authors: P. Rondi[1], C. Resteghini[2], A. Borghesi[1], P. Bossi[2], M. Ravanelli[1], D. Farina[1]; [1]Brescia/IT, [2]Rozzano (Milano)/IT*

**Purpose/Objectives:** Aim of this study is to identify the best response criteria in patients with sinonasal cancer undergoing induction chemotherapy (IC).
**Methods and materials:** This prospective multicenter study was approved by internal review board (IRB) of each of the involved centers. Informed consent was available for all the patients. The study was conducted in accordance with the guidelines for Good Clinical Practice and Declaration of Helsinki.
Patients enrolled in SINTART-1[1] and SINTART-2[2] (ClinicalTrials.gov Identifier: NCT02099175 and NCT02099188, respectively) were included in this study. Unidimensional diameters (antero-posterior, AP; latero-lateral, LL; cranio-caudal, CC), maximum axial area (Amax) and volume (V) were performed on MRI by two radiologists. RECIST 1.1[3] assessment was included as a categorical variable. Variables were evaluated at baseline, after 1st chemotherapy cycle and at best response. Interobserver repeatability was analyzed.
Stepwise univariable and multivariable Cox proportional-hazards regression models were used to correlate variables with Disease-Free Survival (DFS) and Overall Survival (OS).
**Result:** 60 patients were included in this study. Interobserver correlation at baseline and after 1st IC cycle was excellent for V (0.916 and 0.928 respectively), CC diameter (0.96 and 0,863) and AP diameter (0.846 and 0.796); good for LL diameter and moderate for Amax.
Results for univariable and multivariable analysis are reported in Table 1 and Table 2.

| | OS (after 1 IC cycle) | DFS (after 1 IC cycle) | OS (best response) | DFS (best response) |
|---|---|---|---|---|
| ΔAP | n.s. | n.s. | n.s. | n.s. |
| ΔLL | HR 0.06, p=0.004 | HR 0.045, p=0.002 | HR 16.3, p=0.01 | n.s. |
| ΔCC | HR 0.15, p=0.04 | HR 0.12, p=0.014 | n.s. | n.s. |
| ΔV | HR 0.1, p<0.0001 | HR 0.065, p<0.0001 | HR 0.06, p=0.002 | HR 0.1, p=0.005 |
| ΔAP > 30% | n.s. | n.s. | n.s. | n.s. |
| ΔLL > 30% | n.s. | n.s. | n.s. | n.s. |
| ΔCC > 30% | HR 0.32, p=0.031 | HR 0.35, p=0.019 | n.s. | n.s. |
| ΔV > 50% | HR 0.36, p=0.021 | HR 0.33, p=0.012 | HR 0.32, p=0.014 | HR 0.39, p=0.03 |
| RECIST 1.1 | n.s. | n.s. | n.s. | n.s. |

Table 1: Stepwise univariable Cox proportional-hazards regression models analysis results for each variable considered in the study. Hazard ratio (HR), statistically not significant (n.s.)

| | OS (after 1 IC cycle) | DFS (after 1 IC cycle) | OS (best response) | DFS (best response) |
|---|---|---|---|---|
| ΔLL | n.s. | n.s. | n.s. | n.s. |
| ΔCC | n.s. | n.s. | n.s. | n.s. |
| ΔV | HR 0.12, p<0.001 | HR 0.08, p=0.0004 | HR 0.07, p=0.0019 | HR 0.12, p=0.004 |
| ΔCC > 30% | n.s. | n.s. | n.s. | n.s. |
| ΔV > 50% | HR 0.32, p=0.029 | HR 0.37, p=0.032 | HR 0.32, p=0.014 | HR 0.45, p=0.051 |

Table 2: Stepwise multivariable Cox proportional-hazards regression model analysis results. HR: hazard ratio, statistically not significant (n.s.).

RECIST 1.1 criteria after 1st IC cycle and at best response were not correlated with OS and DFS. Volume variation after 1st IC cycle and at best response was the variable most correlated with OS (p<0.0001and p=0.002) and DFS (p<0.0001and p=0.005).
At the multivariable analysis V variation after 1st IC cycle and at best response resulted to be the only variable statistically associated with OS(p<0.001 and p=0.0019 respectively) and DFS(p=0.0004 and p=0.004 respectively).
Figure 1 and Figure 2 show survival curves in patients according to ΔV (OS and DFS after 1 IC cycle and at the best response).

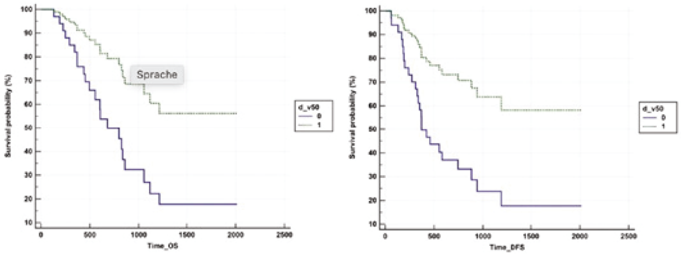

Figure 1: Overall Survival and Disease-Free Survival curves in patients with a volume reduction more (1) or less (0) than 50% (d_v50) after the first IC cycle

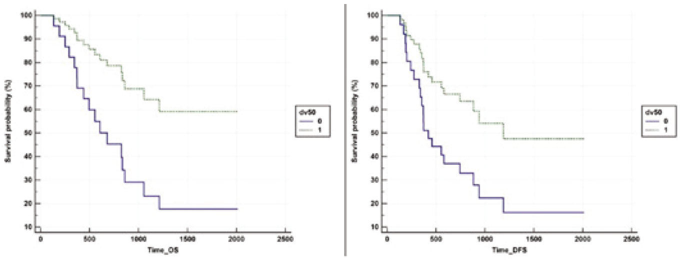

Figure 2: Overall Survival and Disease-Free Survival curves in patients with a volume reduction more (1) or less (0) than 50% (d_v50) at best response.

**Conclusion:** Volume variation should be preferred to RECIST 1.1 and other measurements as objective radiological response in sinonasal cancer.
This study suggests tumor volumetry should be considered the gold standard for assessing response to IC in sinonasal cancer. Volume is the only parameter associated with survival thus being useful for prognostic stratification and in the decision-making process. The cut-point of 50% is excellent to discriminate partial response from stable disease and could be easily used in clinical practice.

References:
[1] Resteghini, Carlo et al., (2023), The SINTART 1 study. A phase II non-randomised controlled trial of induction chemotherapy, surgery, photon-, proton- and carbon ion-based radiotherapy integration in patients with locally advanced resectable sinonasal tumours, European journal of cancer, Oxford, England
[2] Bossi, Paolo et al, (2023), The SINTART 2 Study. A phase II non-randomised controlled trial of induction chemotherapy, photon-, proton- and carbon-ion-based radiotherapy integration in patients with locally advanced unresectable sinonasal tumours, European journal of cancer, Oxford, England
[3] Eisenhauer EA, Therasse P, Bogaerts J, et al, (2009), New response evaluation criteria in solid tumours: revised RECIST guideline (version 1.1), European journal of cancer, Oxford, England

### Predicting response to exclusive combined radio-chemotherapy in naso-oropharyngeal cancer: The role of texture analysis

*Authors: E. Bicci[1], L. Calamandrei[1], M. Pietragalla[2], S. Busoni[1], S. Paolucci[1], F. Mungai[1], C. Nardi[1], L. Bonasera[1], V. Miele[1]; [1]Florence/IT, [2]Pistoia/IT*

**Purpose/Objectives:** Identifying MRI texture features able to predict response to radiochemotherapy in patients with naso-oropharyngeal carcinoma before treatment to help clinical decision making.
**Methods and materials:** Texture analysis was performed on ADC-maps and post-gadolinium T1-images on a single MRI machine of 40 patients with naso-oropharyngeal carcinoma (NPC-OPC). Patients were divided into 2 groups (responders/non-responders) according to MRI, 18F-FDG-PET/CT performed 3-4 and 12 months after RT-CHT, and biopsy. Pre-RT-CHT lesions were segmented and radiomic features were extracted. Non-parametric Mann–Whitney test was performed. A p-value<0.05 was considered significant. Receiver Operating Characteristic curves and Area Under the Curve (AUC) values were generated; a 95% confidence interval (CI) was reported. A radiomic model was constructed using the LASSO algorithm.
**Result:** After features selection on MRI T1 post-contrast sequences six features were statistically significant gldm_DependenceEntropy and DependenceNonUniformity, glrlm_RunEntropy and RunLengthNonUniformity, glszm_SizeZoneNonUniformity and ZoneEntropy with significant cut-off values between responders and non-responders group. In particular, all these features showed higer values on pre-RT-CHT MRI images in patients with tumor persistence/recurrence, in accordance with the greater structural heterogeneity in lesions unresponsive to therapy. With LASSO algorithm the radiomic model showed an AUC: 0.89 and 95%CI: 0.78-0.99. In ADC five features were selected with AUC: 0.84 and 95%CI: 0,68-1.
**Conclusion:** Texture analysis on post-gadolinium T1-images and ADC-maps could potentially predict response to therapy in patients with NPC-OPC who will undergo exclusive treatment with RT-CHT, being, therefore, a useful tool in therapeutical-clinical decision-making.

### Diagnostic Accuracy of Imaging Findings [CT, MRI and USG] in the detection of lymph node metastasis in patients with Head And Neck Cancer: A retrospective analysis

*Authors: S.N. Singh, S. Shukla, V. Gadupudi; Varanasi/IN*

**Purpose/Objectives:** The objective of this retrospective study was to evaluate the precision of radiological imaging methods, such as CT, MRI, and USG, in identifying lymph node metastasis among individuals diagnosed with head and neck cancer.
**Methods and materials:** A total of 1412 patients with head and neck cancers who underwent pre-operative imaging at our tertiary cancer institute between January 2020- 2023 were included. Two experienced radiologists blinded to the histopathology results independently reviewed the imaging findings. They classified the nodes at each level as benign, indeterminate, suspicious for metastasis, and metastatic. The number and level of metastatic lymph nodes and the presence or absence of extranodal extension were compared to the histopathological findings. The primary histopathological subtype and degree of differentiation were also recorded. The information gathered was analyzed using IBM SPSS Statistics. Frequency analysis and percentage analysis were used to describe the data. For each modality and level, the sensitivity, specificity, positive predictive value (PPV), negative predictive value (NPV), and accuracy were calculated.
**Result:** CT, MRI, and USG had high diagnostic accuracy: CT (82.4%), MRI (81.1%), and USG (82.4%). All modalities showed high sensitivity (CT: 92.8%, MRI: 89.4%, USG: 89.5%) and negative predictive value (CT: 84.8%, MRI: 85.5%, USG: 88.8%). Sensitivity, specificity, PPV, and NPV varied by level, with the highest values in level V (MRI: 99.4% sensitivity, 99% specificity). The highest specificity and PPV were found for level II (USG had a specificity of 46.1% and PPV of 54%). In indeterminate nodes, 85% were negative for metastases and 15% were positive. Additional imaging or sampling may help avoid overdiagnosis in such cases. For assessing extranodal extension (ENE), combined imaging sensitivity -38.5 %, specificity -92.7 %, PPV -16.8 %, and NPV-97.5%.
**Conclusion:** Accurate pre-operative staging is crucial due to ENE's impact on disease upstaging and treatment considerations. Imaging showed high accuracy, sensitivity, and NPV in detecting lymph node metastases and extranodal extension in head and neck malignancies, indicating their value in predicting the absence of lymph node metastasis and reducing unnecessary neck dissections. Imaging specificity and PPV were relatively lower, urging re-examining criteria for metastatic nodes. The content, quality, and effective communication of imaging findings to treating physicians are essential for optimal patient care.

### MR in diagnosis of metastatic Lymph Node in patient with Oral Squamous Cell Carcinoma

*Authors: P. Buscemi[1], A. Monteleone[1], Q.Y.H. Ai[2], R. Cannella[1], F. Bencivinni[1], A. Lo Casto[1]; [1]Palermo/IT, [2]Hong Kong/HK*

**Purpose/Objectives:** The occurrence of metastatic lymph nodes (LN) in lateral cervical stations has a significant impact on lifespan of patients with oral squamous cell carcinoma (OSCC), influencing treatment and in particular the surgical approach to a possible lymphadenectomy. MRI is often used to detect the presence of metastatic LN in patients with OSSC, using consolidated criteria, and amon them the short axial diameter (SAD). The aim of this study was to evaluate the diagnostic performance of MRI in diagnosis of metastatic LN in patients with OSCC, also comparing different SAD tresholds.

**Methods and materials:** A retrospective analysis of head and neck MRI exams, operative records, and postoperative histological reports of 83 consecutive OSCC patients, studied between 2017 and 2023, was performed. Patients were enrolled if they underwent surgery with LN dissection, if histological reports were available in the medical records, and if it was possible to retrieve the radiological reports and MR images on institutional PACS. The evaluation of malignancy in LN was based on consolidated morphological criteria. The threshold for evaluating the short axial diameter (SAD) was set at 10 mm, except for the jugulodigastric node where it was set at 11 mm. The presence of extra-nodal diffusion and colliquation were also considered in the evaluation. MRI (1.5T and 3T) studies included T1 sequences with and without fat saturation and i.v. contrast medium administration, T2 and STIR sequences on the axial plane with a maximum slice thickness of 4 mm. Recent research suggests that the SAD threshold could be reduced. Therefore, supplementary analysis was conducted using a SAD treshold of 6 mm and 7 mm.

**Result:** 21 (25.3%) true positives, 33 (39.7%) true negatives, 16 (19.2%) false positives, and 13 (15.7%) false negatives were obtained by MRI applying consolidated criteria. Colliquation occurred in 29 out of 83 patients (median SAD: 14 mm), but it was only a useful finding in 2/83 patients for classifying an LN as malignant with SAD less than 10 mm. Lowering the SAD threshold to 7 mm resulted in seven false negatives (8.4%) with a sensitivity of 79.4%. When the threshold was further lowered to 6 mm, the number of false negatives decreased to three (3.6%) with a higher sensitivity of 91.2%.

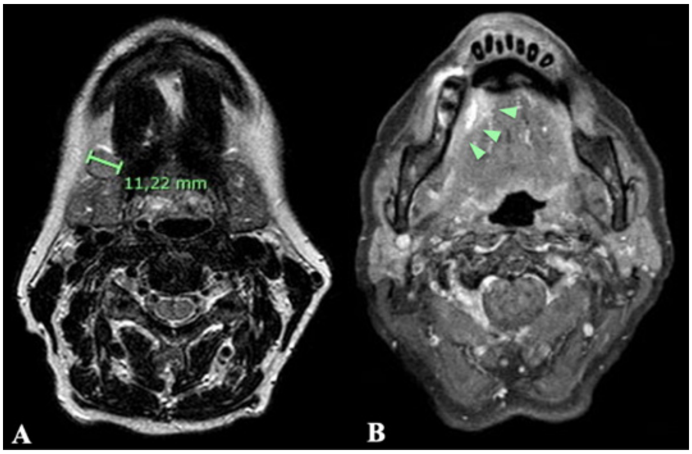

A 63 y.o. patient affected by OSCC of the tongue with false positive lymph node at MRI. A) TSE T2 axial image. The enlarged lymph node (SAD = 11 mm) at IB level on the right was evaluated as metastatic at MRI but proved normal at histology. B) TSE T1 fat sat after i.v. Gd axial image. Enhancing tumor in the right tongue (arrowheads).

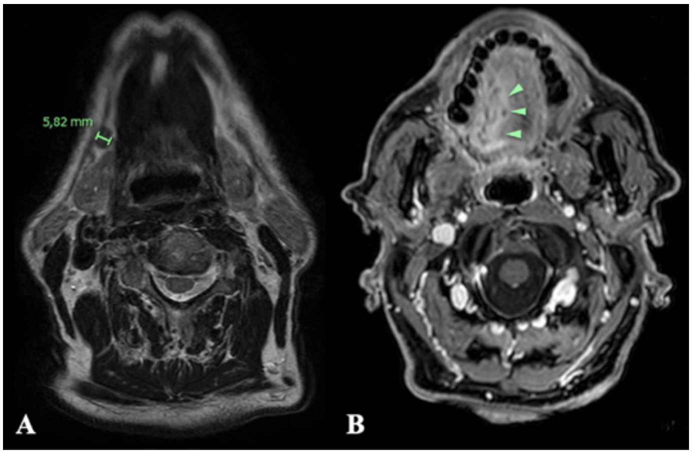

A 72 y.o. patient affected by OSCC of the tongue with false negative lymph node at MRI. A) TSE T2 axial image. The lymph node (SAD= 6 mm) at IB level on the right was considered normal at MRI but proved metastatic at histology. FFE T1 fat sat after i.v. Gd axial image. Enhancing tumor in the right tongue (arrowheads).

| **Table 1**. Demographic, Histological, and Imaging Data | **Results (n=83)** |
|---|---|
| **SAD evaluation N+ (≥10 mm, gd ≥11 mm), n (%)** | |
| *Positive* | 37 (44.6) |
| *Negative* | 46 (55.4) |
| **Colliquation, n (%)** | |
| *SAD ≥ 10 mm* | 27 (32.5) |
| *SAD ≤ 10 mm* | 2 (2.4) |
| **Post-Histological Analysis, n (%)** | |
| *FP;TP;FN;TN* | 16 (19.2); 21 (25.3); 13 (15.7); 33 (39.7) |
| *FP+FN* | 29 (34.9) |
| *Se; Sp* | **61.8%; 67.4%** |
| **SAD ≥ 7 mm, n (%)** | |
| *FN* | 7 (8.4) |
| *Se* | 79.4%; |
| **SAD ≥ 6 mm, n (%)** | |
| *FN* | 3 (3.6) |
| *Se* | 91.2%; |

Principal results

**Conclusion:** The currently used criteria for considering LN as metastatic on MRI lead to a high number of false positives and false negatives. Lowering the SAD threshold appears to be a promising strategy for improving the diagnostic performance of MRI in the detection of metastatic LN, increasing sensitivity, with an expected decrease in specificity.

### Exploring the Application Value of Dual-Energy Enhanced CT for Preoperative Prediction of Cervical Lymph Node Metastasis without Detected Central Necrosis in Laryngeal Squamous Cell Carcinoma

*Authors: Y. Zhang, Y. Sha, R. Hong, W. Chen, H. Xiao; Shanghai/CN*

**Purpose/Objectives:** The ability of traditional Single-Energy CT (SECT) to predict lymph node metastasis is limited, especially when lymph node metastasis presents atypically. This study aims to explore the potential value of Dual-Energy Computed Tomography (DECT) in predicting preoperative lymph node metastasis of laryngeal cancer when central necrosis is not detected in the lymph nodes, and to evaluate whether it can improve diagnostic performance compared to SECT.
**Methods and materials:** This research included 115 lymph nodes (LNM-negative: 91; LNM-positive: 24) from 68 patients diagnosed with laryngeal cancer, all subjected to DECT enhanced examinations, and none detected central necrosis. Lymph node imaging characteristics were analyzed on standard mixed images of DECT, and multiple DECT quantitative parameters were extracted on virtual monoenergetic imaging at different energy levels, iodine maps, and electron density maps. Differences between basic imaging characteristics and DECT parameters were compared, and DECT parameters were subjected to univariate analysis, collinearity analysis, and point-biserial correlation analysis. Basic imaging models were constructed using significant variables, and improved models were developed by adding DECT parameters, with nested comparisons evaluating performance differences.
**Result:** Normalized Iodine Concentration (NIC) showed the best performance among all evaluated DECT parameters. Lymph node CT mean values obtained from different energy levels showed the best performance at 40 keV. The improved model incorporating the NIC parameter significantly boosted predictive performance, especially when the basic model only included the short-axis diameter and level. The improved model, combining clinical-imaging characteristics, and DECT parameters, reached a sensitivity of 0.833 and a specificity of 0.967.
**Conclusion:** DECT technology not only provides imaging features available with SECT but also offers a series of quantitative parameters, which contribute to the accuracy of predicting laryngeal cancer lymph node metastasis.

### Assessing accuracy and reliability of open-access large language models (ChatGPT, Copilot, Claude, Perplexity, and Gemini) in assigning AJCC TNM stage to Radiology reports in Head and Neck squamous cell carcinomas

*Authors: M.S. Swarup, G. Sindhwani, K. Gehlot, M. Jain, R. Kanaujia, R. Gupta Ghasi; New Delhi/IN*

**Purpose/Objectives:** To evaluate the accuracy and reliability of 5 popular open-access large language models (LLMs) namely ChatGPT3.5, Copilot, Claude, Perplexity, and Gemini in assigning radiological stages according to the latest AJCC TNM staging in various Head and Neck squamous cell carcinomas (SCCs) by interpreting radiology reports.
This will be a unique application of artificial intelligence (AI) in radiologic clinical decision support (CDS) i.e. assigning radiologic disease stages in head and neck carcinomas so that appropriate management decisions can be taken efficiently. It is practically almost impossible to accurately memorise the AJCC TNM staging descriptions of all the head and neck SCC and online web searches may be time-consuming in busy workflow departments.
**Methods and materials:** A total of 20 radiology reports of cross-sectional imaging studies (both contrast-enhanced CT and MRI) done for the initial staging of squamous cell carcinoma of various subsites in the head and neck region (buccal mucosa, gingival mucosa, hard palate, oral and base of tongue, oropharynx, nasopharynx, hypopharynx and larynx) were included in the study. Five radiologists used their login accounts for the five popular open-access LLMs on their personal computing systems to access the studied LLMs. The LLMs evaluated in our study are Open AI ChatGPT3.5, Microsoft Copilot, Anthropic Claude, Perplexity, and Google Gemini. Only the freely available versions of these AI-based chatbots are included for evaluation in our study, as these will be accessible to all, particularly in developing nations. Standard prompts were formulated for each of the 20 reports, incorporating the study findings and impressions. The same prompts were used by all five users for each report to obtain responses in different LLMs. The prompt is structured as follows *“Can you assign the TNM stage to the following head and neck cancer CT/ MRI report according to the latest AJCC staging...”*. The responses obtained from the LLMs were evaluated by respective radiologists to determine whether it is concordant or discordant with the actual radiological disease stage in each case. Reliability and consistency between different users for each LLM were also evaluated.
**Result:** A total of 100 responses were obtained for each LLM (20 reports x 5 radiologists) and the data was analysed. The most accurate and reliable AI-based LLM is ChatGPT3.5 with an overall 86 % accuracy followed by Claude, Copilot, Perplexity and Gemini in that order.
**Conclusion:** In the ever-progressive era of AI-based applications in the field of CDS, our study provides the groundwork for the use of LLMs in radiologic staging in head and neck SCCs to enhance clinical workflow with a scope of further improvement and refinement for this capability.

### Diagnostic accuracy of MRI for intracranial invasion of sinonasal malignancies: A systematic review

*Authors:* U. Abdullaeva[1], J. Hirvonen[2]; [1]Tashkent/UZ, [2]Tampere/FI

**Purpose/Objectives:** Pretreatment evaluation of the intracranial extension of sinonasal cancer is crucial for staging, prognosis, and treatment planning. Magnetic resonance imaging (MRI) is commonly considered a superior imaging tool for assessing intracranial involvement of sinonasal malignancies. However, the diagnostic accuracy of MRI in detecting intracranial invasion varies among published reports, mainly due to varying MRI features for assessing invasion (such as bone erosion, thin vs. thick dural enhancement, smooth vs. nodular dural enhancement, etc). In addition, selection, sampling, and verification bias as potential sources of heterogeneity may influence the false negative and false positive results and diagnostic accuracy of the studies. Therefore, we aimed to review the diagnostic accuracy of MRI in detecting intracranial invasion of sinonasal malignancies using histopathological or surgical evidence as the reference standard.
**Methods and materials:** Previous studies in English published in the last 33 years were searched in PubMed and Embase databases. The study was registered in Prospero (CRD42024492090). We included studies that: 1) used preoperative MRI, with and without contrast enhancement, to detect intracranial invasion of sinonasal malignancies; 2) presented histological or surgical confirmation of intracranial invasion; and 3) reported patient numbers in each class in sufficient detail to calculate diagnostic accuracy (sensitivity, specificity, positive and negative predictive value (PPV, NPV), and accuracy).
**Result:** We found 6 studies reporting 488 patients with sinonasal tumors in whom intracranial invasion was assessed with MRI and surgery/histopathology (mean age 55 years). In total, 4 studies were retrospective, one was prospective.
In this review, we assessed MRI data from patients with dural invasion only. Sensitivity ranged from 0.62 to 1.00, and specificity from 0.33 to 1.00, with median values of 0.79 and 0.82, respectively. PPV ranged from 0.61 to 1.00 and NPV from 0.50 to 1.00, had a median of 0.87 and 0.79, respectively.

| Study | Year | N patients | mean age | Sensitivity | Specificity | PPV | NPV |
|---|---|---|---|---|---|---|---|
| McIntyre et al. | 2012 | 39 | 54.6 | 0.93 | 0.33 | 0.87 | 0.50 |
| Salfrant et al. | 2021 | 160 | 57 | 0.79 | 0.82 | 0.61 | 0.92 |
| Meerwein et al. (Reader 1) | 2020 | 73 | 67 | 0.62 | 0.96 | 0.90 | 0.79 |
| Meerwein et al. (Reader 2) | 2020 | 73 | 67 | 0.79 | 0.75 | 0.68 | 0.85 |
| Eisen et al. | 1996 | 4 | 52.3 | 0.67 | 1.00 | 1.00 | 0.50 |
| Geltzeiler et al. | 2023 | 209 | 52.2 | 0.79 | 0.77 | 0.81 | 0.75 |
| Ishida et al. | 2002 | 3 | 47.3 | 1.00 | 1.00 | 1.00 | 1.00 |

Studies included in the systematic review (only patients with MRI features of dural invasion included in the table). PPV, positive predictive value; NPV, negative predictive value

Half of studies used 1.5T, with one additionally using 3T, and half did not report field strength. Most common MRI features were linear or nodular dural enhancement and thickening. Most common tumor localizations were olfactory cleft and ethmoid sinus. Most common tumor histologies were olfactory neuroblastoma and intestinal-type adenocarcinoma.
**Conclusion:** MRI is an essential tool in assessing the intracranial invasion of sinonasal malignancies. Thick and/or nodular dural enhancement was the most commonly found MRI feature. However, the accuracy varies, and the evidence is limited to a few studies. Limitations also include variability in MRI vendors/protocols, imaging criteria, and different reference standards.

### A retrospective cohort study of the radiological features of HPV-positive versus HPV-negative oropharyngeal squamous carcinoma in patients presenting between 2007-2023 at a large tertiary hospital in South Africa

*Authors:* A. Naranbhai, A. Afrogheh, S. O'Hagan, J. Grobbelaar, L. Janse van Rensburg; Cape Town/ZA

**Purpose/Objectives:** To assesses whether patients in our population with HPV positive and HPV negative OPSCC have different CT imaging appearances. Specifically, it assess whether the HPV positive OPSCC cases had smaller primary tumours, more advanced lymph node spread and more frequently cystic lymph nodes[Ref1][Ref2][Ref3].
**Methods and materials:** The study retrospectively recruited all patients with HPV positive OPSCC and a random sample of patients with HPV negative OPSCC who presented between 2007 and 2023. Eligibility required a biopsy proven OPSCC, a p16 antigen test, a contrast enhanced neck CT scan and basic demographic information. These were accessed from hospital and laboratory archives. All p16 positive cases had a confirmatory HPV DNA PCR test[Ref4].
CT scan readers were blind to the p16 and HPV status of the tumour, the patient demographics and prior radiology reports. Scan were read by two readers (a board-certified and a trainee Diagnostic Radiologist) with additional input from a third reader (board certified) during the consensus read.
A formal sample size calculation was performed. Statistical significance could not be reached owing to the low prevalance of HPV positive OPSCC in our population.
**Result:** 21 patients with HPV positive OPSCC and 55 patients with HPV negative OPSCC were recruited.

| | HPV related OPSCC | HPV unrelated OPSCC |
|---|---|---|
| Mean age, years (SD) | 55.1 (9.4) | 58.1 (9.2) |
| Male sex, n (%) | 15 (71.4%) | 43 (78.2%) |
| Ever smoked, n (%) | 10 (47.6%) | 37 (67.3%) |
| Use alcohol, n (%) | 6 (28.6%) | 27 (49.1%) |
| HIV positive, n (%) | 1 (4.8%) | 5 (9.1%) |
| Mean tumour diameter, mm (SD) | 40.9 (18.6) | 39.2 (19.6) |
| Mean lymph node diameter, mm (SD) | 26.4 (18.7) | 20.0 (18.2) |
| Bilateral or more lymph node extension, n (%) | 12 (47.6%) | 28 (50.9%) |
| Cystic lymph nodes, n (%) | 2 (9.5%) | 2 (3.6%) |

Clinical and radiological characteristics of patients

There was no meaningful difference in the mean size of the primary tumour (41mm vs 39mm, respectively), the mean size of the largest lymph node (26mm vs 20mm) or the extent of lymph node spread (bilateral or more lymph nodes in 48% vs 51%). Cystic lymph nodes were seen in 2 (10%) of the HPV positive patients and 2 (4%) of the HPV negative patients. Presence of metastasis was similar in both groups (15% and 18% respectively).

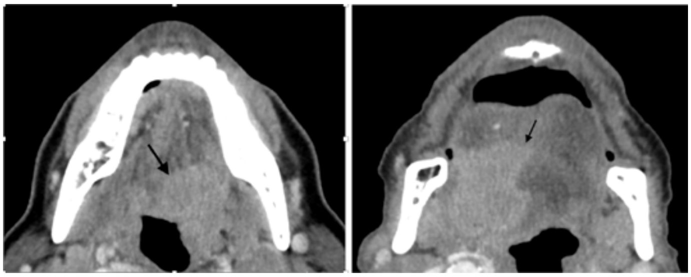

Axial contrast enhanced neck CT showing (a) An HPV positive and (b) an HPV negative base of tongue tumour. Note the similar appearance of both in these examples.

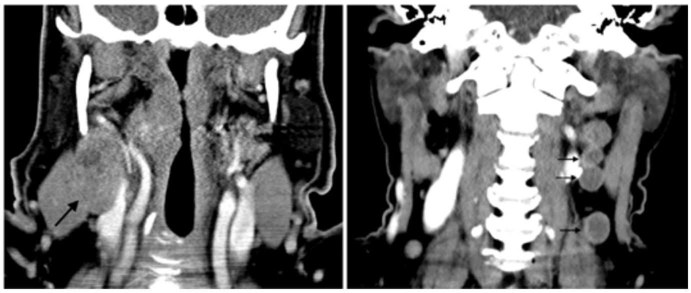

Coronal contrast enhanced neck CT images showing (a) Cystic and (b) solid lymph node spread. Cystic lymph nodes were uncommon in both cancer types.

Both groups had a no significant difference in gender (70% and 78% male), age (55 and 58 years), HIV status (5% vs 9%) and employment status (33% vs 24%). A history of smoking (48% vs 67%) or alchohol use ((29% vs 49%) was less common in the HPV positive group.
**Conclusion:** This study showed no meaningful difference in the CT imaging appearance of HPV positive and HPV negative OPSCC in our population. Furthermore, the HPV positive cases did not demonstrate smaller primary tumours, more advanced lymph node spread and more frequent cystic lymph node spread. Both groups of patients had a similar demographic profile however a history of smoking or alchohol use was less common in the HPV positive group. As per international guidelines, radiological features are unable to diagnose an OPSCC as being due to HPV in our population.

References:
[Ref1] Huang et al., (2017), Cystic nodal metastasis in patients with oropharyngeal squamous cell carcinoma receiving chemoradiotherapy: Relationship with human papillomavirus status and failure patterns, PLOS ONE, e0180779, 7, https://dx.plos.org/10.1371/journal.pone.0180779, 2022-01-22
[Ref2] Goldenberg et al., (2008), Cystic lymph node metastasis in patients with head and neck cancer: An HPV-associated phenomenon, Head & Neck, 898-903, 7, https://onlinelibrary.wiley.com/doi/full/10.1002/hed.20796 https://onlinelibrary.wiley.com/doi/abs/10.1002/hed.20796 https://onlinelibrary.wiley.com/doi/10.1002/hed.20796, 2022-01-22
[Ref3] Cantrell et al., (2013), Differences in Imaging Characteristics of HPV-Positive and HPV-Negative Oropharyngeal Cancers: A Blinded Matched-Pair Analysis, American Journal of Neuroradiology, 2005-2009, 10, http://www.ajnr.org/lookup/doi/10.3174/ajnr.A3524, 2023-01-22
[Ref4] Dapaah et al., (2021), The Prevalence of Human Papillomavirus–Positive Oropharyngeal Squamous Cell Carcinoma at One of the Largest Tertiary Care Centers in Sub-Saharan Africa, Archives of Pathology & Laboratory Medicine, https://meridian.allenpress.com/aplm/article/doi/10.5858/arpa.2021-0021-OA/474697/The-Prevalence-of-Human-Papillomavirus-Positive, 2022-01-22

### Brachial Plexopathy: Clinical-Radiological Patterns in Breast Cancer

*Authors: E. Contrino[1], A. Napolitano[2], C. Mandelli[3], A. Lo Casto[1], S. Gerevini[2];*
*[1]Palermo/IT, [2]Bergamo/IT, [3]Milano/IT*

**Purpose/Objectives:** Brachial plexopathy is a rare but significant complication that causes pain and disability in cancer patients. The causes of brachial plexopathy in cancer patients are tumor infiltration, metastatic spread, radiation damage, trauma to the plexus during surgery or anesthesia, and acute brachial neuritis.
Breast cancer is the most commonly diagnosed malignancy in women worldwide (nearly 1.6 million new cases diagnosed each year); in Italy there are approximately 48,000 new cases each year with a continuously increasing incidence.
Frequently in patients with breast cancer, brachial plexopathy occurs following metastases to the brachial plexus and after radiation therapy.
Brachial plexopathy can have significant functional consequences, such as pain, upper extremity weakness, limitation of shoulder movement, or associated malignant lymphedema.
In this study we will report different neuroradiological patterns of causes of brachial plexopathy in patients with breast cancer.
**Methods and materials:** We retrospectively examined brachial plexus MRI of patients with breast cancer performed at the Neuroradiology Unit of the ASST Papa Giovanni XXIII in Bergamo from 2019 to 2023. Several neuroradiological patterns associated with brachial plexopathy were identified. The main available clinical and pathological details of the primary breast tumor diagnosis were included, including laterality of the disease, age and diagnostic data, tumor histology and grade, and treatments. Data were also collected regarding information on brachial plexopathy, including: laterality of involvement, initial symptoms, data on the onset of initial symptoms, symptoms present on clinical evaluation, previous surgeries and treatments. When available, other diagnostic tests including electromyography (EMG) and PET/CT were also collected.
**Result:** Several clinical patterns associated with brachial plexopathy were selected, identified thanks to MR neurography of the brachial plexus including: direct perineural tumor spread, metastatic invasion, radiation-induced neuropathy, iatrogenic neuropathy following surgery or anesthetic procedure and paraneoplastic neuropathy.
**Conclusion:** Our investigation revealed how brachial plexopathy in patients with breast cancer can be associated with different clinical features and present with different neuroradiological patterns. Although patients with brachial plexopathy are often in advanced stages of breast cancer at the time of diagnosis, these findings can help medical doctors to detect signs and symptoms of brachial plexopathy and plan further diagnostic evaluations at an early stage (ENG/EMG; MRI of the brachial plexus) in order to provide adequate treatment and potentially have a better prognosis.

### Retrospective analysis and imaging review of laryngeal and hypopharyngeal carcinoma by a structured reporting checklist and its impact on staging and management in a high-volume tertiary care hospital

*Authors: M. S. Swarup, H. Sharma, G. Sindhwani, R. Gupta Ghasi; New Delhi/IN*

**Purpose/Objectives:** Laryngeal and hypopharyngeal carcinoma is quite prevalent in Asian region with multiple risk factors working in tandem. CECT and contrast enhanced MRI are working modalities in diagnosis, assessment of lesion size/extent, invasion of adjacent structures, and loco-regional staging. However, complex laryngeal anatomy, variable growth pattern of malignancy, involvement of solitary or multiple sub-compartments of larynx, extension to/from hypopharynx subsites and oropharynx present a real challenge for complete assessment of loco regional extent of laryngeal carcinoma and proper radiologic staging, particularly in a high volume general tertiary care hospital. We try to evaluate effectiveness of a systematic comprehensive reporting checklist in accurate staging of laryngeal carcinoma.
**Methods and materials:** We retrospectively evaluated the images of 65 biopsy-proven cases of squamous cell carcinomas of various subsites of larynx and hypopharynx. The available images were CECT and MRI of the face and neck regions. We included both pretreatment cases done for primary staging of disease as well as post-treatment cases (only post radiotherapy/ chemotherapy) for restaging of disease/ response assessment. We prepared a systematic and comprehensive checklist for reporting in lines of AJCC TNM staging ($8^{th}$ ed) and evaluated its role in minimizing the pitfalls in staging of laryngeal carcinoma. We will also discuss imaging pattern of spread and appearance of various stages of laryngeal carcinoma highlighting potential pitfalls and measures how to avoid them. Special emphasis will be given for involvement of laryngeal cartilages.
**Result:** The initial T-staging was found to be inaccurate in 18 % of cases (12 out of 65 cases) on review. Understaging was observed due to inaccuracy in delineating involvement of preepiglottic/ paraglottic fat spaces, outer cortex of thyroid cartilage, post cricoid region, esophagus and prevertebral space. Overstaging was observed in estimation of cartilage invasion on CT and extralaryngeal spread on MRI. The proposed systematic reporting checklist is more effective in reducing the pitfalls in accurate staging and also reduced the review request at multidisciplinary meetings due to better communication of all clinically relevant imaging findings with the referring surgeons/ radiation oncologists.
**Conclusion:** Familiarity with pattern of spread of laryngeal and hypopharyngeal carcinoma of different subsites, spectrum of imaging morphology of various stages along with utilization of a systematic reporting checklist is essential for eliminating potential pitfalls, providing proper staging of disease and thereby guiding optimal patient management, particularly in a general tertiary care hospital with high caseloads and busy workflow.

### Odontogenic neck abscesses caused by Streptococcus anginosus group (SAG): Emergency MRI findings

*Authors: J. Hirvonen[1], M. Lääveri[2], J. Heikkinen[2], T. Happonen[2], A. Sirén[2], J.-P. Vierula[2], J. Velhonoja[2], H. Irjala[2], T. Soukka[2], K. Mattila[2], M. Gunell[2], J. Snäll[3], J. Nurminen[2]; [1]Tampere/FI, [2]Turku/FI, [3]Helsinki/FI*

**Purpose/Objectives:** Streptococcus (Str.) anginosus group (SAG, including Str. anginosus, Str. intermedius, and Str. constellatus) has recently been acknowledged as a group of pathogens associated with a particularly severe illness in patients with odontogenic neck abscesses [1]. Magnetic resonance imaging (MRI) offers excellent diagnostic accuracy, precise measurements of abscess dimensions, and demonstration of edema patterns with prognostic significance [2][3][4]. We sought to explore whether abscesses caused by SAG are associated with distinct MRI findings.
**Methods and materials:** This retrospective chart review had the following inclusion criteria: 1) MRI evidence of an acute odontogenic neck infection and an abscess in 2013-2021; 2) surgical confirmation of an abscess; 3) microbiological cultures from intraoperative pus samples. From the MR images, we measured abscesses and determined specific edema patterns as previously described: retropharyngeal edema (RPE), mediastinal edema (ME), submandibular space edema (SMSE), sublingual space edema (SLSE), and visceral space edema (VSE). All of the MRI outcomes have been shown to have good interobserver agreement. SAG positive and negative patients were compared in terms of MRI findings and the following clinical variables: C-reactive protein (CRP), white blood cell count (WBC), and need for transcervical (in addition to intraoral) surgery. Data were analyzed using univariate models (Chi-square tests and t-tests) and a multivariate binary logistic regression model. P values less than 0.05 were considered statistically significant.
**Result:** We found 93 patients fulfilling the inclusion criteria. Among these patients, 47 (51%) had SAG growing in their cultures, whereas 46 (49%) did not. Among the MRI findings, the SAG-positive patients had a higher prevalence of ME (47% vs. 16%, p=0.003), whereas other imaging and clinical predictors (abscess diameter, RPE, SMSE, SLSE, VSE, CRP, WBC, symptom duration, prior procedures) were not significantly different. Regarding surgical outcomes, SAG-positive patients required transcervical incisions more often than SAG-negative patients (62% vs. 32%, p=0.004). In the multivariate model, ME was the only statistically significant predictor of SAG positivity (OR=7.3, p=0.003).

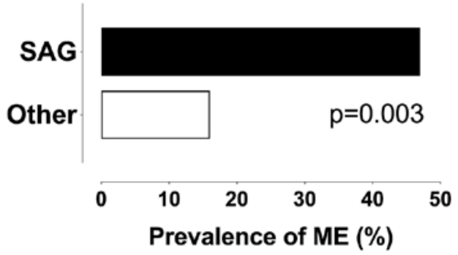

Prevalence of mediastinal edema (ME) on emergency MRI in SAG-positive vs. negative cases.

**Conclusion:** In patients with odontogenic neck abscesses, mediastinal edema seen on emergency MRI is highly suggestive of the involvement of SAG, which has been shown to be associated with a severe course of illness. These findings highlight the clinical utility of emergency MRI in patients with acute neck infections

References:
[1] Furuholm J, Uittamo J, Rautaporras N, Välimaa H, Snäll J, (2023), Streptococcus anginosus: a stealthy villain in deep odontogenic abscesses, Odontology, 522-530, 111
[2] Heikkinen J, Nurminen J, Velhonoja J, Irjala H, Happonen T, Mattila K, Hirvonen J, (2022), Clinical and prognostic significance of emergency MRI findings in neck infections, European Radiology, 1078-1086
[3] Heikkinen J, Jokihaka V, Nurminen J, Jussila V, Velhonoja J, Irjala H, Soukka T, Happonen T, Järnstedt J, Nyman M, Mattila M, Hirvonen J, (2023), MRI of odontogenic maxillofacial infections: diagnostic accuracy and reliability, Oral Radiology, 364-371, 39
[4] Hirvonen J, Heikkinen J, Nyman M, Happonen T, Velhonoja J, Irjala H, Soukka T, Mattila K, Nurminen J., (2023), MRI of acute neck infections: evidence summary and pictorial review, Insights into Imaging, 5, 14

### Pushing the limits: Next-generation AI-based reconstruction algorithms in ultra-high resolution CT for head and neck imaging, further dose reduction and image optimization

*Authors:* *D.A. Messerle, N.F. Grauhan, A.E. Othman, M.A. Brockmann, P. Roman, A. Kronfeld, L. Leukert, A.-K. Dapper, S. Altmann; Mainz/DE*

**Purpose/Objectives:** Deep learning-based image reconstruction has become a valuable tool in reconstructing acquired CT data, improving image resolution and quality. Recently, a new generation of reconstruction algorithms has been developed. We investigated the effects of different reconstruction algorithms, weight-adapted kV reduction, and tube current modulation on image quality and radiation exposure.

**Methods and materials:** Currently, 48 consecutive patients with head and neck UHR-CT were retrospectively evaluated. Patients were examined using tube current modulation and body weight-adapted tube voltage (80 kV ≤70 kg, 100 kV >70 and ≤80 kg and 120 kV >80 kg). Images were reconstructed using adaptive iterative dose reduction (AIDR) and advanced intelligent Clear-IQ engine (AICE), using body reconstruction algorithm (AICE-B) and CTA reconstruction algorithm (AICE-CTA). Three readers evaluated subjective image quality using a 5-point Likert scale regarding image quality, diagnostic acceptability, and assessment of several important anatomic regions. Objective image quality was evaluated by selecting slices of temporalis and masseteric muscle and the floor of the mouth in every patient. Values for signal intensity (SI), image noise, entropy, signal-to-noise ratio (SNR) and contrast-to-noise ratio (CNR) were calculated. Radiation dose was evaluated by comparing the CTDI values.

**Result:** The current study sample consisted of 48 patients (mean age 66.50 +/- 15 years, 25 males and 23 females). Both reconstruction algorithms significantly improvement subjective image quality (e.g. diagnostic acceptability: AICE-B vs AIDR with OR of 43.5 [CI: 21.7;87.3], p <0.001 and AICE-CTA vs AIDR >999.9 [CI: 701.6;>999.9], p <0.001) with excellent image quality for AICE-CTA (70.1% excellent diagnostic acceptability for AICE-CTA vs 79.2% good for AICE-B and 86.1% moderate for AIDR). Similar results were demonstrated for objective image quality (SNR: p < 0.000; CNR: p < 0.025). AICE-CTA significantly outmatched body-reconstruction algorithm for all defined parameters and regions (e.g. image noise, masseteric muscle with 14.04 [AIDR] vs. 13.04 [AICE-B]: p =0.030 and with 5.89 [AICE-CTA]: p < 0.001; CNR with 12.24 [AIDR] vs. 13.38 [AICE-B]: p =0.059 and with 27.83 [AICE-CTA]: p < 0.001), showing similar results for all three anatomic locations. Using weight-adapted kV reduction with tube current modulation while simultaneously applying AICE, very low radiation doses could be achieved (CTDI: 7.1 +/- 2.8), undercutting current diagnostic reference values by more than 50%.

**Conclusion:** Employing AI-based reconstruction algorithms in ultra-high resolution head and neck imaging ensures high image quality while achieving very low radiation exposure, thereby enhancing the scope of diagnostic imaging.

### Prediction of intensive care unit (ICU) admissions in acute neck infections: Classification performance of an MRI-based risk score

*Authors:* *J. Hirvonen[1], J.-P. Vierula[2], J. Heikkinen[2], T. Happonen[2], A. Sirén[2], J. Velhonoja[2], H. Irjala[2], T. Soukka[2], K. Mattila[2], J. Nurminen[2]; [1]Tampere/FI, [2]Turku/FI*

**Purpose/Objectives:** In acute neck infections, magnetic resonance imaging (MRI) affords unsurpassed diagnostic accuracy, precise measurements of abscess dimensions, and demonstration of specific edema patterns with prognostic significance [1][2]. We created and tested a risk score to predict intensive care unit (ICU) admissions in a large sample of patients with acute neck infections imaged with MRI.

**Methods and materials:** In this retrospective cohort study, we included patients who had: 1) MRI evidence of an acute neck infection imaged in 2013-2021; 2) surgical confirmation of an abscess, if shown on MRI; 3) relevant clinical and laboratory information available from medical records. From the MR images, we measured abscesses (patients without abscesses were denoted zero) and determined specific edema patterns as previously described: retropharyngeal edema (RPE) and mediastinal edema (ME). The following clinical and laboratory values were recorded: age, sex, C-reactive protein (CRP), white blood cell count (WBC), and the need for ICU. Associations between predictor variables and ICU admissions were initially analyzed using a multivariate binary logistic regression model, and risk score coefficients were derived from regression coefficients. Model performance was evaluated using areas under the curve (AUC) of receiver operating characteristic (ROC) analyses. P values less than 0.05 were considered statistically significant.

**Result:** The final sample included 535 patients (57% male, 43% female, mean age 41 years), of whom 373 (70%) had an abscess, and 62 (12%) required ICU treatment. In the logistic regression model, RPE (p=0.003), maximal abscess diameter (p=0.001), and CRP (p=0.001) were significant predictors. Cut-off points were >39.5 mm for maximal abscess diameter and >171.5 mg/l for CRP. Risk scores (0-7) were then calculated as 3*RPE + 2*(high CRP) + 2*(large abscess) using the cut-off points for continuous variables. In the ROC analysis, the composite risk score (AUC=0.82) outperformed CRP (AUC=0.74, p=0.03), abscess diameter (AUC=0.72, p=0.04), and RPE (AUC=0.71, p=0.004). Discriminative performance of the risk score at a cut-off >3 points yielded sensitivity 66%, specificity 82%, positive predictive value 33%, negative predictive value 95%, accuracy 80%, and odds ratio 9.0 (p<0.001).

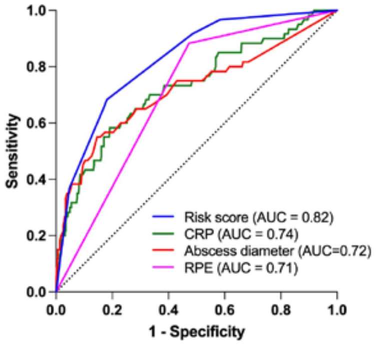

ROC curves for predictors of ICU treatment.

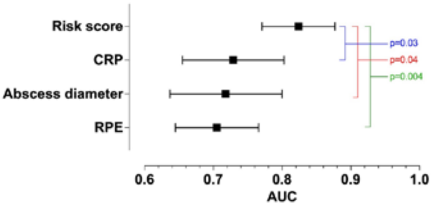

ROC AUC values, their 95% confidence intervals, and statistical comparisons againg the composite risk score.

**Conclusion:** A risk model based on RPE, maximal abscess diameter, and CRP achieved moderate overall accuracy (80%) and high negative predictive value (95%). These findings add to the growing evidence for the utility of MRI in acute neck infections. A practical implication is that a patient with no more than one of these risk factors is very unlikely to require ICU treatment.

References:
[1] Heikkinen J, Nurminen J, Velhonoja J, Irjala H, Happonen T, Mattila K, Hirvonen J, (2022), Clinical and prognostic significance of emergency MRI findings in neck infections, European Radiology, 1078-1086
[2] Hirvonen J, Heikkinen J, Nyman M, Happonen T, Velhonoja J, Irjala H, Soukka T, Mattila K, Nurminen J., (2023), MRI of acute neck infections: evidence summary and pictorial review, Insights into Imaging, 5, 14

### An international survey of current practice in diffusion and perfusion imaging of head and neck

*Authors:* S. Connor[1], A. Christoforou[1], K. Mosier[2], A. Srinivasan[3],
on behalf of ESHNR and ASHNR diffusion and perfusion imaging survey group;
[1]London/GB, [2]Indianapolis/US, [3]Ann Arbor/US

**Purpose/Objectives:** The application of diffusion and perfusion imaging in the head and neck has been widely reviewed in the literature and in educational programmes. Despite this, it remains unclear how these sequences are currently being applied in routine clinical work. A survey was undertaken to help us to better understand when and how these techniques are practiced by head and neck radiologists worldwide, and whether there are any barriers to their implementation.
**Methods and materials:** A core representation of head and neck subspecialty radiologists was assembled from the ASHNR and the ESHNR. Through an iterative review process, a questionnaire was formulated to focus on the clinical application, analysis, and reporting of qualitative DWI (DWI), quantitative MRI (qDWI) and DCE perfusion MRI (DCE-PWI) in the head and neck, as well as potential obstacles to their application. The survey was distributed to ESHNR (n=726) and ASHNR (n=535) members on 3 occasions and was open to responses for a 3-month period.
**Result:** Pending a final survey distribution, there are currently provisional results from 258 responses. There were >80% of respondents from institutions routinely acquiring head and neck DWI. Almost all (98%) of these performed qualitative but only a minority (<40%) performed qDWI evaluation. Qualitative DWI was routinely interpreted across a broad range of clinical indications with the majority using it to diagnose cholesteatoma, distinguish tumour histology, diagnose malignancy, identify abscess and distinguish recurrent tumour. qADC was most frequently used to distinguish different types of tumour histology (mucosal based or salivary) or recurrent carcinoma. Head and neck DCE-PWI was performed in the majority (55%) of respondents' institutions however it was rarely (<20%) routine. There was variation in the methods of analysis and reporting of DCE-PWI with the majority reporting classifications of time intensity curves. Lack of standardisation across centres was identified to be a key reason for not implementing qMRI whilst there were a wide range of factors impeding the adoption of DCE-PWI. There was a strong interest (80%) in initiating these techniques if provided with further resources and education.
**Conclusion:** Provisional outcomes from this international survey of diffusion and perfusion imaging in the head and neck highlight the widespread interpretation of qualitative DWI but considerable variation in the application and analysis of quantitative advanced MRI techniques. This survey aids discussion on how to standardise optimal practice and to justify the provision of targeted education and resources to professional and health care organisations.

### Comparing Ultrasound-based TIRADS and BETHESDA Scoring Systems in The Preoperative Diagnosis of Thyroid Nodules

*Authors:* S. Assiri, Z. Almalki, F.S. Alnemari, F. Altalhi, M. Alnemari, E. Aljuaid, T. AlGhamdi, S. Elsiddeeg; Taif/SA

**Purpose/Objectives:** The TIRADS (Thyroid Imaging Reporting and Data System) aids in the stratification of the malignancy risk. [1],[2] Due to the commonality of thyroid nodules and the fact that most thyroid nodules are benign, it is essential to be highly selective of patients who will undergo surgical excisions to prevent unnecessary invasive interventions. [3] TIRADS system has not been incorporated into the ATA (American Thyroid Association) guidelines as of yet. However, ATA recommends using the Bethesda system for reporting the results of patients who have a Fine Needle Aspiration Cytology (FNAC). [4] Thyroid cancer incidence in Saudi Arabia has increased exponentially in the past decade. [5] We decided to conduct the current study due to the scarcity of articles published from Saudi Arabia that compare US TIRADS, the Bethesda system, and the final histopathology of resected thyroid nodules.
**Methods and materials:** A retrospective study that included all patients at Alhada Armed Forces Hospital from January 2017 to January 2023 who had thyroid nodules and underwent ultrasound results classified with the TIRADS system followed by FNAC results classified with the Bethesda system then underwent a complete surgical excision by undergoing hemithyroidectomy or total thyroidectomy with a final histopathological result. We used the numerical American College of Radiology Thyroid Imaging Reporting and Data Systems (ACR-TIRADS) for ultrasound imaging of thyroid nodules. All patients had a subsequent FNAC classified with the 2023 Bethesda System for Reporting Thyroid Cytopathology.
**Result:** A total of 234 patients, most of them were female. 18.4% of the patients had a nodule on the right side,17.5% on the left side, and the rest 64.1% on both sides. Table.1 shows the Socio-demographic characteristics and risk factors among our patients.

| | Mean | ± sd | | N | (%) | | N | (%) |
|---|---|---|---|---|---|---|---|---|
| Age | 46.22 | 12.65 | Female | 204 | 87.2 | Diabetes | 47 | 20.1 |
| Height (cm) | 157.7 | 7.09 | Male | 30 | 12.8 | Hypertensi on | 43 | 18.4 |
| Weight (kg) | 77.2 | 16.3 | History of exposure to radiation | 3 | 1.3 | Hypothyroi dism | 41 | 17.5 |
| BMI | 31.05 | 6.34 | Family history of thyroid cancer | 29 | 8.2 | Hyperpara thyroidism | 9 | 3.8 |
| Size of Right nodules (cm) | 4.19 | 2.21 | Personal history of thyroid cancer | 4 | 1.7 | Asthma | 14 | 6 |
| Size of Left nodules (cm) | 3.86 | 2.4 | No Previous hemithyrio ectomy surgery | 227 | 97 | Smoker | 5 | 2.1 |
| Duration of symptoms (months) | 11.58 | 12.91 | Accelerate d growth of the thyroid nodules | 59 | 25.2 | Non-smoker | 227 | 97 |
| Duration of Lump (year) | 2.73 | 4.06 | Lymph Node Enlargem ent | 48 | 20.5 | Ex-smoker | 2 | 0.9 |

Table 1 Socio-demographic characteristics and risk factors for thyroid cancer

The categorization frequency of TIRADS and Bethesda scores are demonstrated in Table 2

| | TIRADS 1 | TIRADS 2 | TIRADS 3 | TIRADS 4 | TIRADS 5 |
|---|---|---|---|---|---|
| **Bethesda I (n=9)** | | 1 (11.1%) | 2 (22.2%) | 3 (33.3%) | 3 (33.3%) |
| **Bethesda II (n=144)** | 10 (6.9%) | 16 (11.1%) | 16 (11.1%) | 65 (45.1%) | 37 (25.7%) |
| **Bethesda III (n=19)** | 2 (10.5%) | 2 (10.5%) | | 9 (47.4%) | 6 (31.6%) |
| **Bethesda IV (n=22)** | 3 (13.6%) | 5 (22.7%) | 3 (13.6%) | 10 (45.5%) | 1 (4.5%) |
| **Bethesda V (n=22)** | | | 1 (4.5%) | 8 (36.4%) | 13 (59.1%) |
| **Bethesda VI (n=18)** | | 1 (5.6%) | 3 (16.7%) | 5 (27.8%) | 9 (50%) |

Table 2 TIRADS and Bethesda categorization frequency

Performance measures of TIRADS and Bethesda as diagnostic tests are highlighted in Table 3

| | Sensitivity (%) | Specificity (%) | PPV | NPV | Accuracy |
|---|---|---|---|---|---|
| TIRADS | 88.6 | 34.8 | 36.7 | 87.7 | 50.9 |
| Bethesda | 62.9 | 89 | 71 | 84.89 | 81 |

Table 3 Performance measures of TIRADS and Bethesda as diagnostic tests

Only half of the patients with TIRADS V nodules were malignant on final histopathology, on the other hand, 92.5% of Bethesda V or VI nodules were malignant on final histopathology with a P-value of <0.001. Table 4

| TIRADS score | Malignant Histopathology | Benign Histopathology | Total |
|---|---|---|---|
| III and IV | 31 | 94 | 125 |
| V | 35 | 34 | 69 |
| Total | 66 | 128 | 194 |
| Bethesda score | Malignant Histopathology | Benign Histopathology | Total |
| III and IV | 12 | 29 | 41 |
| V and VI | 37 | 3 | 40 |
| Total | 49 | 32 | 81 |

Table 4 Comparison between TIRADS and Bethesda scores and final histopathology of thyroid nodules

**Conclusion:** Bethesda classification system is significantly better in accurately diagnosing malignant nodules when compared to TIRADS system. Our study confirms that the TIRADS system has a considerable utility in triaging patients with thyroid nodules and it has a decent correlation with Bethesda score and the final surgical histopathology. A holistic view of the patient's history and risk factors should be considered for patients with low TIRADS score before differing FNAC or further surgical management.

References:
[1] Horvath, E., Majlis, S., Rossi, R., Franco, C., Niedmann, J. P., Castro, A., & Dominguez, M., (2009), An ultrasonogram reporting system for thyroid nodules stratifying cancer risk for clinical management., The Journal of clinical endocrinology and metabolism, https://pubmed.ncbi.nlm.nih.gov/19276237/
[2] Mendes, G. F., Garcia, M. R., Falsarella, P. M., Rahal, A., Cavalcante Junior, F. A., Nery, D. R., & Garcia, R. G., (2018), Fine needle aspiration biopsy of thyroid nodule smaller than 1.0 cm: accuracy of TIRADS classification system in more than 1000 nodules, The British journal of radiology, https://pubmed.ncbi.nlm.nih.gov/29182368/
[3] Giles, W. H., Maclellan, R. A., Gawande, A. A., Ruan, D. T., Alexander, E. K., Moore, F. D., Jr, & Cho, N. L., (2015), False negative cytology in large thyroid nodules, Annals of surgical oncology, https://pubmed.ncbi.nlm.nih.gov/25074665/
[4] Haugen BR, Alexander EK, Bible KC, Doherty GM, Mandel SJ, Nikiforov YE, Pacini F, Randolph GW, Sawka AM, Schlumberger M, Schuff KG, Sherman SI, Sosa JA, Steward DL, Tuttle RM, Wartofsky L., (2016), 2015 American Thyroid Association Management Guidelines for Adult Patients with Thyroid Nodules and Differentiated Thyroid Cancer: The American Thyroid Association Guidelines Task Force on Thyroid Nodules and Differentiated Thyroid Cancer., Thyroid, https://www.ncbi.nlm.nih.gov/pmc/articles/PMC4739132/
[5] Flemban, A. F., Kabrah, S., Alahmadi, H., Alqurashi, R. K., Turaes, A. S., Almaghrabi, R., Al Harbi, S., & Khogeer, A. A. , (2022), Patterns of Thyroid Cancer Mortality and Incidence in Saudi Arabia: A 30-Year Study, Diagnostics, https://pubmed.ncbi.nlm.nih.gov/36359559/

### Dissection of the internal carotid artery related to styloid process: New observational data, systematic review and meta-analysis

*Authors: T. Klail[1,2], L. Sachs[1], L. Panos[1], O.Y. Urban[1], T. Siller[1], S. Pilgram-Pastor[1], M. Müller[1], R. Giger[1], F. Wagner[1]; [1]Bern/CH, [2]Brno/CZ*

**Purpose/Objectives:** Internal carotid artery dissection (ICA-D) frequently leads to stroke in individuals under 50. Its causes, often clear, sometimes remain mysterious, labeled as 'spontaneous' for a portion of patients without a known origin. Trauma also contributes to ICA-D. The involvement of the styloid process (SP) in dissections, despite growing evidence, is still underacknowledged.
**Methods and materials:** Comprehensive literature searches were performed in MEDLINE and EMBASE databases to identify all articles pertaining the matter of ICA-D linked to an ipsilaterally elongated SP with short ICA-SP distance. Additionally, we performed a meta-analysis of the available data on the relationship between short SP-ICA distance and the rate of ICA-D. We also present on overview of the diagnostic and treatment modalities employed on selected patients with suspected ICA-D related to SP under our care.
**Result:** In our institute, during the last 4 months, four patients have been identified with spontaneous or traumatic dissections of the ICA, particularly related to the SP. This prompts us to consider the possibility of an underdiagnosis of the styloid process as potential cause of an ICA-D over recent years. Over 70 case reports and several further case-control studies have been published, increasing confirmatory evidence on this pathology and proving the role of SP-length and SP-ICA distance in patients with ICA-D.
**Conclusion:** SP emerges as a plausible causative factor in ICA-D with potential life-threating consequences. In cases where the etiology of the dissection remains unclear, especially in emergency scenarios, it is imperative to include an assessment of the stylohyoid process. An early diagnosis of ICA-D is essential for initiating the appropriate treatment and interdisciplinary management of these patients.

### Blind spots on CT imaging of head and neck

*Authors: V. Nechaev, A. Vasil'ev; Moscow/RU*

**Purpose/Objectives:** Determine the most common anatomical areas of the head and neck where radiologists do not describe pathological changes when analyzing computed tomograms.
**Methods and materials:** We retrospectively analyzed 62 CT scans of the head and neck area which were performed for the primary assessment of the oncological process of various localizations and in which there was no description of additional pathological changes during the initial interpretation. These changes could affect the patient's treatment strategy or require consultation with related specialists or additional examination. All cases were verified after repeated radiological examination in 77% of cases or during retrospective analysis in 23% of cases. The time from the initial examination to detection of missed pathology was on average 119 days.
**Result:** As a result several anatomical zones were identified in which pathological errors were not initially described: intracranial (n=5; 7.8%), paranasal sinuses (n=8; 12.9%), brachiocephalic vessels (n=15; 24,2%), parotid salivary glands (n=10; 16,1%), soft tissues of the neck (n= 4; 6.5%), upper parts of the lungs and mediastinum (n=9; 14,6%), temporal bones (n=5; 8,1%), thyroid gland (n=3; 4.8%) and cervical spine (n=3; 4.8%),
There was no description of the pathology of the brachiocephalic arteries, predominantly hemodynamically significant stenoses of the internal carotid arteries (n=13) and aneurysm (n=2), lesions of the major salivary glands (n=10), metastatic lymph nodes (n=4) and lesions in the thyroid gland (n=3). In the upper parts of the lungs there were no information of a primary or secondary oncological process (n=6), and in the upper mediastinum - an aortic aneurysm (n=1) or lymphadenopathy (n=2). In the paranasal sinuses and temporal bones were unnoticed predominantly inflammatory changes (n=12) and mass (n=1). In cervical spine there was no description of metastasis (n=3).
**Conclusion:** The most common "blind spots" on CT of the head and neck region have been identified. Knowledge of them can potentially lead to a reduction in the incidence of missed pathology when interpreting CT study of this anatomical location.

### The clinical utility of contrast enhanced MRI in the investigation of burning mouth syndrome

*Authors: B. Vekaria, S. Connor; London/GB*

**Purpose/Objectives:** Burning mouth syndrome is characterised by the sensation of burning within the oral cavity and lips in the absence of a medical or dental cause. It can often be accompanied with a variety of other symptoms including altered taste or oral dryness and is more commonly seen in women [26745781] [35574619]. MR imaging of the brain, skull base, face, and neck is often performed to diagnose clinically occult lesions. We aim to assess the diagnostic yield of gadolinium enhanced MRI neck/skull base in this cohort.
**Methods and materials:** Retrospective analysis of gadolinium enhanced MR imaging of the neck/skull base in patients who presented with burning mouth symptoms was performed from a single centre over a 5-year period (1st October 2018 – 30th September 2023).
Important findings were noted and any follow up or management was correlated with clinical notes. All unrelated incidental findings were documented and classified as major, moderate or minor based on guidelines from the Royal College of Radiologists.
**Result:** A total of 74 patients had contrast enhanced MRIs for symptoms of burning mouth, of these, 58 patients also had brain MRI. The mean age was 53 (range 22-88). The cohort consisted of 61 females and 13 males. The diagnostic yield of imaging for burning tongue for upper aerodigestive tract malignancy was 0% in all groups.
There was a 2.7% diagnostic yield of major unrelated incidental findings (2 patients), a partially occlusive chronic venous sinus thrombosis and a contralateral salivary gland tumour, both of which received follow up and treatment.
7 patients (9%) were found to have moderate incidental findings – defined as indeterminate risk but requiring follow up imaging or tests. For example, pituitary cysts, small intracranial aneurysms and possible meningoceles.
**Conclusion:** The likelihood of causative findings of burning mouth syndrome requiring contrast MRI in patients with burning tongue in our centre over a 5-year period was 0%. There was a low diagnostic yield for major incidental findings (2.7%). The main management impact of contrast imaging was in ruling out a causative lesion so patients could be referred on for conservative management of their symptoms.

References:
[26745781] Zakrzewska J, Buchanan JA., (2016), Burning mouth syndrome, BMJ Clin Evid., 1301, https://www.ncbi.nlm.nih.gov/pmc/articles/PMC4704679/pdf/2016-1301.pdf
[35574619] Russo M, Crafa P, Guglielmetti S, Franzoni L, Fiore W, Di Mario F., (2022), Burning Mouth Syndrome Etiology: A Narrative Review, J Gastrointestin Liver Dis. , 223-228, 31(2), https://www.jgld.ro/jgld/index.php/jgld/article/view/4245/1825

### Comparison between multiparametric MRI and US with SWE (Shear Wave Elastography) in the diagnosis of parotid tumors

*Authors: V. Pampalone[1], F. Cordaro[1], F. Pandolfo[2], A. Inzerillo[1], F. Bencivinni[1], R. Cannella[1], A. Lo Casto[1]; [1]Palermo/IT, [2]Marsala/IT*

**Purpose/Objectives:** Diagnosis of parotid lesions on imaging is sometimes difficult to define, due to their cytoarchitecture, causing overlapping features between benign and malignant lesions. In the literature there are few studies regarding the use of US elastography in the diagnosis of parotid lesions and none of these studies used both MRI and multiparametric US. The purpose of this study was to compare MRI and US with SWE (USWE) in the diagnosis of parotid lesions.
**Methods and materials:** From October 2022 to February 2024, 22 patients (13 females, 9 males, age range 15-82 years), with parotid lesions (size 8-41 mm) were studied. The patients underwent both an USWE (B-mode with a 5–14-MHz linear probe) and an MRI (3T magnet) study of the parotid glands. TSE T1 and T2, STIR, DWI, FFE T1 3D fat sat, before and after i.v. administration of a paramagnetic contrast medium, in a dynamic fashion, sequences were acquired for the MRI study. Time/intensity curves (TIC) were elaborated. All patients underwent US guided biopsy, with 20/22 patients submitted to surgical removal of parotid lesions with definitive histology.
**Result:** 26 lesions were detected at MRI, of which 20/26 were diagnosed as benign (5 pleomorphic adenomas, 11 cystadenolymphoma, 3 intraparotid lymph nodes, 1solitary fibrous tumor) on both MRI and USWE. The benign lesions on USWE showed a 34,61 kPa average stiffness value and a hypoechoic appearance on US. KPa average stiffness value for pleomorphic adenomas was 40,6 while for cystadenolymphoma was 37,3.
6/26 lesions (1 myoepithelial carcinoma, 1 salivary duct carcinoma, 2 metastasis of squamous cell carcinoma, 2 metastatic and colliquated intraparotid lymph nodes) were diagnosed as malignant on MRI and USWE. Malignant lesions showed a 57 kPa average stiffness value and an inhomogeneously hypoechoic appearance on US. MRI diagnosis was histopathologically confirmed in 25/26 lesions. In 1/26 the lesion, suspected to be a pleomorphic adenoma, was instead a solitary fibrous tumor at histology.
**Conclusion:** Both MRI and USWE proved useful to differentiate benign from malignant lesions. Benign lesions showed medium-low stiffness values on USWE, while malignant lesions showed medium-high stiffness values. Due to the limited number of patients, further study is needed to assess the role of USWE in differential diagnosis between benign and malignant parotid lesions.

### Sinonasal CT phenotype in individuals with Marfan syndrome, Achondroplasia and patients with rhinosinusitis

*Authors: M. Langeland Blichfeldt, T. Sakinis, H.B. Eggesbø; Oslo/NO*

**Purpose/Objectives:** Currently, there is lack of research investigating the volumetric characteristics of the four paired sinuses and nasal cavity in individuals with Marfan syndrome and Achondroplasia, with scant attention paid to sinus anatomical variations.
This study aimed to address this gap by employing CT imaging to segment the sinuses and nasal cavity and train an artificial intelligence method for volume and surface area calculation for each paired sinuses and nasal cavity. Moreover, the study aimed to identify anatomical variations relevant to potential complications in endoscopic sinus surgery.
**Methods and materials:** The study involved 20 individuals with Marfan syndrome and 44 with Achondroplasia, and 20 patients referred for rhinosinusitis. CT scans were conducted with axial volume acquisitions and 1 mm axial slices were anonymized and extracted from the hospital's image data system. These slices were segmented using Medseg software on a dedicated Linux machine. Following 18 training iterations, final segmentation was manually corrected and transferred to 3D Slicer software for volume and surface area extraction.
Ten different sinus variations were recorded as either present or absent.
**Result:** The total sinus volume ranged as follows (median, SD): Marfan syndrome group 44 - 110 ml (80 ml, 18.3), Achondroplasia group 48 - 152 ml (76 ml, 12.1), and rhinosinusitis group 31 – 106 ml (59 ml, 20.8).
Across all three groups, the maxillary sinus consistently exhibited the largest volume and least variation, whereas the frontal sinus displayed the most variability
The nasal cavity volume ranged as follows (median, SD): Marfan syndrome group 17 - 44 ml (19 ml, 3.7), Achondroplasia group 11 - 36 ml (25 ml, 4.4), and rhinosinusitis group 12 - 34 ml (20 ml, 7.0).
The most prevalent variation with potential implications for sinus surgery was supraorbital ethmoid cells, often associated with the free anterior ethmoidal artery (Marfan and Achondroplasia group, 70-77 %).
**Conclusion:** Individuals with Marfan syndrome and Achondroplasia typically present with enlarged sinus volumes and notable volume variations, particularly evident in the frontal sinus. Additionally, they exhibit anatomical variations essential to delineate before sinus surgery.

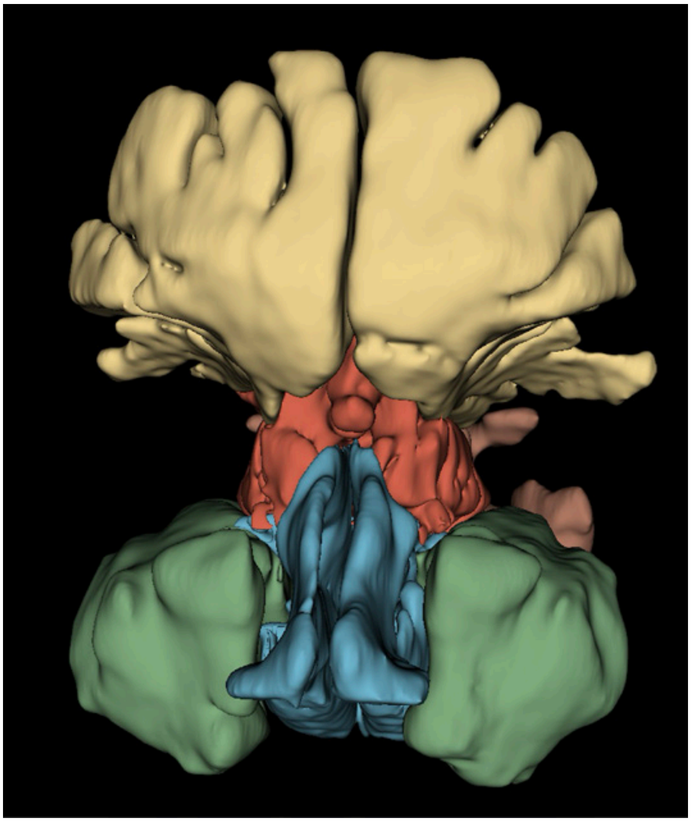

Person with Achondroplasia with frontal sinus hyperplasia (yellow)

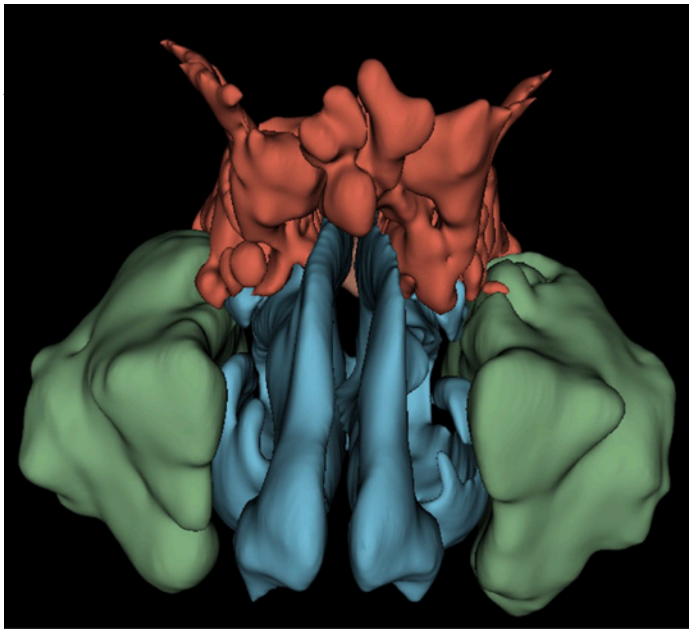

Person with Achondroplasia with frontal sinus aplasia

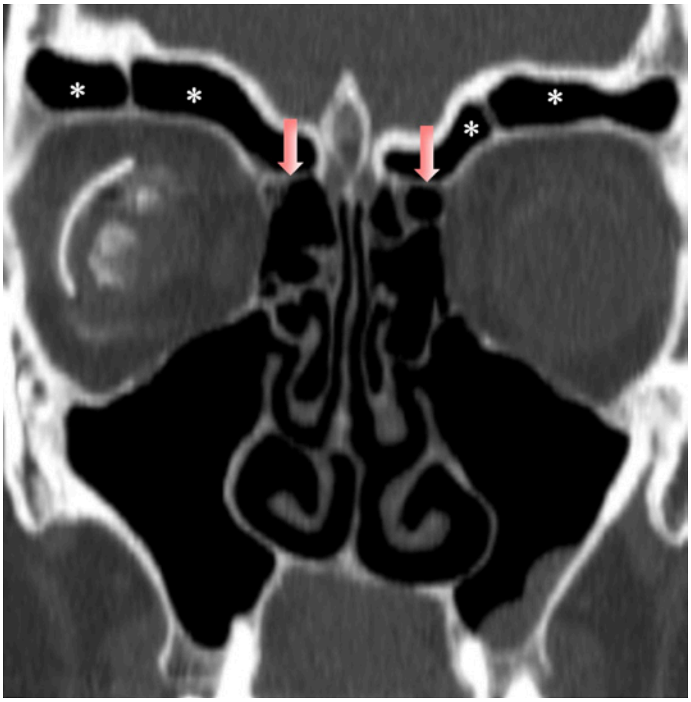

The most prevalent variation with potential implications for sinus surgery was supraorbital ethmoid cells (*), associated with free anterior ethmoidal artery (arrows)

### Approach to Videofluoroscopic Swallow Study (VFSS) interpretation in evaluating dysphagia

*Authors:* *F. A.-Z.b. Haji Johan, H.L. Soh, J. Janwar, S.S. Chok, J. Goh; Singapore/SG*

**Learning Objectives:** This poster aims to highlight the role of the videofluoroscopy swallow study (VFSS) in the evaluation of patients with dysphagia. Through the use of annotated video case studies, one can appreciate the different phases of normal swallow physiology, and recognise the common functional and anatomic abnormalities of swallowing. In addition, we aim to illustrate how combinations of various swallowing pathophysiology can result in penetration and aspiration.
**Background:** Dysphagia can severely reduce quality of life. Without appropriate and early intervention, it can lead to nutritional deficiencies, sarcopenia, aspiration pneumonia, and even death. The prevalence of dysphagia increases with age and can be associated with various medical conditions and their treatment complications.
**Imaging Findings and Procedure Details:** The current gold standard investigation for diagnosing dysphagia is the Videofluoroscopic Swallow Study (VFSS), a dynamic fluoroscopic study often performed in collaboration between speech therapists and radiologists. The use of fluoroscopy enables visualisation of the bolus flow through the digestive tract, allowing real-time evaluation of swallowing physiology and detection of any underlying functional and/or structural abnormalities. Speech therapists can trial different bolus consistencies and volumes, and observe the immediate effects. Following this, various compensatory strategies can be applied and observed.
**Conclusion:** VFSS plays a vital role in individualising dysphagia management approach, depending on the identified pathophysiology with the aim of improving patient outcomes as well as enhancing their quality of life.

*Disclaimer: This poster was presented at ECR 2024 as an Educational poster and was awarded Magna Cum Laude.*

### The impact of 3D real-IR delayed post gadolinium MRI parameterisation on the diagnostic performance and optimal descriptor selection in Ménière's Disease

*Authors: S. Connor, I. Pai, P. Touska, J. Hajnal; London/GB*

**Purpose/Objectives:** The highest performing MRI descriptors of Ménière's Disease (MD) have been shown to be saccular abnormalities and asymmetric perilymphatic enhancement in recent cross-sectional studies however these studies applied IR sequences with a time to inversion of endolymph designed to lie at the zero-crossing point, so depicting endolymphatic signal similar to bone. The impact of using MRI sequences which differentiate negative signal endolymph on the optimal choice of MRI descriptors remains unknown.
The primary aim of this study was to compare the reliability, performance and optimal combination of MRI descriptors used for the diagnosis of MD between a real-IR sequence with "zero-point" endolymph (ZPE), and an optimised real-IR sequence with negative signal endolymph (NSE).
**Methods and materials:** This retrospective single-centre cross-sectional study evaluated delayed post-gadolinium ZPE and NSE real-IR MRI in consecutive patients with Ménièriform symptoms (8/2020-10/2023). Two observers assessed 14 MRI descriptors. "Definite MD" (2015 criteria) and "all MD" ears (wider clinical criteria) were compared to control ears. Perilymphatic SNR and SIR, as well as perilymph: endolymph CNR was calculated. Cohen's kappa and risk-ratios (RR) were evaluated for each descriptor. Forward stepwise logistic regression established which combination of descriptors best predicted MD.
**Result:** The study included 132 patients (57 men; mean age 57.7 ± 13.6), with 87 "all MD" (56 "definite") and 39 control ears. The NSE sequence demonstrated increased perilymph SNR, and improved both diagnostic performance and reliability for 9/14 descriptors. However, ZPE demonstrated superior diagnostic performance for the best descriptor of "saccule absent, large as or confluent with the utricle" (RR 6.571, ZPE; 6.300, NSE) and that of "asymmetric perilymphatic enhancement" (RR 3.628, ZPE; 2.903, NSE). Both sequences combined these two descriptors in the optimal predictive model for "definite MD", with "grade 2 cochlear hydrops" also significant for NSE. ZPE and NPE descriptor combinations both correctly classified 95.8% ears. The ZPE descriptor combination performed better for "all MD" (ZPE, AUC-ROC 0.914; NSE, AUC-ROC 0.893).
**Conclusion:** Modifications to the parameterization of post gadolinium real-IR sequences increases the perilymphatic signal and enables the depiction of negative signal endolymphatic hydrops in MD. This sequence optimisation influences the optimal combination of MRI features to diagnose MD but does not improve on its overall diagnostic performance. Whilst differing parameterisation of real-IR sequences may have little impact on its diagnostic ability, radiologists should be aware that it will influence the key MRI features to analyse.

### Magnetic resonance imaging evaluation of cochlear and vestibular nerve calibre: A case-control study in Ménière's disease and endolymphatic hydrops

*Authors: R. Khalifa[1,2], P. Touska[2], I. Pai[2], F. Padormo[2], V. Goh[2], J. Hajnal[2], S. Connor[2]; [1]Cairo/EG, [2]London/GB*

**Purpose/Objectives:** To compare the calibre of the cochlear (CN), superior vestibular (SVN) and inferior vestibular (IVN) nerves on magnetic resonance imaging (MRI), both between Ménière's Disease (MD) ears and clinical controls, and between inner ears with and without endolymphatic hydrops (EH) on MRI.
**Methods and materials:** A retrospective case-control study evaluated patients undergoing MRI for suspected hydropic ear disease from 9/2017 to 8/2022. The CN, SVN, IVN and facial nerve (FN) diameters and cross-sectional areas (CSA) were measured on T2-weighted sequences whilst EH was evaluated on delayed post-gadolinium MRI. Absolute nerve calibre (and that relative to the FN) in unilateral definite MD ears (2015 Barany criteria) was compared to that in both asymptomatic contralateral ears and clinical control ears. Nerve calibre in ears with severe cochlear and vestibular EH was compared to ears without EH. Depending on whether data was normally distributed, t-tests or Wilcoxon signed-rank test/Mann-Whitney U test were applied ($p<0.001$).
**Result:** 173 patients (mean age 51.3 ±15.1, 65 men) with 84 MD (62 unilateral) and 62 clinical control ears were studied. Absolute and relative CN dimensions were decreased in both MD ears (CSA and diameter) and the contralateral asymptomatic ears (CSA) when compared to clinical controls ($p<0.001$). Absolute nerve dimensions were reduced in both severe vestibular EH (CN, IVN and SVN) and severe cochlear EH (CN) ($p<0.001$), however this was not evident when adjusted according to facial nerve calibre.
**Conclusion:** There is decreased absolute CN calibre in both symptomatic and asymptomatic MD ears as well as ears with severe cochlear and vestibular EH on MRI.

### Accuracy of cone-beam computed tomography and dental magnetic resonance imaging for detecting peri-implant bone defects at single titanium implants – An in-vitro study

*Authors: K. Hartung[1], N. Miotk[1], D. Wessling[1], F.S. Schwindling[1], M. Nittka[2], A. Özen[3], U. Ludwig[3], J.-B. Hövener[4], M. Bock[3], T. Flügge[5], A.-K. Eisenbeiss[4], P. Rammelsberg[1], S. Heiland[1], M. Bendszus[1], T. Hilgenfeld[1]; [1]Heidelberg/DE, [2]Erlangen/DE, [3]Freiburg/DE, [4]Kiel/DE, [5]Berlin/DE*

**Purpose/Objectives:** To evaluate the reliability and diagnostic value of dental magnetic resonance imaging (dMRI) and cone-beam computed tomography (CBCT) for detecting and classifying peri-implant bone defects at single titanium implants.
**Methods and materials:** Forty-eight titanium implants were inserted in bovine ribs. While twenty-four implants had surrounding standardized defects (1-wall, 2-wall, 3-wall, 4-wall) in two sizes (one mm or three mm) the other samples showed no peri-implant bone defects. CBCT and dMRI were performed and analyzed twice by two readers after randomization. MRI examinations were performed at 3T using a novel intraoral coil prototype in combination with a novel susceptibility artifact reduction sequence (compressed sensing SEMAC). Cohen's- weighted Kappa (κ), sensitivity and specificity were assessed for presence or absence of bone defects, defect size and defect type.
**Result:** An excellent intra- and inter-reader reliability (κ: 1) was calculated for bone defect detection for both imaging modalities. High sensitivity and specificity values (CBCT: 1 / 1; dMRI: 0.98 / 1) for bone defect detection were obtained for CBCT as well as dMRI. For defect type classification both CBCT (intra-reader (κ range: 0.916-0.971) and inter-reader (κ: 0.971) and MRI (intra-reader (κ range: 0.906-0.979) and inter-reader (κ: 0.888)) showed almost perfect reliabilities. The sensitivity was higher in large, three mm bone defects (CBCT: 0.92; dMRI: 0.89) compared to small, one mm bone defects (CBCT: 0.81; dMRI: 0.77). For overall defect type classification the sensitivity and specificity values were 0.86/0.99 for CBCT and 0.83/0.99 for dMRI. The lowest sensitivity was observed in small 1-wall defects in both CBCT and dMRI (0.75 vs 0.50).
**Conclusion:** The findings indicate that CBCT and dental MRI may be comparable in reliability and accuracy in identifying and classifying peri-implant bone defects around single titanium implants. Both show a high diagnostic accuracy for correct defect detection as well as defect type classification. Thus, dental MRI may be a promising imaging method in the future without the use of radiation.

### Does cochlear aperture development continue with age?

*Authors: E. Sanverdi, N. Nakhla; London/GB*

**Purpose/Objectives:** To investigate the cochlear aperture (CA) development via width by age.
**Methods and materials:** Temporal bone computed tomography (CT) images of 114 child and 127 adults (M/F: 136/105), a total of 482 temporal bones, were retrospectively analyzed. All CT studies of the temporal bone were performed on the same 16-channel multidetector CT scanner. The images were obtained with 0.5-mm collimation, 0.5-thickness, 100mA, and 120 Kv (peak). Axial-oblique reformatted images revealing the widest distance of the CA were obtained for the measurements. Images with extensive motion artifacts and patients with inner ear abnormalities (n=74 temporal bones) were not included in the study. Two radiologists, the one experienced in head and neck imaging, measured the width of CA retrospectively and blindly. For the comparison of the measurements in the different groups, and the relation with age and gender were analyzed with a dedicated statistical programme.
**Result:** Mean age was calculated 5.4 years in the children and 44.5 years in the adult group (with a range of 6 months-81 years). Mean width of CA was measured 1.71 mm in children and 1.81 mm in the adult group without a statistical significance (P=2). No side difference was found in the temporal bones of the same objects (P=1). Measurements revealed high interobserver agreement (r=0.86, P=0. 2).
**Conclusion:** CA width did not differ between children and adult groups in our study. This statement might reflect that CA is an anatomical structure completes its own development mainly in-utero term like many other inner ear structures. Further studies with larger populations and correlation of histological specimens are needed.

### Reliability and accuracy of dental MRI for in vivo identification of Cemento-Enamel-Junction

*Authors:* *M. Kranz, M. Sohani, A. Juerchott, S. Heiland, M. Bendszus, T. Hilgenfeld; Heidelberg/DE*

**Purpose/Objectives:** To evaluate the reliability and accuracy of high-resolution dental MRI (dMRI) for in vivo identification of cemento-enamel junction (CEJ) using cone-beam computed tomography (CBCT) as reference imaging modality.

**Methods and materials:** Three-Tesla dMRI and CBCT were prospectively acquired in 23 participants (mean age: 52,9 ± 10,6 years). A fat-saturated T2 weighted sequence with isotropic resolution of 0,6 mm was used in dMRI. In 319 teeth longitudinal axis for each root (389 roots) were reconstructed in CBCT and dMRI in the mesio-distal (M-D) and vestibulo-oral (V-O) direction and anonymized. Next, distance from CEJ to apex was measured altogether three times by a blinded dentist and a radiologist. Reliability/accuracy were assessed using intraclass correlation coefficients (ICCs)/Bland–Altman analysis.

**Result:** The reliability of dMRI was excellent and comparable to CBCT (intra-/inter-rater; dMRI vs. CBCT) M-D: 0,963/ 0,914 vs. 0,988/ 0,982; V-O: 0,969/ 0,868 vs. 0,99/ 0,984). Accuracy analysis revealed a mean error (95% confidence interval) of dMRI of 0,019 (-0,178/ 0,215) / 0,0005 (-0,169/ 0,170) cm in the M-D / V-O direction.

**Conclusion:** The reliability of dMRI for CEJ identification in vivo is comparable to CBCT. Mean accuracy of dMRI was high and slightly better in the V-O than in the M-D direction. Due to no distinction in signal between enamel and dentin in standard MRI sequences, however, a 95% confidence intervall up to 0.2 cm limits the accuracy of dMRI for CEJ identification in vivo.

### Diagnostic Performance of Multishot EPI (RESOLVE) and Non-EPI (HASTE) Diffusion-Weighted Imaging in Cholesteatoma and the Contribution of Signal Intensity Ratio Measurement

*Authors:* *A. Bozer, Z. Hilal Adıbelli, Y. Yener; Izmir/TR*

**Purpose/Objectives:** This study aimed to assess the diagnostic performance of Multishot Echo-Planar Imaging (EPI)(RESOLVE) and Non-EPI (HASTE) Diffusion-Weighted Imaging (DWI) sequences in cholesteatoma detection. Additionally, the study aimed to investigate the contribution of signal intensity (SI) ratio measurement in overcoming diagnostic challenges.

**Methods and materials:** The study assessed RESOLVE EPI (RS-EPI) and non-EPI DWI images of 154 patients who underwent microscopic middle ear surgery with pathological results. Two radiologists (Reader A and B) blindly evaluated the images, examining lesion location, T1-weighted (T1W) signal characteristics, and contrast enhancement on temporal bone MRI. The analysis included hyperintensity in both diffusion sequences, lesion size, SI, SI ratio, and susceptibility artifact scores.

**Result:** In our study, 62.3% (96/154) of patients were diagnosed with cholesteatoma (CHO), while 37.7% (58/154) had non-cholesteatoma (N-CHO). Reader A demonstrated 89.6% sensitivity, 79.3% specificity, 87.8% positive predictive value (PPV), and 82.1% negative predictive value (NPV) for cholesteatoma detection in RS-EPI DWI. Comparable results were found in non-EPI DWI, with 89.6%, 86.2%, 91.5%, and 83.3%, respectively. Reader B reported sensitivities, specificities, PPV, and NPV of 82.3%, 84.5%, 89.8%, and 74.2% for RS-EPI DWI, and 86.5%, 89.7%, 93.3%, and 80% for non-EPI DWI. High interobserver agreement was noted (RS-EPI: κ 0.84, non-EPI: κ 0.91). SI ratio measurements in non-EPI DWI consistently exceeded RS-EPI DWI (Reader A: 2.51, Reader B: 2.46) in the CHO group. In non-EPI DWI, the SI ratio cut-off (>1.98) effectively distinguished hyperintense lesions between CHO and N-CHO, with 82.9% sensitivity, 100% specificity, AUC: 0.901 (95% CI: 0.815-0.956), p<0.001. In RS-EPI, susceptibility artifact scores were 1.18 ± 0.7 (Reader A) and 1.04 ± 0.41 (Reader B), while non-EPI DWI had a mean score of 0.

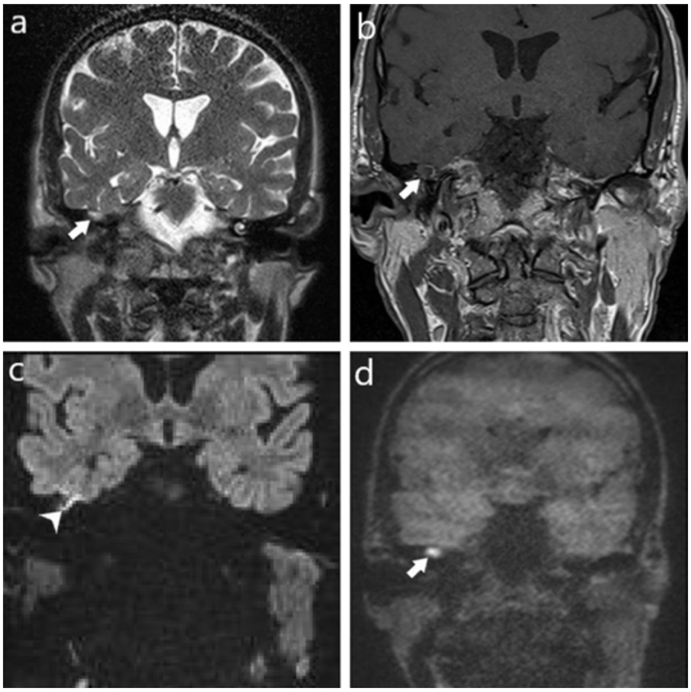

A False Positive Case in Non-EPI DWI Confirmed Histopathologically and Intraoperatively as Non-Cholesteatoma. a) T2W b) Post-contrast T1W c) RS-EPI DWI d) Non-EPI DWI

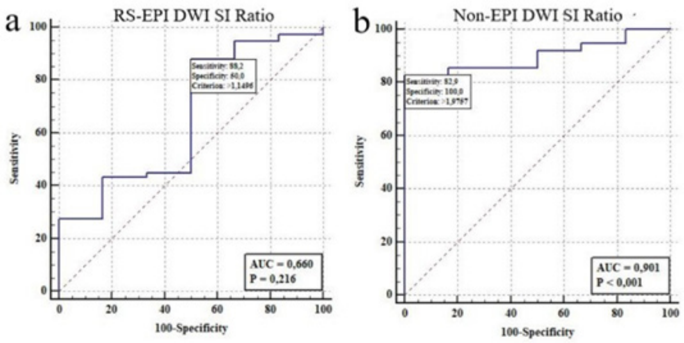

ROC Analysis of Signal Intensity Ratio Cut-off in Cholesteatoma Diagnosis using RS-EPI DWI (a) and Non-EPI DWI (b)

**Conclusion:** RS-EPI and Non-EPI DWI showed high diagnostic accuracy for cholesteatoma detection. While RS-EPI DWI isn't a substitute for non-EPI DWI, combining both enhances sensitivity. SI ratio measurement, especially in non-EPI DWI, proved valuable in challenging cases.

**Keywords:** Cholesteatoma, Diffusion-Weighted Imaging, Non-EPI, RESOLVE, Signal Intensity Ratio.

### Photon Counting CT of the temporal bone – Revealing hidden details with a fraction of the radiation dose

*Authors: D. Pellby[1], M. Vestin-Fredriksson[2], R. Siemund[1], M. Nilsson[1], L. Kull[3], A. Rönnblom[2], K. Tano[2], M.-L. Aurumskjöld[1]; [1]Lund/SE, [2]Umeå/SE, [3]Luleå/SE*

**Purpose/Objectives:** To investigate if the diagnostic quality in temporal bone imaging is maintained even at significantly lower radiation doses using Photon Counting CT (PCCT) compared to Energy-Integrating CT (EICT).
**Methods and materials:** An anthropometric phantom was constructed with a human skull enclosed by Polyurethane (PUR) and a scanned nylon coat.

Anthropomorphic phantom

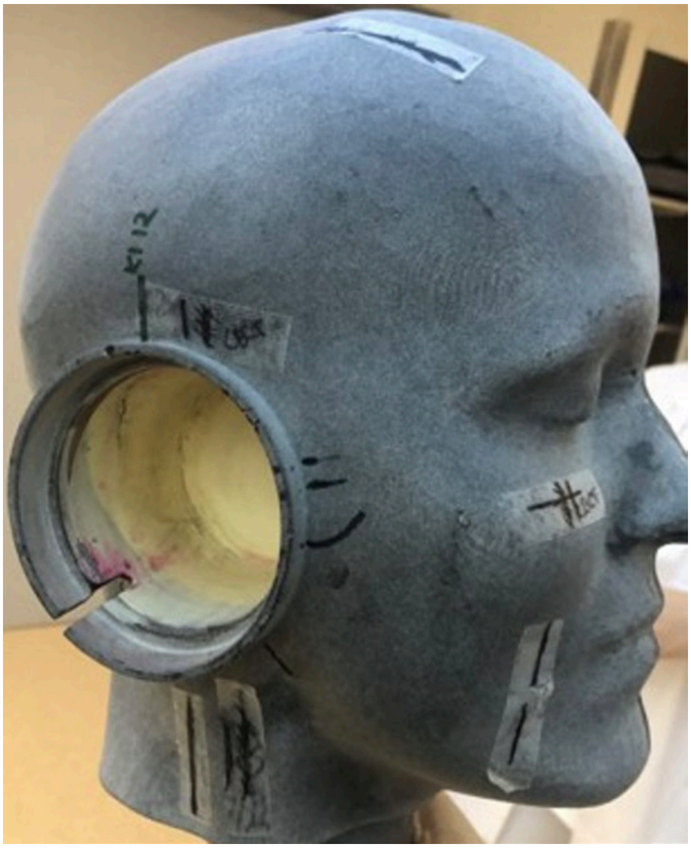

Natural conditions have been recreated by using tissue equivalent materials (soft- and hard tissue), in order to imitate real conditions regarding attenuation, spectral distribution, artefacts etc. A submersion has been drilled in the temporal bone area of the phantom making it possible to place temporal bone specimens in the anatomically correct position. A fresh frozen human temporal bone specimen was placed in the phantom and underwent temporal bone CT examinations on a PCCT system (Naeotom Alpha, Siemens) and a EICT system (Somatom Flash, Siemens) with clinical protocol settings, with CT Dose Index (CTDI) 37,5mGy for the EICT scanner and similar settings for the PCCT (CTDI 39,0mGy).

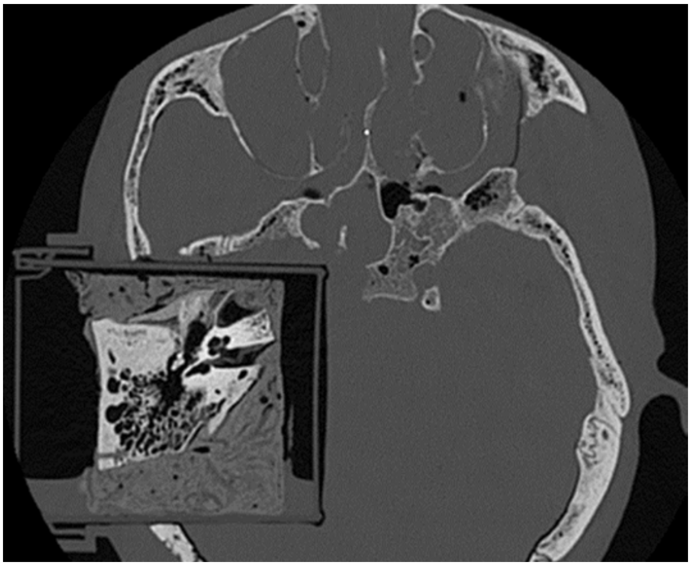

CT scan of anthropomorphic phantom with temporal bone specimen

Additional scans of the temporal bone were conducted in a dose reduction step wise approach with PCCT to examine if PCCT could perform acceptable image quality even at significantly lower dose levels. The steps conducted was at CTDI 29,1mGy, 12,4mGy, 5,1mGy, and 1,3mGy.
Ten important anatomical landmarks were chosen (incudomalleolar joint, incudostapedial joint, stapes, posterior canaliculus of the chorda tympani, modiolus, spiral lamina of the cochlea, fissula ante fenestram, vestibular aqueduct, lateral semicircular canal, tympanic segment of the facial nerve canal) to be assessed and furthermore the overall image quality.

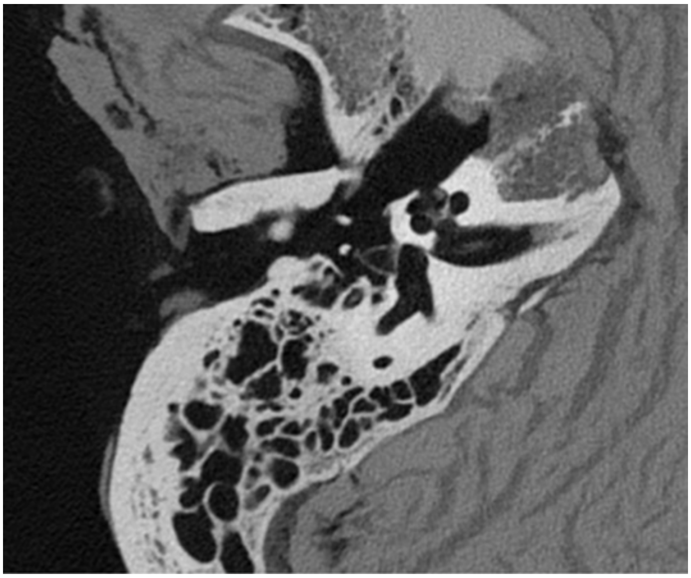

Zoom reconstruction of scanned temporal bone specimen at the level of stapes

Three blinded readers reviewed and graded all scans using a 4-point scale (poor, suboptimal, good, excellent). One reader reviewed all scans a second time for intra-rater agreement.
For the analysis of inter-rater agreement and intra-rater agreement kappa was used. Wilcoxon test was used for analysis of pairwise comparisons.
**Result:** PCCT provided excellent to good image quality for all anatomical landmarks at CTDI 39,0mGy, 29,1mGy and 12,4mGy. At CTDI 5,1mGy most landmarks provided good image quality but eg the ossicular chain landmarks provided suboptimal image quality. At CTDI 1,3mGy all landmarks provided poor image quality. PCCT scans at CTDI 39,0mGy, 29,1mGy, 12,4mGy, 5,1mGy were significantly superior to the EICT scan ($p < .05$).
**Conclusion:** PCCT was superior to EICT scan regarding image quality even at a radiation dose below 15 % of the clinical EICT-protocol. The clinical relevance / Application for this study states that maintaining excellent image quality in temporal bone diagnostics at a fraction of the radiation dose allows great benefits for all patients especially in pediatrics.

### Dedicated high-resolution MR imaging in endolymphatic hydrops: Attempting to explore its value and clinical correlations

*Authors: E. Ventura[1], R. Spasiano[1], F. Pedretti[2], D. Distefano[1], F. Piccoli[1], M. Trimarchi[1], A. Cianfoni[1]; [1]Lugano/CH, [2]Pavia/IT*

**Purpose/Objectives:** Endolymphatic hydrops (ELH) refers to an abnormal accumulation of endolymph within the inner ear membranous labyrinth. It represents the hallmark pathological feature of Menière's disease (MD), and it has traditionally been considered definitively confirmable only with postmortem examination. Nowadays dedicated high-resolution Magnetic Resonance (MR) protocols enamble to evaluate ELH-related changes in the membranous labyrinth. We then aim to evaluate the MR findings and their potential correlations with clinical aspects in patients suffering from conditions suspected of being attributable to hydrops.
**Methods and materials:** From December 2018 to February 2024, we studied a group of 70 adult patients referred to our ENT consultation with clinical scenarios suggestive of an underlying ELH. Most of them (41) had an anticipated clinical diagnosis of MD, including 22 with definite and 19 with probable MD according to 2015 diagnostic criteria. Other diagnoses mainly included fluctuating/sudden sensorineural hearing loss in mid-to-low frequencies. Every patient then underwent a dedicated 3.0 Tesla MR study protocol 4 hours after iv administration of a double dose of gadolinium to assess perylimphatic enhancement changes in the labyrinth. MR findings were evaluated according to the 2019 Bernaerts et al. grading system by three independent Neuroradiologists blinded to clinical data.
**Results:** Overall, ELH-related changes at MR were found in 28 out of 70 patients (40%). In 23 out of 41 cases (56%) with anticipated diagnosis of MD the MR findings agreed with the clinical ones. 20 out of 22 patients (90.9%) with definite MD and 3 out 19 (15.7%) with probable MD had MR findings of ELH. In only 4 out of 15 (26.6%) patients affected by fluctuating/sudden hearing loss MR confirmed the clinical suspicion of cochlear compartment hydrops. In addition, the extent of hearing for patients with ELH-positive MR findings seemed not to correlate with ELH radiological grading system.
**Conclusion:** ELH plays a central role in Meniere's disease and seems to strongly correlate with its definite form. Dedicated high-resolution MR has emerged as a reliable technique to demonstrate in vivo ELH-related changes of the inner ear membranous structure with promising application in diagnostic and prognostic purposes. The low rate of MR findings of hydrops in patients suffering from probable MD could suggest the clinician to reconsider the clinical diagnosis or the researcher to examine further pathogenetic hypotheses. Even if not specific for MD, a hydropic substrate seems to be seldomly confirmed by MR in suspected forms of isolated hearing loss. Lack of correlation between hearing impairment and MR ELH-grading may be due to the inherently fluctuating nature of hydropic hearing damage.

### 3D FSE T2 Cube Hypercube sequence versus 3D FIESTA-C sequence in evaluation of inner ear and IAC-nerve complex on 3T scanner

*Authors: M.S. Swarup, P. Gupta, M. Jain; New Delhi/IN*

**Purpose/Objectives:** Currently 3D volume acquired FIESTA-C/CISS is the most commonly used and widely accepted sequence of choice for visualizing cranial nerves at skull base and evaluating inner ear and internal auditory canal (IAC) complexes. Being a GRE sequence, FIESTA-C suffers from motion artifacts, flow artifacts, susceptibility artifacts at air-bone interfaces. We tried to compare 3D FSE T2W Cube sequence with hypercube acceleration technique (to reduce imaging time) and 3D FIESTA-C for evaluation of inner ear and IAC nerve complex anatomy and pathology.
**Methods and materials:** 35 patients with 70 inner ear-IAC were evaluated on a 3T MRI scanner (GE discovery MR750W 3T). Routine departmental protocol was followed for image acquisition including 3D axial FIESTA-C images, followed by oblique sagittal images along respective IAC and 3D axial T2 Cube Hypercube sequence from bilateral temporal bones. The patients included in the study were referred for MR imaging for evaluation of sensory-neural hearing loss. Image quality for each of these sequences and processed multi-planar reformatted images were assessed for evaluation of anatomy and pathology using following categories on a 5-point Likert scale for A) Image distortions, B) Artifacts, and C) Overall image resolution and contrast. Both the images were evaluated independently by two radiologists with more than 5 years of experience. Particular attention was given to the visibility of the anatomic structures, including four parts of the cranial nerves within the IAC (facial nerve, cochlear nerve, superior and inferior vestibular nerves), anatomic structures of the cochlea, vestibule, and three semicircular canals. The Wilcoxon signed-rank test was used to compare the data of the two techniques.
**Result:** The image quality of T2 Cube Hypercube and its reformats was found to be significantly better compared to FIESTA-C images and its reconstructed images with good interobserver agreement for rating of overall image quality. FSE T2 cube technique reduced uncertainty caused by motion artefact from FIESTA-C and improved overall image quality (resolution and contrast) compared to FIESTA-C. Both sequences have comparable imaging times (T2 cube- 3 mins 42 secs and 3D FIESTA-C- 3 mins 21 secs).

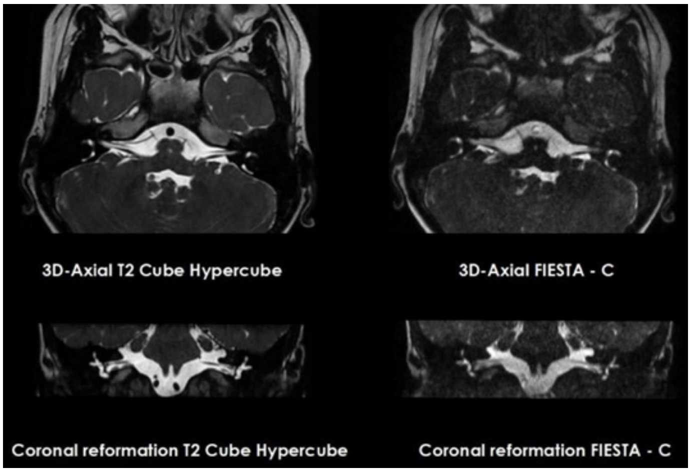

Comparison of images quality obtained with 3D FSE T2 Cube Hypercube and 3D FIESTA-C sequences

**Conclusion:** 3D FSE T2 Cube Hypercube sequence is a more appropriate and better alternative for evaluation of fine details of inner ear - IAC anatomy and pathology compared to 3D FIESTA- C sequence due to higher image quality and less susceptibility artefacts with similar aquisition times. This also obviates need of acquiring separate oblique sagittal images of IAC. We also find this sequence useful for evaluation of cisternal segments of other cranial nerves at the skull base such as imaging evaluation of trigeminal neuralgia.

### Spatial accuracy of dental MRI using a surface coil and a novel intraoral coil for tooth surfaces – Comparison with CBCT with µCT as the reference modality

*Authors: A. Skenderi[1], M. Jost[1], F.S. Schwindling[1], M. Nittka[2], A. Özen[3], U. Ludwig[3], J.-B. Hövener[4], J. Mente[1], H. Gehrig[5], M. Bock[3], T. Flügge[6], A.-K. Einsenbeiss[4], P. Rammelsberg[1], S. Heiland[1], M. Bendszus[1], T. Hilgenfeld[1]; [1]Heidelberg/DE, [2]Munich/DE, [3]Freiburg/DE, [4]Kiel/DE, [5]Kandel/DE, [6]Berlin/DE*

**Purpose/Objectives:** Accurate visualization of tooth anatomy is essential in dental diagnostics as well as treatment planning. To evaluate the advantage of a novel intraoral coil the spatial accuracy of tooth surfaces was evaluated ex vivo in direct comparison with a dedicated dental extraoral coil and cone-beam computed tomography (CBCT) using µ-CT as reference imaging modality.
**Methods and materials:** µCT, CBCT and 3-Tesla dental MRI were performed on 20 teeth (12 molars and 8 incisors). MRI measurements were performed using the MSVAT-SPACE sequence with a slice thickness / acquisition time of 440 µm/6:29 min for the extraoral coil and 180 µm / 7:09 min for the intraoral coil. The isotropic resolution of the CBCT / µ-CT were 80 / 9 µm. Tooth surfaces were reconstructed semi-automatically and registered to the µ-CT. After registration the distance between each CBCT/MRI surface and the µCT surface were calculated (in root mean square (RMS)). Surface errors were compared using the one-way analysis of variance (ANOVA).
**Result:** No substantial difference between the two different tooth types was observed (p-value range: XXX). CBCT was significantly more accurate (RME± standard deviation (SD) for molars/incisors: 0,07±0,01 mm / 0,07±0,01 mm) than the extraoral coil dental MRI (0,212±0,009 mm / 0,220±0,010 mm; $p < 0,0001$) as well as the intraoral coil dental MRI (0,11±0,01 mm / 0,10±0,01 mm; $p < 0,05$). The intraoral coil setup was significantly more accurate than the extraoral coil setup ($p < 0,0001$).
**Conclusion:** Within the limitations of an ex vivo study, the results illustrate the advantage of dental MRI using an intraoral coil compared to a dedicated extraoral coil for depicting small dental anatomy. Results of the intraoral coil were, moreover, very close to the clinical reference imaging modality CBCT, highlighting the possibilities for novel diagnostic applications as well as the reduction of radiation dose.

### The real-world impact of vestibular schwannoma fully automated volume measures on the evaluation of size change and clinical management decisions in a multidisciplinary meeting setting

*Authors:* *S. Connor, N. Wijethilake, J. Shapey; London/GB*

**Purpose/Objectives:** Clinical management decisions for patients with Vestibular Schwannoma (VS) are made within a multidisciplinary meeting (MDM). Manual extraction of linear measurements is susceptible to variation. The improved accuracy and reliability of volumetric tumour measures compared to linear measures is well recognised but may be time intensive.
The objective was to determine if the provision of deep learning based fully automated volume measures during MDM preparation resulted in a different classification of VS size changes compared to a standard approach with linear dimensions, and whether this impacted on clinical management decisions.
**Methods and materials:** This prospective cohort study evaluated 50 randomly selected adult patients (mean age 64.6 SD 12.8; 24 male, 26 female) with unilateral sporadic VS. Two simulated MDMs were conducted 35 days apart. A standard MDM (s-MDM) radiology preparation applied maximum linear tumour dimensions whilst a later computer assisted MDM (c-MDM) preparation was conducted with the aid of a report detailing fully automated volume measurements and changes. Definite and equivocal interval increases or decreases in VS size from the index to final and penultimate to final MRI studies were recorded based on previous definitions of >2 mm linear and >20% volume changes. Interval comparisons were excluded when volume segmentation was technically impossible or unacceptable. The subsequent clinical MDM outcome was classified as discharge from follow up, surveillance, intensive surveillance or intervention. Wilcoxon signed rank tests compared the classification of VS size change and the management outcomes between the s-MDM and those at the c-MDM. Spearman rank correlation coefficient compared changes in the cube of the manual linear dimensions (% Δ linear3) and the automated volume change (% Δ vol).
**Result:** The final study cohort comprised 33 patients with 57 interval MRI comparisons. There was a significant difference in the classification of VS size change between the s-MDM and c-MDM for all interval comparisons (z=2.49, p=0.01) but there was no significant difference in the resulting management decisions between the two MDMs (z=0.30, p=0.76). There was a significant positive relationship between % Δ linear3 (mean 15.46 SD 85.62%) and the % Δ vol (median 9.90 [-90.4,110.2] %) for all intervals rs =0.76, p<0.001.
**Conclusion:** The real-world application of fully automated VS volume changes in the MDM setting demonstrated a significant difference in the classification of VS size change when compared to the current “standard of care” approach with linear dimensions, however this did not result in a significant change to subsequent clinical management decisions.

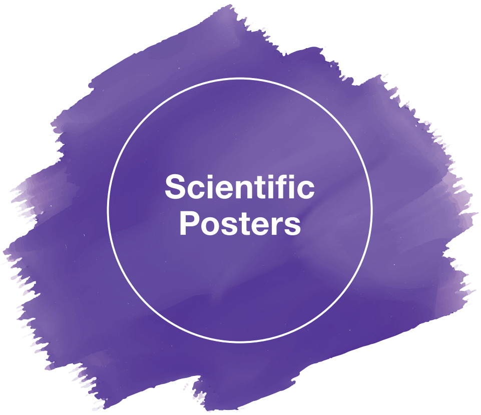

### 4D MR Parathyroids: A Review and Our Institutional Experience

*Authors:* *S.W. Goh, H.W. Khoo, A.A. Karandikar, A.Y.Q. Soon, C.K. Chew, M.Y. Lim, P.L. Kei, J.P.N. Goh; Singapore/SG*

**Purpose/Objectives:** Surgical management of primary hyperparathyroidism necessitates the precise localization of parathyroid adenomas to ensure targeted and minimally invasive intervention ([1]). Traditional imaging modalities, including Sestamibi scans, ultrasound, and computed tomography (CT), are sometimes unable to localise these adenomas. Four-dimensional magnetic resonance imaging (4D-MR) is a promising technique capitalizing on the temporal resolution of MRI, which exploits the hypervascular nature of adenomas ([2], [3], [4], [5]). Typical findings include a greater wash-in, greater degree of maximum enhancement, faster time-to-peak (TTP) and greater wash-out than both normal thyroid tissue and cervical lymph nodes. This study aims to review our institution's experience with 4D-MR as a possible alternative for adenoma localisation. A prior version of this study was presented at the Asian-Oceanian Congress of Head & Neck Radiology 2023; we now aim to present our updated data and conclusions.
**Methods and materials:** This study involves a retrospective analysis of 21 patients diagnosed with primary hyperparathyroidism who underwent 4D-MR at our institution. Unenhanced T1/T2 sequences were performed on a 3T MRI according to our department protocol, followed by Dynamic Contrast-Enhanced (DCE) analysis of selected candidate lesions. Images were interpreted by a subspecialty head and neck radiologist.
**Result:** Out of the 21 cases analysed, eleven patients subsequently underwent a parathyroidectomy, of which the adenoma was localised to the correct quadrant in nearly all the cases (10/11, 91%). Other localisation studies were either unyielding or indeterminate, reflecting the patient population that underwent 4D-MR in our clinical practice.
On the unenhanced T1 and T2 sequences, adenomas were detected in just over half of our patients (6/11, 55%), with motion artefacts (patient movement, swallowing) posing a significant challenge in identifying these small lesions. Only 3 out of 11 lesions (27%) showed (mild) restricted diffusion.
All the lesions were visible on the postcontrast sequences, possibly related to either an increased contrast ratio (given the hypervascular nature of these lesions) and less motion sensitive sequences. 9 out of 11 cases (82%) showed a classical hypervascular pattern of enhancement (akin to type A pattern on CT), while the other two showed a progressive enhancement pattern (similar to type C on CT).
**Conclusion:** The findings from our study align with the broader recognition of 4D-MR as a valuable secondary modality for localizing parathyroid adenomas, especially when traditional first-line imaging techniques prove inconclusive. The ability of 4D-MR to leverage the temporal resolution of MRI facilitates accurate localization in these challenging cases.

References:
[1] Donovan Tay, Jeeban P. Das, Randy Yeh, (2021), Preoperative localization for primary hyperparathyroidism: A clinical review, MDPI, Biomedicines, Singapore, Article 390, 9(4), https://doi.org/10.3390/biomedicines9040390, 2024-03-24, Singapore, Sengkang General Hospital
[2] K. Nael, J. Hur, A. Bauer, R. Khan, A. Sepahdari, R. Inampudi, M. Guerrero, (2015), Dynamic 4D MRI for Characterization of Parathyroid Adenomas: Multiparametric Analysis, American Journal of Neuroradiology, American Journal of Neuroradiology, New York, 2147-2152, 36(11), https://doi.org/10.3174/ajnr.A4425, 2024-03-24, United States of America, Icahn School of Medicine at Mount Sinai
[3] J.L. Becker, V. Patel, K.J. Johnson, M. Guerrero, R.R. Klein, G.F. Ranvier, R.P. Owen, P. Pawha, K. Nael, (2020), 4D–Dynamic Contrast-Enhanced MRI for Preoperative Localization in Patients with Primary Hyperparathyroidism, American Journal of Neuroradiology, American Journal of Neuroradiology, Tucson, 522-528, 41(3), https://doi.org/10.3174/ajnr.A6482, 2024-03-24, United States of America, University of Arizona
[4] Shlomo Merchavy, Judith Luckman, Michal Guindy, Yoram Segev, Avi Khafif, (2015), 4D MRI for the Localization of Parathyroid Adenoma: A Novel Method in Evolution, Wiley, Otolaryngology–Head and Neck Surgery, Tel Aviv, 446-448, Vol 154, Issue 3, https://doi.org/10.1177/0194599815618199, 2024-03-24, Israel, Assuta Medical Center
[5] Nathan A. Johnson, Mitchell E. Tublin, Jennifer B. Ogilvie, (2007), Parathyroid Imaging: Technique and Role in the Preoperative Evaluation of Primary Hyperparathyroidism, American Journal of Radiology, American Journal of Radiology, Pittsburgh, -, Vol 188, Issue 6, https://doi.org/10.2214/AJR.06.0938, 2024-03-24, United States of America, University of Pittsburgh Medical Center and School of Medicine

### A retrospective review of evaluation of BTA ultrasound guided grading of thyroid nodules with histopathological correlation and assessment of inter-rater agreement

*Authors:* *M. Krishnakumar[1], R. Bakare[1], S. Goodwin[1], S. Roberts[1], A. Mohyuddin[2]; [1]Bangor/GB, [2]Rhyl/GB*

**Purpose/Objectives:** The British Thyroid Association (BTA) Ultrasound classification is a risk stratification tool commonly used in the UK for grading thyroid nodules from U2-U5 based on their sonographic appearances. Only few studies are available on interrater variation whilst grading thyroid nodules using U scores. This study aims to evaluate interrater reliability in ultrasound assessment of thyroid nodules and to estimate the diagnostic accuracy of the BTA classification.
**Methods and materials:** A retrospective review of thyroidectomy specimens over a 12 month period in 2023 was carried out. This totalled to 56 patients. The U grade provided in the original report and the final histopathology results were recorded. Furthermore, three observers (two Sonographers and a Consultant Radiologist) were blinded to patient information and histopathology results. The observers were then asked to retrospectively allocate a U grade based on ultrasound images saved to a power point document.
**Result:** The rate of malignancy for each U category was analysed according to the score provided on the original report. U2 had a malignancy rate of 6%, U3 was 32%, U4 was 56% and U5 was 67%. The sensitivity for detection of malignant thyroid nodules based on original US reports was high at 90% and the specificity was 71%. The interrater agreement for U scores was calculated for the original report and three further raters with a Fleiss Kappa score of 0.52 indicating moderate agreement between the raters.
**Conclusion:** This study concludes that the BTA classification has a high sensitivity for detection of malignant thyroid nodules and revealed a moderate interrater agreement. We aim to repeat this study comparing the BTA U grading with the ACR/EU TIRADS score.

### Better detection of recurrent pleomorphic adenoma with the DESS sequence

*Authors: M. Malik, J. Gao, S. Morley, M. McGurk; London/GB*

**Purpose/Objectives:** Pleomorphic adenoma is the most prevalent benign salivary gland tumour, often treated with surgical resection due to the risk of malignant transformation. The post-operative recurrence rate has been reported at 3.1%, with subsequent recurrences occurring up to 40% following repeat surgery, posing a formidable management challenge. One factor contributing to recurrence is incomplete excision and we postulate that this may be partly due to underestimating the extent of recurrent disease on imaging prior to surgery. In our practice, the primary MRI sequence used to assess disease recurrence was a fat-suppressed fluid-sensitive sequence, such as STIR. While sensitive for pleomorphic adenoma recurrence, it is limited by the large slice thickness. By contrast, the Double Echo Steady State (DESS) sequence (Siemens), used in previous studies for facial neuronography, offers smaller slice thickness, which we propose improves the visualisation of small nodules and, being a volumetric sequence, enables multiplanar reconstruction. We aim to compare the DESS and STIR sequences in assessing the spread and number of nodules of recurrent pleomorphic adenoma and the feasibility of using 3D mapping to aid surgical planning.
**Methods and materials:** 7 adults with confirmed recurrent pleomorphic adenoma after surgical intervention were included. MRI scans were performed on 3T scanners, including both a DESS sequence and a fat-suppressed fluid-sensitive sequence (e.g. STIR or T2 dixon fat sat) in the same session. The acquired scans were imported to 3D Slicer, on which the number of nodules and extent of disease recurrence in three axes was measured. After segmentation, 3D reconstructions were generated.
**Result:** In the 5 patients with a quantifiable number of nodules of recurrence (the other 2 patients had >50 nodules), the DESS sequence identified on average a 65.5% increase in the number of nodules compared to STIR. In all 7 patients, the DESS sequence revealed an increase in disease extent in 13 measurements (62%), no difference in extent in 2 measurements (10%) and a decreased extent in 6 measurements (29%), compared to STIR. Additionally, we present 3D models, made using the DESS sequence, highlighting the relation of the recurrent disease to important anatomical structures.
**Conclusion:** The DESS sequence holds potential in optimising surgical management of recurrent pleomorphic adenoma by improved identification of recurrent disease nodules. Further prospective research should focus on the clinical application of DESS in parotid MRI protocols and the impact of 3D models on surgical decision-making and clinical outcomes.

### Children with Complete Aplasia of the Semicircular Canals – Outcome after CI Implantation and Correlation with Radiological Data and associated Syndroms

*Authors: A. Ahmed, K. Döring, F. Götz, H. Lanfermann, A. Warnecke, A. Giesemann; Hannover/DE*

**Purpose/Objectives:** Severe Inner Ear malformations are often treated with CI. Factors influencing the outcome are the possible nerve hypoplasia, the malformation itself but also an accompanying syndromic disease. Data on outcome is essential for realistic expectations. About 60% of children with complete aplasia of the semicircular canals have CHARGE association. Only few do not show any accompanying disability or syndrom.
**Methods and materials:** Inclusion criteria are the diagnosis of complete aplasia of the semicircular canals done by CT of the temporal bone and the treatment with Cochlea Implantation. Imaging findings on CT and MRI especially nerve hypoplasia were evaluated. Children were followed at least 2 years after implantation. Evaluation was primarily done using Categories of auditory performance (CAP) – a score from 0 to 12 describing states from being unaware of environmental sounds to the use of telephone with unfamiliar speakers.
**Result:** 42 children with complete aplasia of the semicircular canals were identified and 29 of them were implanted and had a follow up of at least 2 years available. In 23 cases MR Images were available. The average follow-up time being 5.3 years with an average age of the children was 7.5 years at last follow up. The best CAP score reached by two of those children is 9 (understanding conversation with unfamiliar speakers). Both children do not have CHARGE syndrom and no nerve hypoplasia on MRI. The best CAP score reached with CHARGE syndrom is 7 in one case. The majority showed CAP scores between 2 and 4 (15 children), mostly demonstrating a clearly visible cochlear nerve on the implanted ear.
**Conclusion:** Children with complete aplasia of the semicircular canals show auditory benefit from cochlea implantation, although varying and in the majority to a limited degree. Best results show children with no associated CHARGE syndrom.

### Cochlear size as a predictor of depth of angle insertion: An MRI validation study

*Authors: O. Casals-Farre, T. Sivakanthan, Y. Kamhieh, A. Hall, R. McLeod, A. Khatib, J. Johnston, M. Hart; Cardiff/GB*

**Purpose/Objectives:** Depth of insertion of cochlear implant (CI) electrode is an area of increasing interest with Xu et al proposing insertion depth angle as a more utile measure [1]. Cochlear size varies even between individuals with normal morphology [2] and can affect depth of insertion. Escude et al [3] identified 2 fixed cochlear measurements on high-resolution CT (HRCT) which predicted depth of insertion. With the development of the Cochlear 'SmartNav' technique, the team have identified a new composite CT view of the cohlea which allows for one single measure to predict depth of insertion [4]. However not all centres perform CT prior to CI surgery, and no data exists to date on MRI-based measurements. This project examines the validity of the measurements on MRI images.
**Methods and materials:** A retrospective review of CTs of 50 temporal bones from consecutive CI cases was carried out by two senior head and neck radiologists. The measurements were conducted first on CT and then independently on MRI, by two different raters. We assessed for validity of the measurements taken on MRI compared to those on CT, and for inter-rater reliability.
**Result:** Data on validity of MRI measurements and senior head and neck radiologist inter-rater reliability will be presented
**Conclusion:** Escude et al.'s [3] exciting work produced two simple measurements of cochlear size which could predict depth of insertion angle for CI electrodes. However this is based on pre-operative CT scans which are not routinely conducted in all centres. Our study will show whether these measurements can be reliably replicated using the routine pre-operative MRI.

### Development of a Radiomics-based Model for Differentiating Parotid Neoplasms

*Authors: S.W. Goh, A.Y.Q. Soon, A.C. Srinivasa, J.L. Tan, K. Choy, A. Shrestha, B.K. Prakash; Singapore/SG*

**Purpose/Objectives:** The differentiation of parotid tumour varieties is crucial due to the differences in their management. Pleomorphic Adenoma (PA) and Warthin tumour (WT) represent 2 of the most common parotid tumours. PA should be resected as there is a risk of carcinoma ex pleomorphic adenoma. MRI is the pivotal modality their characterization. On MRI, PA and WT classically exhibit distinct signal characteristics and enhancement patterns, with PA being T2 hyperintense and enhancing, and WT being T2 hypointense and hypoenhancing ([1], [2]), though some difficult lesions offer diagnostic challenges for the head & neck radiologist.
There is a growing arsenal of tools to characterize these, e.g. DCE analysis ([3]), though the burgeoning field of artificial intelligence has introduced novel tools for characterization ([4]). Among these, radiomics, which utilizes quantitative image features of these tumours, is a promising technique for the characterization of these lesions, in this case PA and WT.
**Methods and materials:** 63 MRI studies were analysed retrospectively, comprising 44 PA and 19 WT. MRI was performed according to departmental protocol. Image features were extracted from the T1, T2 (non-fat saturated), and T1 post-contrast sequences, the most commonly observed sequences across all studies.
Ground truth labeling was performed by an expert head & neck radiologist, with independent segmentation of the right/left parotids and tumours.
Pyradiomics ([5]) was employed for feature extraction from T1 scans, following which machine learning classifiers (Random Forest, Gradient Boosting Machine, Support Vector Machine & Logistic Regressor) were trained on the 1, 2 and 3 top image features. Training/validation had 48 and testing had 13 datasets.
**Result:** After normalization, the top five image features used for analysis were Joint entropy, High grey level run emphasis, Joint average, Short-run high grey level emphasis and Sum entropy.

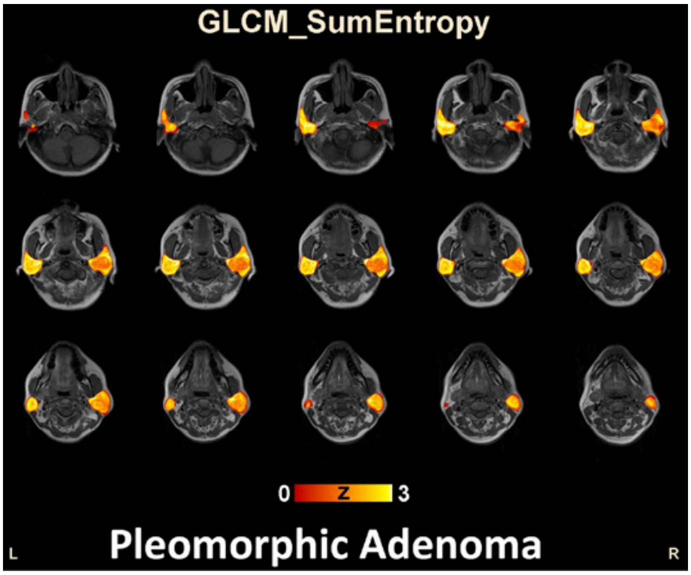

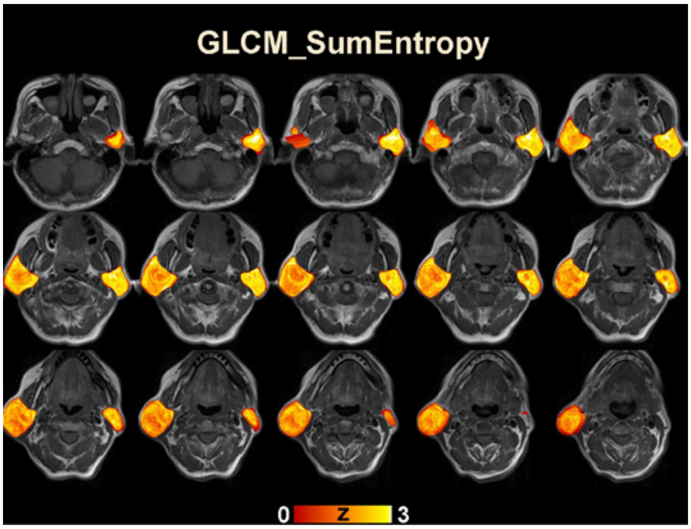

Heat Maps of the Sum Entropy Feature, One Case Each of Pleomorphic Adenoma and Warthin Tumour

For instance, WT shows a higher joint average and joint entropy feature score than PA.
The models trained on 1 feature (sum entropy) achieved a maximum accuracy of 96% and an F1 score of 0.97 (PA)/0.93 (WT), and the model trained on 2 features (sum entropy and joint entropy) reached up to a perfect accuracy (100%) and F1 score (1.0) for both PA and WT.
The models trained on 3 features (sum entropy, joint entropy and joint average) also achieved up to perfect accuracy and F1 score.

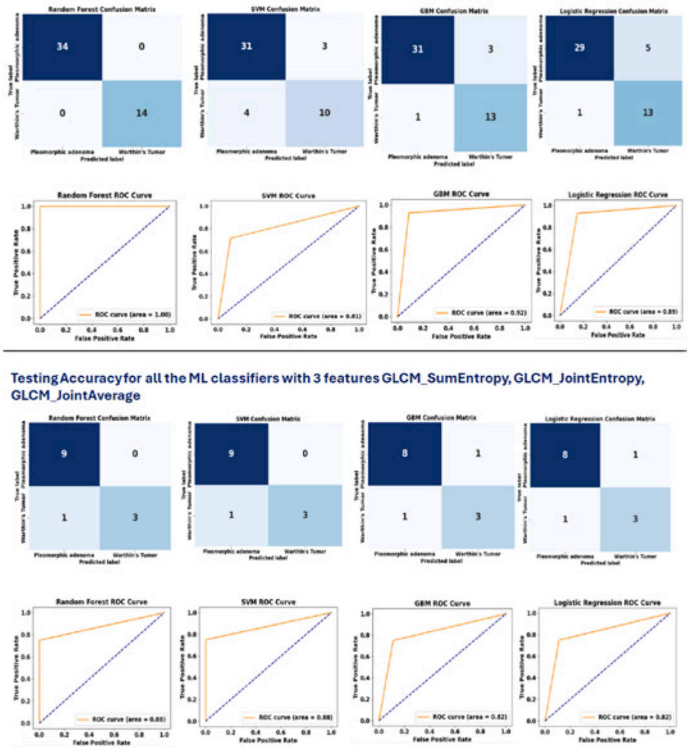

Training Statistics for the Classifier Models Trained on Three Features

**Conclusion:** In summary, the development of radiomics-based prediction models represents a promising avenue in the analysis of parotid tumours. The findings underscore the efficacy of leveraging such algorithms for the accurate diagnosis of PA and WT, highlighting a significant advancement in the characterization of parotid tumours through non-invasive imaging techniques.

References:
[1] H. Coudert, S. Mirafzal, A. Dissard, L. Boyer, P.-F. Montoriol, (2021), Multiparametric magnetic resonance imaging of parotid tumors: A systematic review, Elsevier, Diagnostic and Interventional Imaging, Clermont-Ferrand, 121-130, Vol 102, Issue 3, https://www.sciencedirect.com/science/article/pii/S2211568420302163?via%3Dihub, 2024-03-24, France, University Hospital Gabriel-Montpied
[2] Soung Yung Kim, Urs Borner, Jung-Hyun Lee, Franca Wagner, Dechen W. Tshering Vogel , (2022), Magnetic resonance imaging of parotid gland tumors: a pictorial essay, BMC, BMC Medical Imaging, Bern, -, 33, https://bmcmedimaging.biomedcentral.com/articles/10.1186/s12880-022-00924-0, 2024-03-24, Switzerland, Röntgeninstitut Marktgasse AG
[3] Mohini Kushwaha, Jyoti Kumar, Anju Garg, Ishwar Singh, Nita Khurana, (2023), Differentiation of various salivary gland tumours using diffusion-weighted MRI and dynamic contrast-enhanced MRI, Polish Journal of Radiology, Polish Journal of Radiology, Delhi, 203-215, 88(1), https://www.polradiol.com/Differentiation-of-various-salivary-gland-tumours-using-diffusion-weighted-MRI-and-dynamic-contrast-enhanced-MRI,126,50620,0,1.html, 2024-03-24, India, Maulana Azad Medical College
[4] Janita E. van Timmeren, Davide Cester, Stephanie Tanadini-Lang, Hatem Alkadhi, Bettina Baessler, (2020), Radiomics in medical imaging—"how-to" guide and critical reflection, Springer Open, Insights into Imaging, Zurich, 1-16, 11, Article 91, https://insightsimaging.springeropen.com/articles/10.1186/s13244-020-00887-2#citeas, 2024-03-24, Switzerland, University Hospital Zurich, University of Zurich
[5] Joost J M van Griethuysen, Andriy Fedorov, Chintan Parmar, Ahmed Hosny, Nicole Aucoin, Vivek Narayan, Regina G H Beets-Tan, Jean-Christophe Fillion-Robin, Steve Pieper, Hugo J W L Aerts, (2017), Computational Radiomics System to Decode the Radiographic Phenotype, AACR Journals, Cancer Research, Boston, e104–e107, 77 (21), https://doi.org/10.1158/0008-5472.CAN-17-0339, 2024-03-24, United States of America, Brigham and Women's Hospital Harvard Medical School

### MRI Imaging role of the residual/recurrent pleomorphic adenoma

*Author: S. Siurana; Barcelona/ES*

**Purpose/Objectives:**
- To review our institutional experience with imaging of recurrent pleomorphic adenoma in the last ten years.
- To establish risks of secondary malignant transformation

**Methods and materials:** Retrospective imaging and chart reviews of 19 patients (10 males, 9 females; aged between 15–69 years) with recurrent pleomorphic adenoma of the parotid gland.
MR images of treated patients were reviewed as to the available sequences and location, multiplicity, signal intensity, cellularity, and enhancement pattern
**Result:** Solitary recurrent lesion was seen in 5 patients and multiple in 14 patients.
The majority of recurrences were confined to the parotid space but in 6/19 cases they were located distant from the operative bed.
The volume of the recurrent tumor was ≤ 10 ml in 13, 10– 20 mm in 2, 20–30 mm in 1, and ≥ 30 mm in
T2 fat saturation easily identifies most of the recurrent lesions.
63% of the lesions showed high T2 signal and high ADC.
Of the 7 lesions with ADC < 1.3, malignant transformation was identified in 3.
**Conclusion:** Understanding the diversity of radiological features of recurrent pleomorphic adenoma, using conventional and functional MR images, improves the radiologist's ability to make correct diagnoses.
Given the slow growth of residual lesions and low risk of secondary malignant transformation MRI follow-up after all first recurrences, can help identify a patient's individual growth rate and can avoid a need for more extensive surgery.

### Radiological assessment of resectability in Head and Neck cancers: Our experience on CT, MRI, and PET/CT

*Authors: M.S. Swarup, P.N.S. Varalakshmi, S. Sree V S, H. Sharma, G. Sindhwani, R. Gupta Ghasi; New Delhi/IN*

**Purpose/Objectives:** Head and neck cancer surgeons often rely on imaging studies to determine the resectability of malignant neoplasms. The purpose of this presentation is to share our experience on the diagnostic accuracy of various cross-sectional imaging modalities (CT, MRI, PET/CECT) for local resectability of various head and neck cancers.
**Methods and materials:** American Joint Committee on Cancer (AJCC) Staging Manual defines T4a and T4b stages in advanced head and neck cancers as resectable and unresectable diseases respectively. Even among patients classified as T4a with advanced resectable tumours, specific factors play a crucial role in determining whether the chosen surgical approach carries a high risk of significant complications, morbidity and mortality.
The available contrast-enhanced CT, MRI, and PET/CECT studies were evaluated in 50 patients with head and neck carcinomas of various subsites, that were referred for the staging or restaging of cancer after neoadjuvant therapy. Tumours were assessed for the presence of resectability-defining factors (T4b status) and critical T4a factors, which include the following:
1) Major arterial encasement
2) Prevertebral fascia involvement
3) Mediastinal infiltration
4) Tracheal and esophageal extension
5) Laryngeal cartilage invasion
6) Pre-epiglottic fat involvement
7) Dural infiltration
8) Bone involvement (mandible/ maxilla, hyoid)
9) Skull base and pterygoid plate invasion
10) Involvement of masticator space/pterygoid muscles
11) Involvement of pterygo-palatine fossa
12) Perineural tumour spread
13) Orbital involvement
14) Brachial plexus invasion

**Result:** Contrast-enhanced CT, Contrast-enhanced MRI, and PET/CECT are reliable imaging examinations for defining local resectability of head and neck cancers with variable results in diagnosing criteria of resectability according to the above-mentioned critical factors. MR imaging shows relatively higher sensitivity but lower specificity than CECT. PET/CT is often useful in cases showing false positivity on MRI. Imaging is good in depicting most of the critical signs of unresectability except for accurate depiction of prevertebral fascia involvement and early cartilage infiltration.
Various critical issues of resectability will be illustrated with relevant cases with a multimodality approach.
**Conclusion:** Imaging plays a crucial role in assessing resectability for head and neck cancers. The decision to pursue extensive and potentially morbid tumor resections should be a joint consideration between surgeons, radiologists and patients, informed by the most accurate available imaging techniques.

### Refining The Management of Bethesda III Thyroid Nodules by Combining Nuclear Atypia with Sonographic Risk Stratification

*Authors: H.W. Khoo, H. Li, A.Y.Q. Soon, S.W. Goh, C.C.Y. Lee, M.M. Bundele, J.Y. Gan, E.W. Fu, M.Y. Lim, J.P.N. Goh; Singapore/SG*

**Purpose/Objectives:** Ultrasonography stands as the key modality in the workup of thyroid lesions ([1]). Multiple sonographic risk stratification systems (RSS) have been proposed to standardize reporting and provide some prognostication, with some of the more widely-used being the European Thyroid Imaging Reporting and Data System (EU-TIRADS) ([2]), the American College of Radiology-TIRADS (ACR-TIRADS) ([3]), and the American Thyroid Association guidelines (ATA) ([4]). Despite this, cytology, as defined by the Bethesda classification ([5]), is essential in diagnosing malignancy. Bethesda III nodules may present a diagnostic challenge due to their ambiguous nature and 10-30% risk of malignancy. Further stratification by nuclear atypia and sonographic risk could aid in the decision-making process for or against a diagnostic hemithyroidectomy. The preliminary results of this study were presented in the World Congress on Thyroid Cancer, the 5th Annual Meeting of the Society of Otolaryngology Head Neck Surgery of Singapore, and Singapore Health and Biomedical Congress, all in 2023.
**Methods and materials:** We retrospectively reviewed consecutively resected Bethesda III thyroid nodules from Tan Tock Seng Hospital between 2010 and 2016. Blinded to their diagnoses, 2 radiologists independently classified the nodules according to the aforementioned three RSS. A third radiologist assessed the images to resolve discordance between the two radiologists. A pathologist reviewed the cytology to determine the presence of nuclear atypia. Univariate and multivariate analyses were performed to determine the indicators of malignancy. The risk of malignancy (ROM) was calculated for each indicator.
**Result:** Sonographic images were matched to the histological diagnoses in 246 out of 289 nodules. 64 (26%) were malignant (35 papillary carcinomas, 26 follicular carcinomas, 2 lymphomas, 1 poorly differentiated carcinoma). Nuclear atypia and higher EU, ACR-TIRADS, or ATA sonographic risk were significantly more common in malignant nodules ($p<0.01$) than benign nodules (including NIFTP). All RSS remained significant on multivariate logistic regression. The area under the receiver-operator-curve of the composite diagnostic model incorporating age, sex, nuclear atypia, and RSS was 0.67, 0.66, 0.69, for EU-TIRADS, ACR-TIRADS, and ATA, respectively. In sonographically high risk nodules, the ROM was significantly higher in those with nuclear atypia than those without atypia ($p<0.01$).
**Conclusion:** These risk factors can aid in the management of Bethesda III nodules – a diagnostic hemithyroidectomy should be considered in sonographically high-risk nodules with nuclear atypia. In contrast, less invasive strategies like molecular testing or repeat fine needle aspiration may be beneficial in other lower-risk categories.

References:
[1] Cosimo Durante, Giorgio Grani, Livia Lamartina, Sebastiano Filetti, Susan J. Mandel, David S. Cooper, (2018), The diagnosis and management of thyroid nodules a review, JAMA Network, JAMA, Roma, 914-924, 319(9), https://doi.org/10.1001/jama.2018.0898, 2024-03-24, Italy, Università di Roma "Sapienza"
[2] Gilles Russa, Steen J. Bonnema, Murat Faik Erdogan, Cosimo Durante, Rose Ngu, Laurence Leenhardt, (2017), European Thyroid Association Guidelines for Ultrasound Malignancy Risk Stratification of Thyroid Nodules in Adults: The EU-TIRADS, European Thyroid Journal, European Thyroid Journal, Paris, 225-237, 6(5), https://doi.org/10.1159/000478927, 2024-03-24, France, Pitié Salpêtrière Hospital, Pierre and Marie Curie University
[3] Franklin N Tessler, William D Middleton, Edward G Grant, Jenny K Hoang, Lincoln L Berland, Sharlene A Teefey, John J Cronan, Michael D Beland, Terry S Desser, Mary C Frates, Lynwood W Hammers, Ulrike M Hamper, Jill E Langer, Carl C Reading, Leslie M Scoutt, A Thomas Stavros, (2017), ACR Thyroid Imaging, Reporting and Data System (TI-RADS): White Paper of the ACR TI-RADS Committee, Elsevier, Journal of the American College of Radiology, Alabama, 587-595, 14(5), https://doi.org/10.1016/j.jacr.2017.01.046, 2024-03-24, United States of America, University of Alabama at Birmingham
[4] Bryan R. Haugen, Erik K. Alexander, Keith C. Bible, Gerard M. Doherty, Susan J. Mandel, Yuri E. Nikiforov, Furio Pacini, Gregory W. Randolph, Anna M. Sawka, Martin Schlumberger, Kathryn G. Schuff, Steven I. Sherman, Julie Ann Sosa, David L. Steward, R. Michael Tuttle, Leonard Wartofsky, (2015), 2015 American Thyroid Association Management Guidelines for Adult Patients with Thyroid Nodules and Differentiated Thyroid Cancer: The American Thyroid Association Guidelines Task Force on Thyroid Nodules and Differentiated Thyroid Cancer, Mary Ann Liebert, Thyroid, Aurora, 1-133, 26(1), https://doi.org/10.1089%2Fthy.2015.0020, 2024-03-24, United States of America, University of Colorado School of Medicine
[5] Syed Z. Ali, Zubair W. Baloch, Beatrix Cochand-Priollet, Fernando C. Schmitt, Philippe Vielh, Paul A. VanderLaan, (2023), The 2023 Bethesda System for Reporting Thyroid Cytopathology, Mary Ann Liebert, Thyroid, Baltimore, 1039-1044, 33(9), https://doi.org/10.1089/thy.2023.0141, 2024-03-24, United States of America, The Johns Hopkins Medical Institutions

### Role of MRI evaluation of sarcopenia in patients with oral cancer underwent demolitive surgery and nutritional therapy

*Authors: R. Raspante, P. Buscemi, A.A. Blandino, O. Berlina, A. Monteleone, A. Lo Casto; Palermo/IT*

**Purpose/Objectives:** Patients affected by oral cancer may undergo malnutrition with adverse survival rate. Assessment of malnutrition is critical for prognosis in oral cancer patients. Sarcopenia is a marker of malnutrition and imaging techniques are used to evaluate sarcopenia. MRI was recently proposed, instead of the most used CT, to evaluate sarcopenia in oral cancer patients. Aim of this work was to evaluate sarcopenia by MRI in patients with oral cancer, underwent demolition surgery and nutritional therapy.
**Methods and materials:** 35 patients with oral cancer, who underwent surgery and received nutritional therapy, between January 2020 and May 2023, submitted to staging and follow up MRI, were retrospectively evaluated. Clinical nutritional assessment included: medical history, physical examinations, Mini Nutritional Assessment (MNA) tests, anthropometric data, Bioelectrical Impedance Analysis (BIA), grip tests, and laboratory tests. As index of sarcopenia the volumetric assessment of paravertebral muscles, performed at C3 level through segmentation of 5 axial T2 MR images, was done, using the software "3D-slicer". Sternocleidomastoid muscles were not included in the segmentation area because, in patients with oral cancer, they can be involved in demolitive surgery or invaded by metastasis.
**Result:** Kaplan-Meier survival curves showed no significant differences based on TNM stage ($P=0.74$). MNA test scores indicative of malnutrition were associated with shorter survival rate ($P=0.021$). Patients with artificial nutrition had a reduced survival rate ($p<0.001$). Volumetric assessment of paravertebral muscles with MRI revealed a reduced paravertebral muscle volume in 5 patients treated with artificial nutrition (Percutaneous Endoscopic Gastrostomy and Nasogastric tube), with a maximum reduction of 30%, and an increased muscle volume in 5 patients with natural nutrition, with a maximum increment of 42%.
**Conclusion:** Volumetric evaluation of paravertebral muscles using MRI seems a useful tool for sarcopenia diagnosis and monitoring in patients with oral cancer suffering of malnutrition.

References:
[1] Pressoir M. et al., (2010), Prevalence, risk factors and clinical implications of malnutrition in French Comprehensive Cancer Centres, British Journal of Cancer, https://www.nature.com/articles/6605578#citeas
[2] De Bree R. et al., (2022), Sarcopenia and its impact in head and neck cancer treatment, Current Opinion in Otolaryngology & Head and Neck Surgery, https://journals.lww.com/co-otolaryngology/fulltext/2022/04000/sarcopenia_and_its_impact_in_head_and_neck_cancer.3.aspx
[3] Swartz JE et al., (2016), Feasibility of using head and neck CT imaging to assess skeletal muscle mass in head and neck cancer patients, Oral Oncology, https://pubmed.ncbi.nlm.nih.gov/27865369/#:~:text=Discussion%3A%20Assessment%20of%20skeletal%20muscle,imaging%20or%20additional%20patient%20burden.
[4] S.I. Bril et al., (2021), Validation of skeletal muscle mass assessment at the level of the third cervical vertebra in patients with head and neck cancer, Oral Oncology, https://www.sciencedirect.com/science/article/pii/S1368837521007247?via%3Dihub
[5] Zwart AT et al., (2021), Skeletal muscle mass and sarcopenia can be determined with 1.5-T and 3-T neck MRI scans, in the event that no neck CT scan is performed, European Radiology, https://link.springer.com/article/10.1007/s00330-020-07440-1

### Sonographic features of thyroid nodules with indeterminate cytology – Are there differences between lesions with nuclear or architectural atypia?

*Authors:* *D. Davda, N. Sriskandan, A. Chandra, M. Munonyara, A. Rovira, R. Simo, A. Velusamy, S. Anandappa, P. Carroll, P. Touska; London/GB*

**Purpose/Objectives:** Indeterminate (atypical) cytological results following ultrasound-guided fine needle aspiration of thyroid nodules (Table 1) can now be dichotomised into those with nuclear atypia (Thy3aNu) and those without (Thy3aNo).[1] Malignancy rates are higher for Thy3aNu nodules resulting in more aggressive treatment.[2] However, sonographic differences have not yet been explored.

| System | Country | Category | Category description |
|---|---|---|---|
| Royal College Of Pathologists (Rcpath) Thy Terminology For Reporting Thyroid Fine-Needle Aspiration Cytology | U.K. | Thy3a | Cytological/nuclear or architectural atypia or other features raising the possibility of neoplasia but insufficiently to allow placement into any other category |
| Bethesda Reporting System For Thyroid Cytopathology | U.S.A. | III | Atypia of undetermined significance |

Table 1: Examples of indeterminate (atypical) thyroid cytology categories

AIMS:

1. Assess sonographic differences between nodules with Thy3a-Nu and Thy3a-No cytology with reference to the 2023 international expert consensus lexicon for thyroid nodules.[3]
2. Determine whether mixed nodule echogenicity (isoechoic with hypoechoic areas - image 1) is discriminatory.
3. Establish inter-reader agreement for sonographic features.

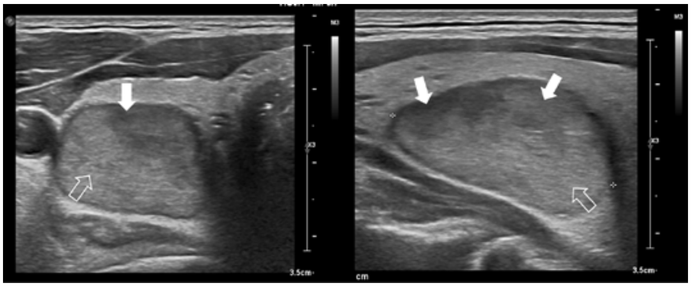

Example of a solid nodule of mixed echogenicity. Areas of intranodular hypoechogenicity are indicated by the solid arrows and the isoechoic areas by the outlined arrows.

**Methods and materials:** Nodules with Thy3a cytology from 05/2023 to 01/2024 were identified. B-mode (≥2 views) and duplex colour Doppler (where available) images were extracted. Two blinded head and neck radiologists evaluated the images in accordance with the 2023 international Thyroid US lexicon. Inter-rater agreement was assessed using Cohen's kappa. Differences between US features and type of atypia were assessed using the Pearson Chi2 test using SPSS v29 (IBM). A p-value of <0.05 was deemed significant.

**Result:** Forty nodules were identified in 39 patients; 52.5% (n=21) yielded Thy3a-No cytology and 47.5% (n=19) Thy3a-Nu cytology. Lesion characterstics and management are presented in table 2.

| | Thy3a-No | Thy3a-Nu | Comments | p-value |
|---|---|---|---|---|
| Maximum size | 2.9 cm (range: 0.5-7.4 cm) | 1.9 cm (range: 0.5-7.8cm) | Thy3aNo: non-significant trend towards larger lesions. | 0.06 |
| Conservative treatment | 81.0%, n=17 | 21.0% (n=4) with <1cm nodules were managed conservatively (active surveillance) | Thy3aNu: <1cm nodules were managed conservatively (active surveillance). | - |
| Surgical treatment | 19.0% (n=4) | 73.7%, n=14 | Thy3aNo: diagnostic lobectomy yielding benign disease in 3 cases and PTC in 1 case (operated due to nodule size). | - |
| Awaiting surgery | N/A | 5.2% (n=1) | | - |
| Overall malignancy rate | 4.8% (n=1) | 36.8% (n=7) | Approximate values (analysis includes non-operated cases). | - |

Table 2: Lesion characteristics and management. PTC=Papillary thyroid carcinoma.

Interobserver agreement statistics and sonographc features are presented in table 3.

| Feature | Inter-observer Kappa | Category | Thy3a-No | Thy3a-Nu | Pearson Chi$^2$ | p-value |
|---|---|---|---|---|---|---|
| Composition | 0.688 | Mixed solid-cystic | 28.6% | 21.1% | 0.30 | 0.58 |
| | | Solid | 71.4% | 78.9% | | |
| Echogenicity | 0.658 | Hyper/isoechoic* | 57.1% | 26.3% | 4.86 | 0.09 |
| | | Hypoechoic | 33.3% | 42.1% | | |
| | | Very hypoechoic | 9.5% | 31.6% | | |
| Mixed echogenicity | N/A | Present | 28.6% | 15.8% | 0.94 | 0.33 |
| | | Absent | 71.4%% | 84.2%% | | |
| Margin | 0.682 | Ill-defined* | 0.0% | 36.8% | 9.72 | <0.05 |
| | | Lobulated | 9.5% | 10.5% | | |
| | | Smooth* | 90.5% | 52.0% | | |
| Direction of growth | 0.654 | Taller than wide | 9.5% | 31.6% | 3.03 | 0.08 |
| | | Wider than tall | 90.5% | 68.4% | | |
| Echogenic foci | 0.501 | None* | 90.5% | 57.9% | 5.83 | 0.05 |
| | | Peripheral calcifications | 0.0% | 5.3% | | |
| | | Punctate echogenic foci* | 9.5% | 36.8% | | |
| Vascularity | 0.531 | Absent* | 0.0% | 21.1% | 15.48 | <0.05 |
| | | Mild intramodular* | 61.9% | 10.5% | | |
| | | Marked intranodular | 19.0% | 15.8% | | |
| | | Unavailable | 19.0% | 42.1% | | |
| Malignancy rate | N/A | No histology | 81.0% | 26.3% | 12.58 | <0.05 |
| | | Benign* | 14.3% | 36.8% | | |
| | | Malignant (PTC and 1 case of NIFTP)* | 4.8% | 36.8% | | |
| TIRADS | N/A | TR2 | 19.0% | 5.3% | 8.27 | <0.05 |
| | | TR3 | 28.6% | 10.5% | | |
| | | TR4 | 47.6% | 47.4% | | |
| | | TR5* | 4.8% | 36.8% | | |
| U-classification | N/A | U2 | 0.0% | 0.0% | 5.35 | 0.07 |
| | | U3 | 81.0% | 47.4% | | |
| | | U4 | 9.5% | 15.8% | | |
| | | U5* | 9.5% | 36.8% | | |

***Significant difference suggested (adjusted residual >1.96)**

Table 3: Sonographic characteristics of thyroid nodules with inter-operator agreement, relative percentages, chi2 and p-values.
PTC=Papillary thyroid carcinoma.
NIFTP=Non-invasive follicular thyroid neoplasm with papillary like nuclear features.

**Conclusion:** Our study found differences in the sonographic features of cytologically indeterminate nodules with differing types of atypia. Those with nuclear atypia were more frequently scored as TIRADS-5 or U5, which correlates with the known increased malignancy risk. Thy3a-Nu lesions were also more likely to have ill-defined margins, contain echogenic foci and demonstrate hypovascularity (underscoring the need to avoid equating increased vascularity with a greater malignancy risk). There was no significant difference in the frequency of mixed echogenicity, suggesting that this feature does not influence the risk of malignancy. Whilst inter-rater agreement was acceptable for several features, it was moderate for others, which highlights the relative subjectivity in the interpretation of sonographic findings. The study was limited by its small sample size and inclomplete histological data and future work with a larger dataset incorporating machine learning techniques would be beneficial.

References:
[1] Cross P, Chandra A, Giles T, Johnson SJ, Poller D., (2024), Guidance on the reporting of thyroid cytology specimens, The Royal College of Pathologists
[2] Poller DN, Baloch ZW, Fadda G, Johnson SJ, Bongiovanni M, Pontecorvi A, Cochand-Priollet B., (2016), Thyroid FNA: New classifications and new interpretations., Cancer Cytopathol. , Jul;124(7):457-66.
[3] Durante C, Hegedüs L, Na DG, Papini E, Sipos JA, Baek JH, Frasoldati A, Grani G, Grant E, Horvath E, Hoang JK, Mandel SJ, Middleton WD, Ngu R, Orloff LA, Shin JH, Trimboli P, Yoon JH, Tessler FN., (2023), International Expert Consensus on US Lexicon for Thyroid Nodules, Radiology, Oct;309(1):e231481.

### The role of MRI radiomics using T2-weighted images and the ADC map for discriminating between Warthin's tumors and malignant parotid gland tumors

*Authors: D.D. Muntean, S.M. Dudea, M. Baciut, C. Dinu, S. Stoia, C. Solomon, C. Csaba, G.M. Rusu, L.M. Lenghel; Cluj-Napoca/RO*

**Purpose/Objectives:** Differentiating between benign and malignant parotid gland tumors (PGT) is essential for establishing the treatment strategy, which is highly influenced by the tumor's histology. The objective of this study was to evaluate the role of MRI-based radiomics in the differentiation between Warthin's tumors (WT) and malignant tumors (MT), two entities proved to present overlapping imaging features on conventional and functional MRI sequences.

**Methods and materials:** This retrospective study involved 125 patients who underwent head and neck MRI scans. A total of 125 PGT with confirmed histology were subjected to radiomic analysis, split into a training group (87 PGT: 55 WT, 32 MT) and a validation group (38 PGT: 23 WT, 15 MT). The radiomic features were extracted from 3D segmentations of PGT performed on the following sequences: PROPELLER T2-weighted images and the Apparent Diffusion Coefficient (ADC) map, using a dedicated software. First- and second-order features were derived for each lesion.

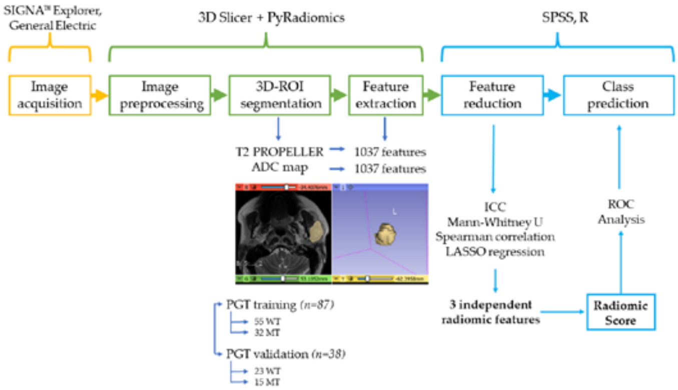

Radiomics workflow diagram

**Result:** After employing several feature reduction techniques, including LASSO regression, three radiomic parameters, were identified to be the most significant in distinguishing between the two studied groups, with the AUC ranging between 0.724-0.747. All three radiomic features were used to construct a radiomic score that presented the highest diagnostic performance in distinguishing between WT and MT, achieving an AUC of 0.786 in the training set, and 0.735 in the validation set.

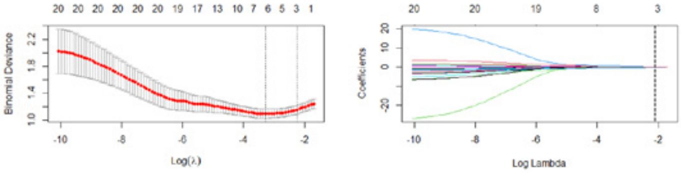

LASSO (least absolute shrinkage and selection operator) regression model

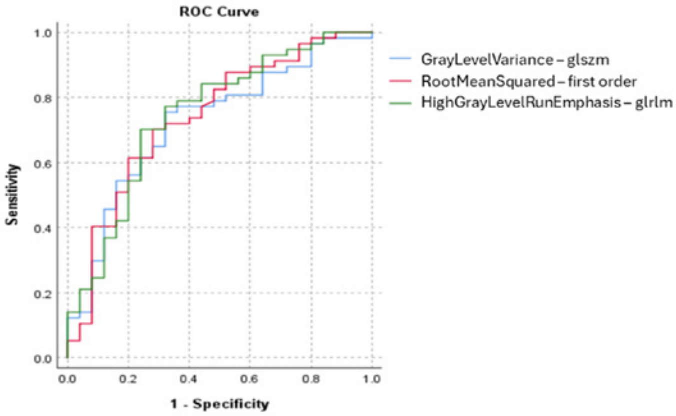

ROC curves of the final selected radiomic features.

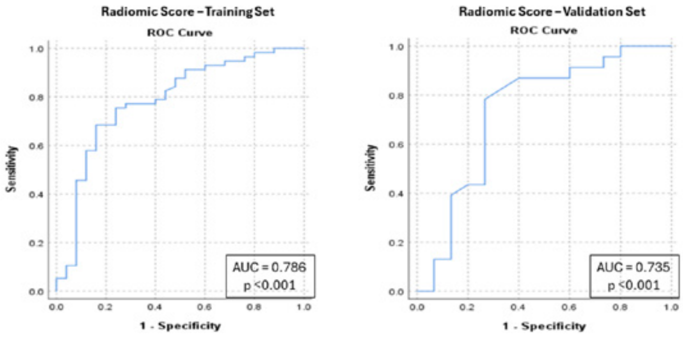

ROC curves of the radiomic score in the training and validation sets

**Conclusion:** MRI-based radiomic features have the potential to serve as additional imaging biomarkers for discriminating between Wartin's tumors and malignant tumors in the parotid gland. However, the validation of the proposed radiomic score in larger, multicentric studies, is warranted.

### The treatment of salivary glands' adenoid cystic cancer with carbon ion radiation therapy and the predictive role of diffusion weighted magnetic resonance imaging – A preliminary study

*Authors:* *M. Pecorilla, S. Imparato, A. Donatelli, L. Brizzi, G. Fontana, M.F. Achilli, C. Fichera, E. Orlandi, L. Preda; Pavia/IT*

**Purpose/Objectives:** This preliminary study aims to investigate how DW-MRI can potentially identify predictive and early biomarkers of disease local control in naïve patients with head and neck adenoid cystic cancer treated with carbon ion radiation therapy.

**Methods and materials:** The clinical and radiological data of 80 patients with diagnosis of head and neck ACC, underwent CIRT between March 2013 and July 2021 at the National Center of oncological hadron-therapy, were retrospectively analyzed. Three trainee radiologists performed manual contouring of the lesions on DW-MRI sequences in baseline and follow-up exams.

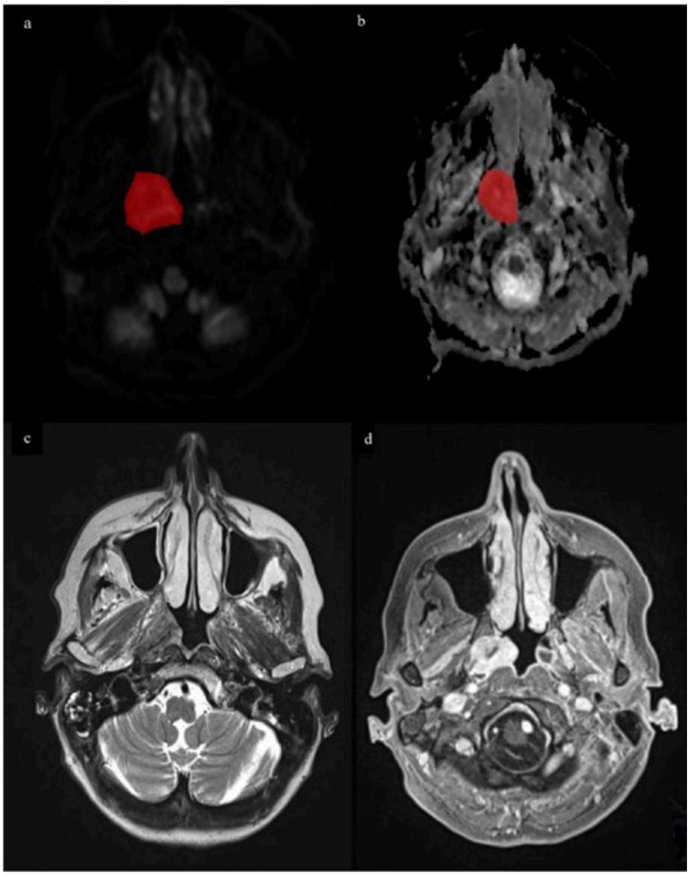

Fig 1. Baseline right nasopharynx lesion of a Responder patient: a. DW image (b 1000) in axial plane and tumor contouring; b. ADC map image in axial plane and tumor contouring; c. TSE T2w image in axial plane d. post mdc T1w image in axial plane.
ADC maps were calculated and first-order histogram features were extracted from the ADC masks: minimum, maximum, mean, median, standard deviation, iqr, kurtosis, skewness. The changes in lesion between baseline and the last follow-up were employed to stratify patients into Responders (Re) and Non Responders (NRe).

**Result:** Between the 80 patients, 41 were eligible, 38 were excluded (13 for no DWi in MR protocol, 3 for poor DWI quality, 22 for missing b-value [necessary b-value: 50, 400, 1000 s/mm$^2$]). Age from 24 to 82 years, with a median of 56.6. ACC onset sites: the nasopharynx (27%), paranasal sinuses (17%), parotid glands (12%), oropharynx/base of tongue (10%), oral cavity (10%), nasal cavity (7%), submandibular glands (5%). The delivered total dose was 68.8 Gy[RBE] (20, 49%); 65.6 Gy[RBE] (39%); 60 Gy[RBE] (10%); 61.6 Gy[RBE] (2,5%). The mean follow-up time was 32 months. Patients were stratified as: 14 NRe and 27 Re. Local PD was reported after 22 months, and the overall sample showed a 92% and 73% local relapse-free survival (LRFS) rate at 1y and 2y. At the univariable analysis ADC baseline skewness (stratified on the median value=0.64; p= 0.043) of first-order features, showed an impact on the LRFS. The lesion site (major vs minor salivary glands) impacted the LRFS (p= 0.045).

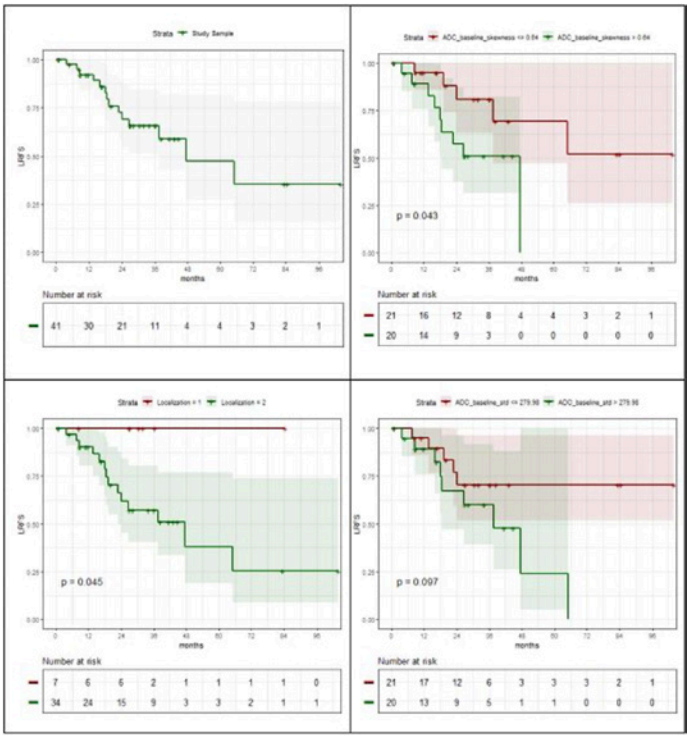

Fig 2. Kaplan-Meier curves: the impact of the ADC baseline skewness and the lesion site on the LRFS.

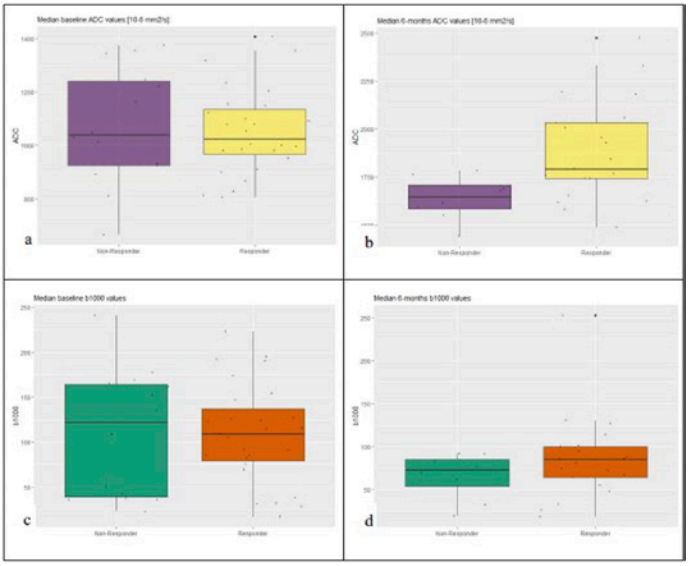

Fig 3. Box plots of ADC and b1000 median values at baseline (a-b) and fup_6months (c-d) in Responders and Non-Responders
ADC mean and median at 6 and 24 months follow-up, reported significant differences among Re and NRe.

| | MWU test: p-value | Re median[IQR]* | NRe median[IQR]** |
|---|---|---|---|
| 6mo_fup_ADC_mean | 0,006 | 1805.79[273.285] | 1639.13[150.804] |
| 6mo_fup_ADC_median | 0,009 | 1792.349[289.676] | 1647.239[125.964] |
| 6mo_fup_ADC_min | 0,3 | 606.942[858.359] | 306.327[611.276] |
| 6mo_fup_ADC_mean | 0,02 | 1760.173[393.075] | 1595.46[67.555] |
| 6mo_fup_ADC_median | 0,03 | 1741.52[439.7] | 1558.478[65.101] |
| 6mo_fup_ADC_min | 0,02 | 774.646[827.766] | 74.219[22.155] |
| 24mo_fup_b1000_kurtosis | 0,03 | 0.931[1.08] | -0.312[0.293] |

Tab1. Features reporting statistically significant differences among Re and NRe, according to Mann-Whitney U (MWU) test.

Re patients reported higher median and mean ADC values after both 6 and 24 months post-CIRT.

**Conclusion:** Pretreatment first order features of ADC values have the potential to become a prognostic imaging marker in patients with head and neck ACC and the assessment of ADC values at short follow up after CIRT could potentially predict medium-to-long-term LR, allowing for a more effective diagnostic and therapeutic management of patients.

### Thyroid Nodule Ultrasound Practice and Preferences; A National Survey

*Authors: N. Sanzone, C. McArthur; Glasgow/GB*

**Purpose/Objectives:** Thyroid nodules are a common incidental finding with studies showing they are present in up to 67% of the population when this region is examined ultrasonically. [1]
Knowing how best to categorise and manage these nodules remains a significant challenge in head and neck radiology.
This survey was designed to gather information across Scotland as to how clinicians working in this field approach the assessment of thyroid nodules. Specific interest was paid to the grading systems which are currently being used and which systems the clinicians would elect to use if they were given the choice.

**Methods and materials:** A survey was created using Microsoft Forms. The survey composed of 20 questions which were a mixture of multiple choice and freetext type. A link was then emailed to all clinicians in Scotland who participate in thyroid imaging. The survey link was active over a 4 week period before being closed and the results gathered.
The results were then reviewed and a mixture of quantitative and qualitative data was collated.

**Result:** There was an 82.6% response rate to the survey with a mixture of radiologists, sonographers and ENT doctors participating.
The only grading systems currently in use in Scotland were found to be the British Thyroid Association Ultrasound "U" Classification[2] and the American College of Radiology Thyroid Imaging and Reporting System[3]. 54.1% of repsondents were using the BTA system and 32.4% were using ACR-TIRADS at the time of the survey. The remaining respondents (13.5%) were using both BTA and ACR-TIRADS in tandem. The average satisfaction score overall was 3.7 out of 5. The average satisfaction in the BTA group was below average at 3.3 out of 5. Satisfaction in the ACR-TIRADS group overall was higher than average at 4.3 out of 5.
Respondents were asked what concerns they had about their current grading system in use. From the BTA group, 65% of the clinicians expressed that "some nodules are too difficult to classify". Only 17% of the ACR-TIRADS group reported this as a concern.

**Conclusion:** This survey has helped to gather useful insights into the current practices and opinions of staff working in head and neck imaging across Scotland. There was a high response rate with most of the health boards in Scotland being represented.
The responses have shown that the overall satisfaction of clinicians was highest amongst those using the ACR-TIRADS system. This fact coupled with the highest number of concerns being raised by users of the BTA system will hopefully open up lines of discussion regarding whether a change needs to be made regarding the current approach to grading thyroid nodules ultrasonically in Scotland.

References:
[1] Cronan JJ, (2008), Thyroid nodules: is it time to turn off the US machines? , Radiological Society of North America, Radiology, 602-4, 247
[2] Perros P, Boelart K, Colley s, et al, (2014), Guidelines for the Management of Thyroid Cancer , Oxford, Clinical Endocrinology, 1 - 122, Volume 81, supplement 1
[3] Franklin N. Tessler, MD, CM, William D. Middleton, MD, Edward G. Grant, MD, Carl C. Reading, MD , Leslie M. Scoutt, MD, (2017), 4.ACR Thyroid Imaging, Reporting and Data System (TI-RADS): White Paper of the ACR TI-RADS Committee, Elsevier, Journal of the American College of Radiology, 587 - 595, Vol 14, issue 5

### Unusual disease with severe condylar resorption: 3 case reports

*Authors:* *J. Kang, J.-E. Kim, K.-H. Huh, M.-S. Heo, S.-S. Lee; Seoul/KR*

**Purpose/Objectives:** This study explores unusual cases featuring distinctive mandibular condyle bone destruction and assesses their radiographic features.
**Methods and materials:**
Case 1: A 58-year-old female presented with bilateral temporomandibular joint pain and limited mouth opening that had been ongoing for a year. Panoramic radiographs and cone-beam computed tomography (CBCT) imaging revealed severe erosion in both mandibular condyles, with almost complete absorption of the condyles and anterior open bite. The patient was referred to the rheumatology department, where a comprehensive evaluation, considering systemic conditions, led to the diagnosis of multicentric reticulohistiocytosis.
Case 2: A 49-year-old female has been experiencing right temporomandibular joint pain that has persisted for one month. Initially diagnosed with TMJ dislocation at a local clinic, she underwent drug therapy for approximately seven months. Follow-up panoramic radiographs and CBCT images revealed a solid mass, accompanied by lateral displacement of the condyle and erosive changes in the articular fossa. Surgical excision was performed, and diagnosed as tenosynovial nodular fasciitis.
Case 3: A 30-year-old female presented with left temporomandibular joint pain and restricted mouth opening that persisting for the one year. Radiographic studies demonstrated bone resorption in the medial and lateral aspect of left condyle, with characteristic periosteal bone reactions extending along the condylar neck to the mandibular ramus. Additional MRI evaluation led to a diagnosis of malignant lesion. The histopathological examination revealed a diagnosis of chondrosarcoma.
**Result:** Erosion of the mandibular condyle and articular fossa in patients complaining of temporomandibular joint pain is a common radiographic finding. Nevertheless, it is essential to consider various diseases based on the erosion pattern, emphasizing the need for differential diagnoses considering factors such as bilaterality, extent of bone destruction, and patterns of periostral bone reaction.
**Conclusion:** This report contributes additional insights into the radiological features of these rare lesions, facilitating the improvement of future differential diagnoses.

Educational Posters

### 4-Hour Delayed-Enhanced 3D FLAIR sequence: Beyond endolymphatic hydrops

*Authors: A. Ben Lakhal, S. Boukriba, W. Frikha, E. Azouz, K. Ben Rhouma, H. Mizouni; Tunis/TN*

**Learning objectives:** Describe, with annotated iconography, the differential diagnoses of endolymphatic hydrops on the delayed-enhanced 3D FLAIR sequence.
Insist on the importance of a complete MRI protocole that includes both the classical sequences and the novel delayed-enhanced 3D FLAIR sequence.
**Background:** The advent of the late-enhanced 3D FLAIR sequence in the last decade is regarded as one of the most important landmarks in head and neck imaging of the recent years.
In fact, thanks to this sequence, it is now possible to visualize, in vivo, a pathological entity that was, until then, only diagnosable on autopsy: endolymphatic hydrops.
A more widespread use of this sequence resulted in the discovery of the MR aspects of other inner ear pathologies that can mimic endolymphatic hydrops both in clinical findings and cochleo-vestibular test results.
Schematically, the MRI findings of these pathologies can be divided in three categories:
1. Diminished endolymphatic spaces
2. Asymmetrical perilymphatic enhancement
3. Pathological enhancement outside of the inner ear

**Imaging findings and procedure details:**
Diminished endolymphatic spaces:
This is found in vestibular atelectasis. Considered for a long-time as an obscure syndrome, it was recently diagnosed in vivo thanks to MR imaging of the membranous labyrinth. It can be divided in two categories: total atelectasis when the utricle and at least two ampullae are not visible and partial atelectasis when these structures are barely visible.

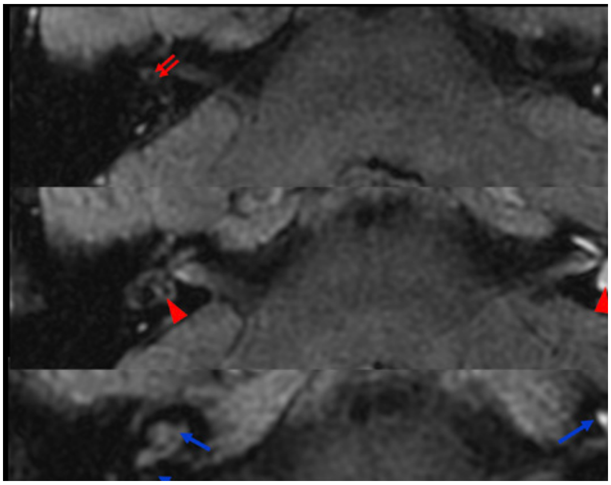

Notice in A, the absence of the left superior semi-circular canal ampulla. In B, the absence of the left utricle. In C, both the saccule and the cochlear canal are visible. Asymmetrical perilymphatic enhancement on the left side indicates a labyrinthitis: this is a secondary vestibular atelectasis.

Asymmetrical perilymphatic enhancement:
A more pronounced perilymphatic enhancement is synonymous with rupture of the haemato-perilymphatic barrier. This is found in two entities: vestibular neuritis and labyrinthitis. In vestibular neuritis, asymmetrical enhancement is usually focal and follows the vascular segmentation of the labyrinth.
Labyrinthitis can be diagnosed on a classical early T1 sequence with contrast injection but the 3D FLAIR sequence has a higher sensitivity.
Pathological enhancement outside the inner ear:
This is found in perilymphatic fistulae in the form of a nodular enhancement adjacent to one of the windows. In the appropriate clinical setting and with correlation to CT and MRI results, it can help make the positive diagnosis of perilymphatic fistulae.

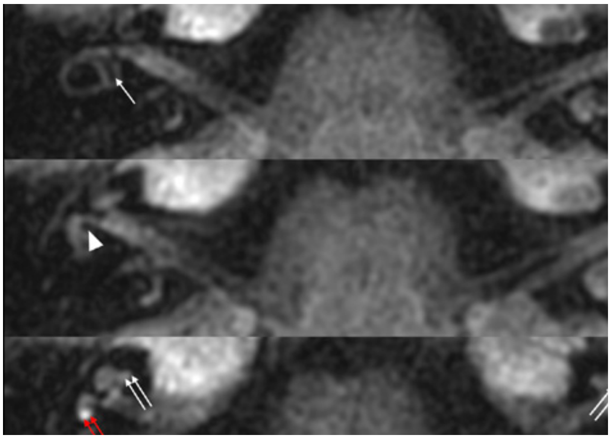

This patient was adressed for a suspicion of a right-sided MD. White arrows and arrowheads on A, B and C show the absence of endolymphatic hydrops. Red double arrows in C show a nodular enhancement adjacent to the round window. Surgery confirmed the perilymphatic fistula.

Importance of a complete protocol:
It is important to correlate the delayed-enhanced 3D FLAIR sequence results with the classical ones. In fact, a vestibular schwannoma, depending on its location, can mimic endolymphatic hydrops on the 3D FLAIR sequence. Correlation with both T2 and post-injection T1 imaging can help avoiding a misdiagnosis.

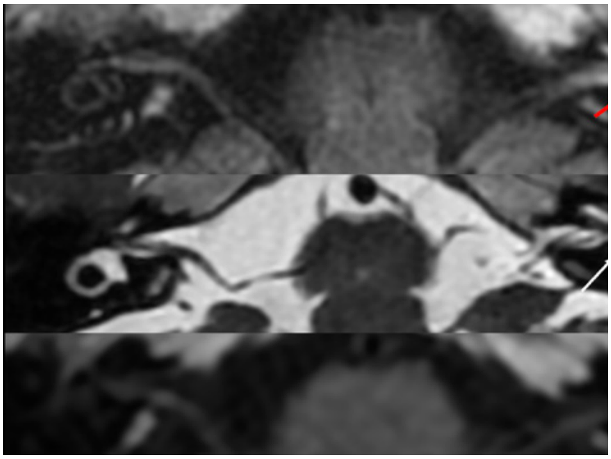

This case highlights the importance of a complete protocol. In A, the signal loss on the delayed-enhanced 3D FLAIR sequence can be misdiagnosed as a utricular hydrops. But in B, it has a low T2 signal and in C, on the T1 post-injection sequence, it shows vivid enhancement: this is a vestibular schwannoma.

**Conclusion:** The late-enhanced 3D FLAIR sequence is a promising imaging tool that can help the radiologist and the otologist make diagnoses of inner ear pathologies beyond the scope of endolymphatic hydrops.

References:
[1] Michael Eliezer et al., (2019), Vestibular atelectasis: Myth or reality? , Wiley-Blackwell , Laryngoscope, 1689-1695, Volume 129, Issue 7, https://onlinelibrary.wiley.com/doi/10.1002/lary.27793, 2019-01-10
[2] Frédérique Dubrulle et al., (2020), The round window sign: a sensitive sign to detect perilymphatic fistulae on delayed postcontrast 3D-FLAIR sequence, Springer Science+Business Media, European Radiology, 6303-6310, Volume 30, https://link.springer.com/article/10.1007/s00330-020-06924-4, 2020-05-28
[3] Lisa M. H. de Pont, (2020), State of the Art Imaging in Menière's Disease. Tips and Tricks for Protocol and Interpretation, Springer Link, Current Radiology Reports, Volume 8, https://link.springer.com/article/10.1007/s40134-020-00365-z, 2020-09-27
[4] Hayoung Byun et al., (2018), Clinical value of 4-hour delayed gadolinium-Enhanced 3D FLAIR MR Images in Acute Vestibular Neuritis, Wiley-Blackwell, The Laryngoscope, 1946-1951, Volume 128, Issue 8, https://onlinelibrary.wiley.com/doi/abs/10.1002/lary.27084, 2018-01-13

### A jaw-dropping tale: Utilising MRI in the management of Juvenile Idiopathic TMJ Arthritis

*Author: R. Carter; Oxford/GB*

**Learning objectives:**
- Understand the role of magnetic resonance imaging (MRI) in the diagnosis and assessment of temporomandibular joint (TMJ) involvement in Juvenile Idiopathic Arthritis (JIA).
- Identify the key imaging findings of TMJ involvement in JIA on MRI, including synovial proliferation, joint effusion, bone erosions, and condylar deformities.
- Recognize the importance of early detection of TMJ involvement in JIA patients using MRI to prevent long-term complications such as mandibular growth disturbances and functional impairments.
- Appreciate the utility of MRI in monitoring disease progression and treatment response in JIA patients with TMJ involvement.

**Background:** As early signs of TMJ involvement in JIA might be difficult to detect clinically, MRI is a valuable tool in its early detection. It is also useful in disease monitoring, in guiding treatment decisions and assessing treatment response.

**Imaging findings and procedure details:** As MRI provides detailed visualisation of soft tissue structures and osteochondral abnormalities, it is key in detecting both acute and chronic manifestations of the disease. Acute findings such as synovial inflammation, joint effusion, bone marrow oedema can be differentiated from chronic findings such as disc abnormalities, erosions, osteochondral changes and mandibular growth abnormalities to assess disease stage and progression.

**Conclusion:** By providing insights into MRI characteristics of TMJ JIA, this pictorial review aims to enhance diagnostic accuracy, facilitate timely intervention, and improve outcomes for paediatric patients with this challenging condition.

### A Pictorial Review of Benign and Malignant Causes of Vocal Cord Palsy

*Authors: S. Arain, S. Vaidyanathan, S. Karthik, A. Zarei; Leeds/GB*

**Learning objectives:**
1. Understand the clinical manifestations and anatomical basis of vocal cord palsy.
2. Review the range of benign and malignant aetiologies which can cause vocal cord palsy with anatomical correlation.
3. Develop a comprehensive imaging search strategy for the evaluation of symptomatic or incidentally detected vocal cord palsy.

**Background:** Vocal cord palsy, typically unilateral, often stems from pathology affecting the ipsilateral recurrent laryngeal nerve, a branch of the vagus nerve. This condition can manifest with symptoms such as dysphonia, dysphagia or dyspnoea and may signify a serious underlying disease. Despite this, up to 40% of cases may be asymptomatic and discovered incidentally during imaging performed for other reasons or on nasoendoscopy.

**Imaging findings and procedure details:** Imaging serves as a crucial tool in the evaluation of vocal cord palsy, particularly when a cause is not clinically apparent. This educational poster provides a comprehensive pictorial review, showcasing various aetiologies which can cause vocal cord palsy and correlates them with the anatomical course of the vagus and recurrent laryngeal nerves. By understanding the anatomical relationships and pathological processes, radiologists can develop an effective search strategy for the evaluation of symptomatic or incidentally detected vocal cord palsy.

**Conclusion:** An understanding of the clinical manifestations, potential aetiologies, anatomical basis, and imaging findings associated with vocal cord palsy is essential for healthcare professionals. This knowledge enables the development of an effective imaging search strategy for timely diagnosis and appropriate management of benign and malignant conditions causing vocal cord palsy.

### A rare case of neck region ganglioneuroma in a 2-year old girl

*Authors: B. Valente[1], L. Gambardella[2], F. Malacario[3], A. Manto[3]; [1]Napoli/IT, [2]Campobasso/IT, [3]Nocera Inferiore/IT*

**Learning objectives:**
The purpose of this work is to describe the radiological findings of ganglioneuroma and to provide guidance on the differential diagnosis of neck masses in pediatric age, as well as to present a rare case of neck region ganglioneuroma in a 2-years-old girl, which has never been reported in literature at this age.

**Background:** Ganglioneuroma (GN) represents the least aggressive form of neuroblastic tumors and it is derived from cells of the autonomic nervous system, typically localized in the posterior mediastinum, retroperitoneum or adrenal gland[1][2]. Although rare, GN can also occur in the cervical region, accounting for only a small percentage of cases. This tumor mainly affects children and young patients with median age of 7 years old[2]. Often asymptomatic, GN may present with symptoms related to compression of adjacent structures as it grows. Diagnostic imaging including ultrasound, CT and MRI, plays a crucial role in identifying the location, size and composition of the lesion, guiding treatment decisions[2][3].

**Imaging findings and procedure details:** In this case, a 2-year-old girl presented with asymptomatic palpable mass in the left lateral neck. Ultrasonography revealed an oval mass behind the left mandibular corner, with wavy margins, a predominantly solid echostructure with millimetric contextual calcification and small internal vascular spots.

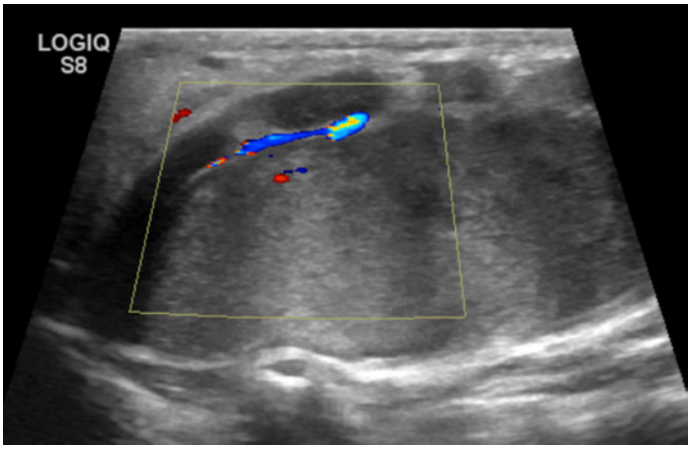

Figure 1 - Doppler ultrasonography shows a solid oval mass with mixed echostructure and some small internal vascular spots, found in the left lateral side of the neck.

Contrast-enhanced MRI provided further details, showing a voluminous expansive lesion (64x29x44 mm) with regular and well defined limits, signal characteristics suggesting hypocellularity and marked and homogeneous contrast enhancement.

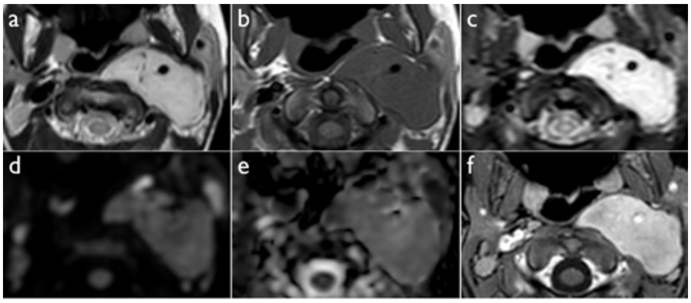

Figure 2 - MRI images showing the presence of a voluminous expansile lesion with well-defined margins located in the left carotid space of the neck. It is characterized by hyperintense signal on T2w (a) and STIR (c) sequences, hypointense signal on T1w (b), poor signal restriction on DWI (d)/ADC map (e) and intense and homogeneous enhancement (f).

The mass occupied the carotid space, compressing adjacent structures, displacing surrounding tissues, and causing "encasement" of the ipsilateral ICA. Imaging and clinical-laboratory findings led to the exclusion of congenital developmental anomalies and infectious inflammatory causes, in favor of a more probable neoplastic etiology. Some differential diagnosis we considered were paraganglioma, neurofibroma, rhabdomyosarcoma or a neuroblastic tumor. The little patient underwent surgery and after excisional biopsy she was diagnosed with ganglioneuroma.

**Conclusion:** GN is a rare tumor that can occur in various locations, including the cervical region, although infrequently. Imaging modalities (such as US and MRI) and histopathological examinations are invaluable for differential diagnosis and for characterizing GNs, aiding in treatment planning. Prognosis is usually excellent, however local recurrence has been reported.

References:
[1] Wu Z, Zeng Q, Zhang X, Tu S, Zhang F., (2018), Ganglioneuroma in unusual sites: clinical, radiologic and pathological features., International journal of clinical and experimental pathology, 4862–4866, 10, https://www.ncbi.nlm.nih.gov/pmc/articles/PMC6962920/, 2018-10-01
[2] Lonergan, G. J., Schwab, C. M., Suarez, E. S., & Carlson, C. L, (2002), Neuroblastoma, ganglioneuroblastoma, and ganglioneuroma: radiologic-pathologic correlation, RadioGraphics, 911–934, 4, https://doi.org/10.1148/radiographics.22.4.g02jl15911, 2024-07-01
[3] Xu, T., Zhu, W., & Wang, P. , (2019), Cervical ganglioneuroma: A case report and review of the literature , Medicine, Baltimore, 15, https://doi.org/10.1097/MD.0000000000015203, 2024-04-01

### A rare case of unilateral cervical pain: Translent perivascular inflammation of the carotid artery (TIPIC) syndrome, Carotidynia

*Authors: S. Günes, G. Kılıçarslan; Elazıg/TR*

**Learning objectives:** Transient perivascular inflammation of the carotid artery (TIPIC) syndrome is a rare and idiopathic syndrome that occurs with unilateral neck pain, marked tenderness, and over the carotid artery bifurcation. Our aim is to consider this syndrome in patients presenting with unilateral pain and help rule it out from other differential diagnoses.
**Background:** TIPIC syndrome is a rare syndrome also known as carotidynia. It often affects individuals in the 4th and 5th decades and is more common in women than men. Although its etiology is not fully known, it is assumed to occur from infectious or inflammatory processes affecting the vascular wall [1],[2]. Clinical findings include local pain at the level of the carotid artery with irradiation towards the neck and face, local swelling, lymphadenopathy, fever, headache [3]. In particular, cross-sectional imaging also plays an important role in the diagnosis of other vascular pathologies such as arterial dissection, large vessel arteritis, arteriosclerosis, fibromuscular dysplasia or aneurysm that present with unilateral neck pain and mimic TIPIC syndrome [4]. Radiological imaging is of great importance in the diagnosis of the disease. Correct diagnosis is essential in dictating the proper management.
**Imaging findings and procedure details:** In our case, a 44-year-old female patient applied to the outpatient clinic with left neck pain. Ultrasonography (US) is generally the first method with low cost and high sensitivity to detect pathology in superficial tissues. Ultrasound scan of the neck using 9 MHz linear array transducer revealed decreased echogenicity and eccentring intimal thickening of distal common carotid artery and carotid bulb on left side of the neck. Although there was no serious narrowing in the lumen, eccentric intimal thickening was observed (Figure 1).

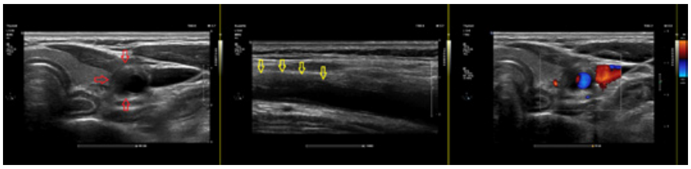

Figure 1: US Findings: Intimal thickening

It is necessary to rule out other vascular pathologies with MRI or CT imaging. In our case, MRI was preferred and there was intimal thickening, edema and increased contrast enhancement (Figure 2).

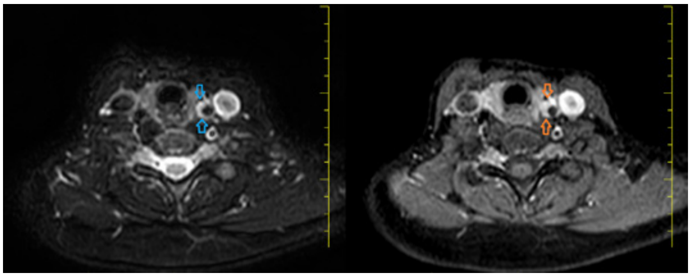

Figure 2: MRI Findings: intimal thickening, oedema and contrast enhancement

**Conclusion:** Carotidynia is a one of the very rare cases. The important thing is to remember this entity in cases presenting with neck pain and to diagnose it with the necessary imaging techniques. It is mostly a self-limiting condition and nonsteroidal anti-inflammatory drugs (NSAIDs) are effective in treatment.

References:
[1] Moreira MCS, Dos Santos AC, Cintra MB. , (2017), Perineural spread of malignant head
[1] Mathangasinghe Y, Karunarathne RU, Liyanage UA, (2019), Transient perivascular inflammation of the carotid artery; a rare cause of intense neck pain, BJR Case Rep., 10.1259/bjrcr.20190014
[2] Baig MA, Asad S, (2023), Carotidynia presenting as an atypical cause of unilateral neck pain in the emergency department, Oxf Med Case Reports, 10.1093/omcr/omad094
[3] Peycheva M, Zdravkova T, Zlatareva D, Viteva E, Harizanova Z, Meinel TR, Heldner MR, (2024), Transient perivascular inflammation of the carotid artery-A transient but potentially recurrent disease, Clin Case Rep, 10.1002/ccr3.8322
[4] Santarosa C, Stefanelli S, Sztajzel R, Mundada P, Becker M, (2017), Santarosa C, Stefanelli S, Sztajzel R, Mundada P, Becker M, Case Rep Radiol., 10.1155/2017/7086854

### A Rare Case: Extracranial Internal Carotid Artery Aneurysm

*Authors: G. Kılıçarslan, Y. Gül; Elazıg/TR*

**Learning objectives:** Peripheral arterial aneurysms are rare and extracranial carotid artery aneurysms are even rarer. They have various causes of occurrence and usually present with swelling or pulsatile mass. The aim of this poster is to present a patient with isolated extracranial internal carotid artery aneurysm and to provide general information.
**Background:** Isolated extracranial internal carotid artery aneurysms account for less than 1% of all peripheral arterial aneurysms. It is defined as an increase of more than 50% of the reference size (1). People with this abnormality, which can cause various neurological symptoms, usually present to hospitals with complaints of swelling and pulsatile mass in the neck. Cerebral ischemia or 10th cranial nerve symptoms may occur due to compression of large aneurysms. Transient ischemic attack, dizziness, tinnitus, and pain are other common symptoms. Trauma, inflammation, congenital, previous operations, radiotherapy, fibromuscular dysplasia and atherosclerosis have been shown to be involved in the pathogenesis (1-3) The most common cause is atherosclerosis (2). It is classified as true or pseudoaneurysm according to the part of the vessel involved (1). Imaging methods are used to confirm the diagnosis. The location, etiology and morphology of the ICAA are important in determining the appropriate treatment (3-5).
**Imaging findings and procedure details:** Doppler USG is the first method of choice. CT examinations will give us information about the shape of the aneurysm. It will also show calcifications very clearly. Typically, arterial contrast enhancement is observed after contrast material is administered. MR angiography and Digital substraction angiography are also widely used in the diagnosis. Eggshell calcification is more commonly seen in fusiform type aneurysms. Calcification in the aneurysm wall indicates a true aneurysm. True aneurysms, mostly caused by atherosclerosis, are localized dilatation of the arterial diameter with integrity of the three vascular layers. Our case was a 66-year-old male. There was no known history of trauma or surgery. There were atheroma plaques in the arterial walls. We thought that the etiology was due to atherosclerosis, which is the most common cause. There was an aneurysm in the left ICA, 24 mm distal to the carotid bifurcation, with coarse calcifications in the wall and compression of the jugular vein.

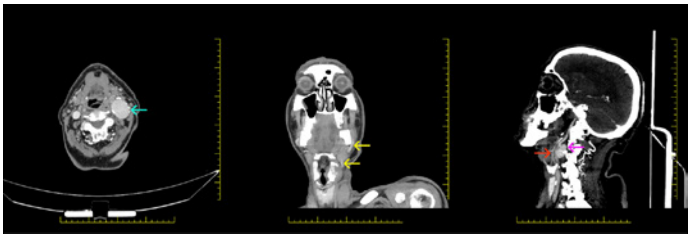

The calcifications on the aneurysm wall are shown with blue arrows, the relationship of the ICA to the aneurysm is shown with yellow arrows, and the ECA is shown with a red arrow.

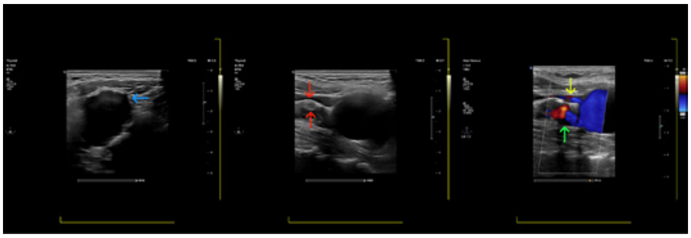

B-mode and color doppler USG, wall calcifications are shown with blue arrow, atheroma plaque causing 50% stenosis in the proximal part of the aneurysm is shown with red arrows, ICA is shown with green arrow, ECA is shown with yellow arrow.

**Conclusion:** Extracranial carotid artery aneurysms are rare. However, they are of medical importance due to their location, differential diagnosis, natural history, complications and treatment. The appropriate treatment method should be chosen considering the localization and size of the aneurysm. Therefore, correct interpretation of radiologic images will be guiding.

References:
[1] Bilreiro C, Grande P, Brito J, Silva LD, Aleixo F. , (2013), Extracranial internal carotid artery aneurysm. , Eurorad, DOI:10.1594/Eurorad/CASE.11350, ISSN:1563-4086.
[2] Ak K, кsbir S, Bayramiçli M, Tekeli A, Bozkurt S, Arsan S., (2009), Aneurysm of Extracranıal Internal Carotıd Artery: a case report. , Marmara Medical Journal
[3] Welleweerd JC, den Ruijter HM, Nelissen BG, Bots ML, Kappelle LJ, Rinkel GJ, Moll FL, de Borst GJ. , (2015), Management of extracranial carotid artery aneurysm, Eur J Vasc Endovasc Surg, doi: 10.1016/j.ejvs.2015.05.002.
[4] Davidovic L, Koncar I, Dragas M, Ilic N, Banzic I, Pavlovic SU, Markovic M, Ristanovic N. , (2016), Treatment strategies for carotid artery aneurysms, J Cardiovasc Surg , Torino
[5] Pourier VE, De Borst GJ., (2015), Which carotid artery aneurysms need to be treated (and how)? , J Cardiovasc Surg, Torino

### A segmental approach to facial nerve pathologies – A pictorial review

*Authors:* *J. Asogan Vaishnavi[1], S. Thiru[1], A. Kamalasanan[2]; [1]Dundee/GB, [2]Glasgow/GB*

**Learning objectives:**
1. Normal course and radiological anatomy of facial nerve
2. Clinical presentation of facial nerve disorders
3. Role of imaging in evaluation of facial nerve
4. Pictorial review of facial nerve pathologies with a segmental approach

**Background:** The facial nerve is one of the most complex cranial nerves and is also one of the most commonly paralysed nerves in the body. The main intent of this exhibit is to familiarise the readers with the segmental imaging anatomy of the facial nerve and the common pathologies affecting each of these segments with special emphasis on clinical presentation which in turn helps with tailoring the imaging technique. Our educational exhibit compiles all the causes and imaging findings on a segmental basis, under one heading via a structured pictorial review.

**Imaging findings and procedure details:** Facial nerve disorder can occur from a variety of causes, including congenital, inflammatory, traumatic, vascular, and neoplastic processes. Ideally, CT is most useful for evaluation of the facial nerve segments contained within the temporal bone, while contrast enhanced MRI is most appropriate for the evaluation of the intra-axial, cisternal, and extra-cranial segments. CT and MRI protocols can be tailored according to the segment of facial nerve being evaluated.

Tailoring the imaging technique based on the clinical presentation can be adapted as follows:
1. Central lesion suspected (presence of a paralysis of muscles of facial expression with forehead sparing) - MRI is indicated, looking for ischemic, demyelinating or tumour lesions in the intra-axial facial nerve pathways above the facial nerve nuclei.
2. Peripheral lesion suspected (paralysis of ipsilateral muscles of facial expression +/- impairment of lacrimation, sound dampening and taste), a temporal bone lesion should be excluded, best evaluated by HRCT temporal bone.
3. Facial nerve tumour evaluation – Both MRI and temporal bone CT are typically performed and are complimentary to each other.
4. Facial palsy cannot be definitively localized – Contrast-enhanced MRI.
5. Parotid segment evaluation – MRI

**Conclusion:** The appropriate radiological evaluation in facial neuropathy requires a thorough knowledge of its complex segmental anatomy and its various pathologies along with a good clinical knowledge.

### A survival guide for young radiologists in the assessment of nasopharyngeal cancer: What to look for and how to report

*Authors:* *S. Galioto, F. Tiralongo, L. Reali, F. Marino, P.V. Foti, S. Palmucci, A. Basile; Catania/IT*

**Learning objectives:** Malignant tumors of the nasopharynx are mainly represented by nasopharyngeal carcinoma (NPC), a tumor of epithelial origin corresponding to more than 90% of malignant tumors that develop in the nasopharynx.
This study aims to provide a simple guide to MRI staging and reporting in NPC.

**Background:** Nasopharyngeal cancer represents the 22nd most common cancer globally.
Patients usually present clinically with hearing loss, nasal obstruction, anosmia, epistaxis, trismus, and swallowing (due to invasion of the XII cranial nerve) or dysphonia (X), diplopia (VI), or enlarged cervical lymph nodes.
After rhinoscopy and biopsy, following the finding of an NPC, the patient undergoes MRI evaluation.

**Imaging findings and procedure details:** MRI represents the technique of choice, superior to CT, for assessing the NPC, providing information on the location and extension of the tumor and on the nodal involvement, to plan the radiotherapy strategy and the need for chemotherapy.
MRI report must contain the size of the primary tumor and the local staging, from the tumor localized to the nasopharynx, nasal cavity, or oropharynx (T1) to the invasion of the surrounding para- and retro-pharyngeal soft tissues (T2), the skull base, paranasal sinuses, and cervical spine (T3), up to the involvement of cranial nerves, orbits and hypopharynx (T4); we also propose an anatomical checklist for assessment of local tumor extension and a focus on the MRI findings of perineural spread.

| FRONT EXTENSION | POSTERIOR EXTENSION | CAUDAL EXTENSION | CRANIAL EXTENSION | LATERAL EXTENSION |
|---|---|---|---|---|
| Coana Space | Retropharyngeal space | Oropharynx | Foramen Lacerum Anterior | Pharyngo-Basilar Fascia |
| Ethmoid | Carotid space | Hypopharynx | Foramen Ovale | Parapharyngeal Space |
| | Prevertebral space | | Clivus | Masticatory Space |
| | Atlo-axial junction | | Sphenoid Sinus | Infratemporal Fossa |
| | | | Meckel's cavus | Pterygopalatine Fossa |
| | | | Cavernous sinus | Fissures Orbital/Orbit |
| | | | | Parotid Space |

Anatomic checklist of loco-regional invasion.

Nodal staging (N1-N3) includes accurate lymph node level localization and using NODE-RADS criteria to better identify suspected nodal involvement[1].

**Conclusion:** MR imaging maps the sites and boundaries of the primary tumor and nodal metastases when planning for radiation therapy, and it also provides indicators of the need for chemotherapy.

References:
[1] Elsholtz FHJ, Asbach P, Haas M, Becker M, Beets-Tan RGH, Thoeny HC, Padhani AR, Hamm B. Introducing the Node Reporting and Data System 1.0 (Node-RADS): a concept for standardized assessment of lymph nodes in cancer. Eur Radiol. 2021 Aug;31(8):6116-6124. doi: 10.1007/s00330-020-07572-4. Epub 2021 Feb 14. Erratum in: Eur Radiol. 2021 Mar 19;: PMID: 33585994; PMCID: PMC8270876.

### Abnormalities of the terminal thoracic duct: No need for these to be a diagnostic pain in the neck

*Authors: C. Vincent, S. Karthik; Leeds/GB*

**Learning objectives:**
1. Review relevant anatomy of the terminal thoracic duct.
2. Illustrate the imaging features of the terminal thoracic duct in different imaging modalities.
3. Review a case series of patients presenting with left neck swelling related to the duct to raise awareness of this potential pitfall in head and neck imaging.

**Background:** Lower neck swelling is a common presentation in the general population. (1) Diagnosis can prove challenging because of myriad potential aetiologies. The radiologist plays a key role in helping clinicians identify “don’t touch” findings e.g. anatomical structures and benign lesions, crucial in averting unnecessary intervention. The terminal thoracic duct is an anatomical structure that can present as a left supra-clavicular fossa swelling or mass when dilated or, occasionally, inflamed. If not recognised this can lead to misidentification as a ‘pathological’ lesion at clinico-radiological assessment thus potentiating inappropriate management and potentially serious complications such as chyle leak. (2)(3) This poster aims to provide, in conjunction with a small case series, an overview of the anatomy of the terminal thoracic duct and its abnormal manifestations illustrating key multi-modality imaging features to assist identification of this rare but significant cause of left neck swelling.

**Imaging findings and procedure details:** We will exhibit ultrasound, computed tomography, and magnetic resonance imaging examples from a selection of cases where the cause of left lower neck swelling was ultimately determined to be related to the terminal thoracic duct. Given its high spatial resolution, dynamic nature and the relative ease of imaging guided targeted concurrent sampling, ultrasound is the ideal problem-solving modality in these cases and will be the focus. In each case the initial imaging differential provided was incorrect, and the possibility of findings being related to the terminal duct were only raised on subsequent examinations, highlighting this as a potential pitfall. All cases have undergone sampling with cyto/histopathological, biochemical and/or microbiological correlation or imaging follow-up.

**Conclusion:** This poster will be relevant to healthcare professionals with an interest in head and neck imaging and will increase awareness of this potential diagnostic pitfall in patients presenting with swelling of the left lower neck.

References:
[1] Quraishi S, Bradley PJ, (2007), Neck Swellings, Ear, Nose and Throat, 10:87
[2] Seeger M, Bewig B, Günther R, Schafmayer C, Vollnberg B, Rubin D, Hoell C, Schreiber S, Fölsch UR, Hampe J, (2009), Terminal part of thoracic duct: high-resolution US imaging, Radiology, 897-904, 252(3):
[3] Liu ME, Branstetter IV BF, Whetstone J, Escott EJ, (2006), Normal CT appearance of the distal thoracic duct, American journal of roentgenology, 1615-20, 187(6)
[4] Offiah CE, Twigg S, (2011), Lymphocoele of the thoracic duct: a cause of left supraclavicular fossa., The British journal of radiology, 27-30, 84(998)

### Approaching External Ear Pathologies: A Case-Based Perspective

*Authors: M.S. Swarup, P.N.S. Varalakshmi, M. Jain, P. Gupta, R. Kanaujia, R. Gupta Ghasi; New Delhi/IN*

**Learning objectives:**
1. To provide a case based review of various common and uncommon pathologies affecting the external ear
2. To discuss the role of imaging in each case and information provided by cross sectional imaging studies beyond clinical examination
3. To emphasize the HRCT and MR imaging characteristics of abnormalities relevant to radiologists
4. To briefly discuss the imaging anatomy and embryological developement of external ear for better understanding of pathologies

**Background:** The external ear, composed of the pinna and the external auditory canal (EAC) can be affected by a multitude of abnormalities. These conditions include congenital anomalies, infective and inflammatory pathologies, traumatic injuries, vascular malformations, and benign and malignant neoplasms. Some of these pathologies are amenable to physical examination and can be diagnosed clinically. However, clinical evaluation and diagnosis is not always possible due to obscuration of deeper aspect by the lesions itself and similar clinical features and presentation in some cases. Hence, imaging is often necessary to assess the extent of a lesion, determine its rectectability, plan surgical management, formulate differential diagnoses, and to evaluate potential complications.

**Imaging findings and procedure details:** HRCT scans are typically the initial diagnostic method for assessing the external auditory canal and are superior in revealing bone changes. On the other hand, MRI excels in tissue characterization and allows for better delineation of lesion extension and perineural tumor spread.
The following pathologic entities of the external ear will be discussed with a case based approach focusing cross-sectional imaging features, radiologists should know.

1. *Congenital external ear malformations (Auricle and EAC malformations including EAC stenosis and complete atresia)*
2. *First branchial cleft anomalies*
3. *Otitis externa*
4. *Necrotising/ Malignant Otitis externa and Osteomyelitis*
5. *Polychondritis of external ear*
6. *Keratosis obturans*
7. *Medial canal fibrosis*
8. *EAC cholesteatoma*
9. *Vascular malformations - Slow flow and High flow*
10. *EAC osteoma and exostosis*
11. *Pyogenic granuloma/capillary hemangioma of external ear*
12. *Glomus tympanicum extending to EAC*
13. *Primary malignant neoplasm of external ear – Squamous cell carcinoma*
14. *Secondary malignant involvement from direct tumor spread from adjacent periauricular skin, parotid gland, or regional lymph nodes*
15. *EAC injuries and fractures*
16. *EAC foreign bodies and wax/cerumen*

**Conclusion:** Although clinical examination is sufficient in some cases, Imaging is often necessary for complete evaluation. Understanding the embryologic development, anatomy of the external ear, and awareness of common and less common associated conditions are crucial for precise radiologic diagnoses.

### Arts and Signs in Head and Neck Emergencies

*Authors: G.W. Tan, X.Y. Tan, C.L. Ho; Singapore/SG*

**Learning objectives:**
This pictorial review aims to illustrate classical imaging features of various head and neck conditions in emergency settings. Pertinent radiological signs and the cause for their appearances will be discussed.
**Background:** Head and neck emergencies are life-threatening conditions. By associating key radiological features with common daily objects, radiologists apply pattern recognition to rapidly identify the pathognomonic appearance of various pathological conditions. Known as the "Aunt Minnie" phenomenon, the use of these imaging signs to confidently establish accurate diagnoses are invaluable in the emergency settings when time is of essence.
**Imaging findings and procedure details:** We have a collection of unusual head and neck imaging signs following retrospective review of radiological images of patients admitted to the Emergency Department of a tertiary hospital in Singapore from 2018-2023. These images span over a wide range of conditions including trauma, infection, vascular, neoplastic, congenital and degeneration. Several of the case examples are highlighted below.
Iatrogenic/Trauma

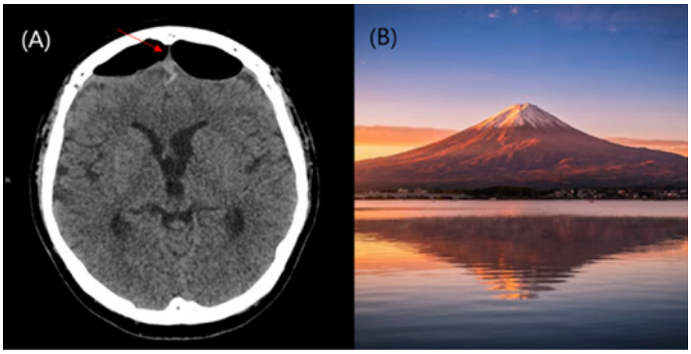

Fig. 1A. An axial CT brain of a 74-year-old man following bifrontal craniotomy shows an accumulation of air in the subdural space causing compression of the underlying brain parenchyma, forming a peak in the midline or "peaking" sign (arrow).
The air under pressure resulted in separation of the frontal lobes and widening of the interhemispheric space. This appearance resembles the silhouette of Mount Fuji (Fig 1B) hence earning the name 'Mount Fuji sign'. The air under pressure can be relieved by reopening of the cranioplasty with saline wash-out.
Infection

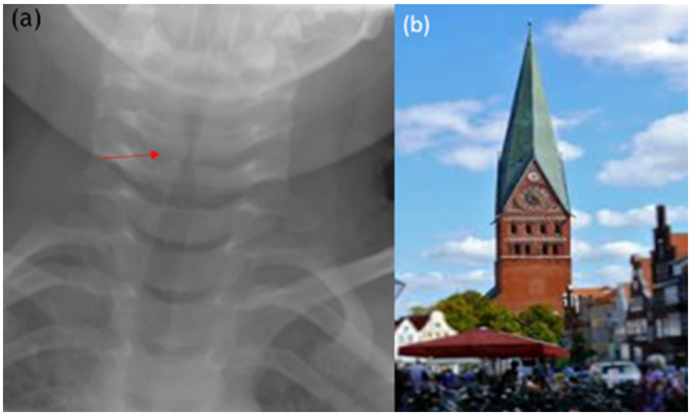

A 4-year-old boy presents with a barking cough, hoarseness of voice and inspiratory stridor. He has been diagnosed with croup, possibly caused by the parainfluenza virus. Fig 2A. A frontal chest radiograph shows narrowing and tapering of the subglottic larynx and upper trachea (arrow) due to oedema of the upper airways.
The appearance resembles the church steeple (Fig 2B) hence earning the name 'Steeple sign'. Treatment is usually supportive; in severe cases, corticosteroids are adminstered to reduce airway inflammation.
Vascular

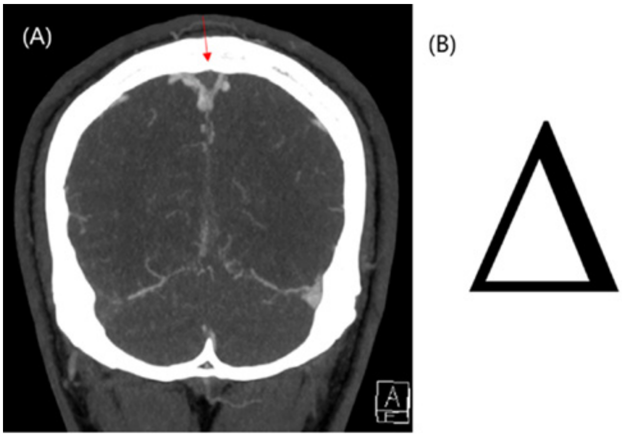

A 32-year-old female presents with severe headache. Fig. 3A. Coronal contrast-enhanced CT brain shows a triangular filling defect in the superior sagittal sinus (arrow), resembling the delta symbol, hence earning the name "empty delta sign". Treatment includes thrombectomy and anticoagulation to prevent progression to venous infarction.
Neoplastic

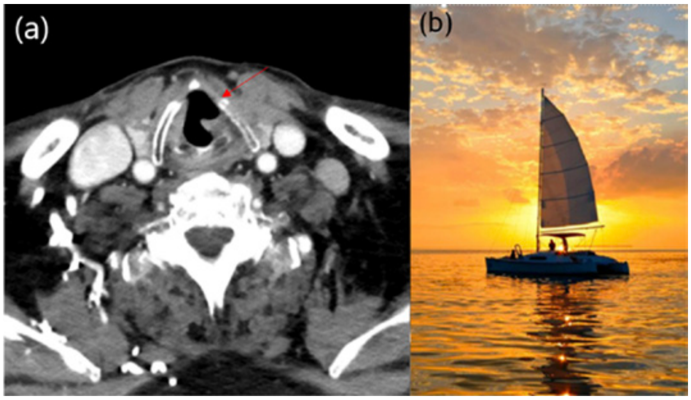

Fig 4A. Axial T1-weighted contrast-enhanced MRI neck of a 86 year-old man with left vocal cord palsy shows dilated left laryngeal ventricle and pyriform fossa (arrow); the shape resembling the sail of a boat (arrow) hence earning the name 'sail sign' (Fig 4B).
It is essential to exclude an extrinsic compression of the recurrent laryngeal nerve on imaging.
**Conclusion:** Prompt recognition of key radiological signs is paramount in emergency head and neck imaging when time is of essence.

### Benign tumors and tumor-like lesions of the tongue: A pictorial review

*Authors:* *M. Pietragalla[1], C. Nardi[2], A. Lo Casto[3]; [1]Pistoia/IT, [2]Florence/IT, [3]Palermo/IT*

**Learning objectives:**
- To review the main radiological features of benign tumors and tumor-like lesions occurring in the tongue.
- To highlight the role of imaging in the diagnosis of these pathological entities.

**Background:** Benign tumors or tumor-like lesions of the tongue are uncommon and comprise a heterogeneous group of tongue alterations[1]. CT and MRI are mandatory for the diagnostic approach of these lesions, and imaging features can be used to narrow the differential diagnosis. Benign tumors or tumor-like lesions of the tongue described herein include schwannoma, lipoma, angiomyolipoma, lymphatic, venous and arterio-venous malformation (AVM), thyroglossal duct remnants, dermoid and epidermoid cysts.

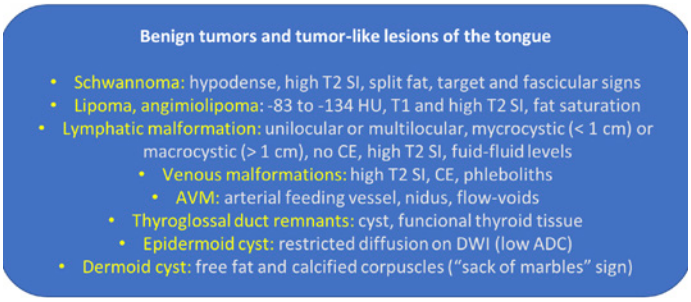

Benign tumors and tumor-like lesions of the tongue

**Imaging findings and procedure details:** Schwannoma usually arises from the anterior two-thirds of the tongue, and very rarely from the tongue base. On CT schwannoma appears as a well-defined and hypodense mass. On MRI it demonstrates low signal intensity (SI) on T1, high SI on T2, and intense contrast enhancement (CE).

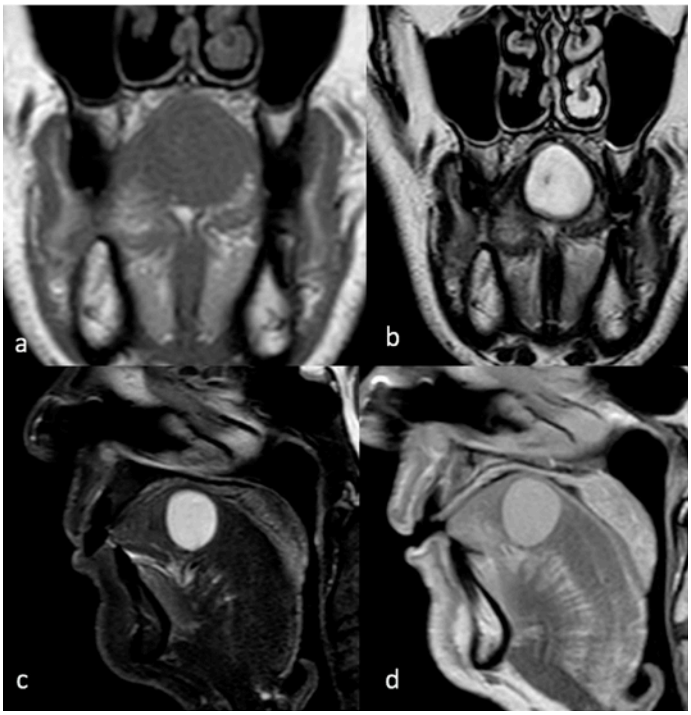

Tongue schwannoma in a 60-year-old male patient. MRI reveals a rounded, well defined mass in the tongue's body that displaces surround fat tissue, showing T1 intermediate SI (isointense to muscle) (a), T2 (b) and STIR high SI without intralesional fat (c). After gadolinium contrast injection, the mass shows vivid and homogeneous enhancement (d).
Split fat sign, target sign, and fascicular sign may help the diagnosis on MRI. Cystic and fatty degeneration may occour[2].

Intramuscular lipoma of the tongue displays mature adipocytes that often irregularly infiltrate and entrap muscle fibers. CT and MRI show an homogenous soft tissue mass with the same density and signal of subcutaneous fat (-83 to -134 Hounsfield units, high SI on both T1 and T2, homogeneous fat saturation signal, and no CE)[3]. Angiomyolipoma appears more heterogenous than lipoma, especially on contrast imaging due to vascular and muscular components.
Congenital vascular malformations include lymphatic (low-flow), venous (low flow) and artero-venous (AVM, high flow) malformations. Lymphatic malformations are unilocular or multilocular, mycrocystic (< 1 cm) or macrocystic (> 1 cm), nonenhancing alterations with high SI on T2. Fluid-fluid levels may occur in case of hemorrhage due to rapid grow[4]. Venous malformations usually enhance and contain phleboliths. Veno-lymphatic malformations share characteristics of both lymphatic and venous malformations. AVM are typically characterized by an arterial feeding vessel, a nidus, and flow-voids on MRI.
Thyroglossal duct remnants are congenital lesions that arise from anomalous development and migration of the thyroid gland. These remnants may accumulate fluid and be referred to as thyroglossal duct cysts or may be represented by functional thyroid tissue.
Epidermoid cyst shows restricted diffusion on DWI, whereas dermoid cyst contains free fat and/or calcified corpuscles ("sack of marbles" sign) hyperdense on CT and hypointense on T2w MRI[5].
**Conclusion:** Characteristic CT and especially MRI features can supply a clue for differential diagnosis of tumors and tumor-like lesions of the tongue.

References:
[1] Liu, L., Li, Y., Zi, Y. et al., (2023), MRI findings of benign tumors and tumor-like diseases of the tongue with radiologic–pathologic correlation, Jpn J Radiol, 19–26, 41, https://doi.org/10.1007/s11604-022-01329-3
[2] Badar Z, Farooq Z, Zaccarini D, Ezhapilli SR, (2016), Tongue base schwannoma: differential diagnosis and imaging features with a case presentation, Radiol Case Rep., 336-340., Nov 9;11(4), 10.1016/j.radcr.2016.10.001
[3] Yoon YA, Kwon YE, Choi SY, Choi KS, An CH, An SY, (2021), Giant lipoma of the tongue: A case report and review of the literature, Imaging Sci Dent., 117-121, Mar;52(1), 10.5624/isd.20210140
[4] White CL, Olivieri B, Restrepo R, McKeon B, Karakas SP, Lee EY, (2016), Low-Flow Vascular Malformation Pitfalls: From Clinical Examination to Practical Imaging Evaluation--Part 1, Lymphatic Malformation Mimickers, AJR Am J Roentgenol, 940-51, May;206(5), 10.2214/AJR.15.15793
[5] Giarraputo L, Savastano S, D'Amore E, Baciliero U, (2018), Dermoid Cyst of the Floor of the Mouth: Diagnostic Imaging Findings, Cureus, e2403, pr 2;10(4), 10.7759/cureus.2403

### Besides Brain: A case report of an intra-osseous fibroma of the lower jaw in Tuberous Sclerosis Complex

*Authors:* O. Barut, S. Telli Erdoğan, C. Falk Kieri, E. Levring Jäghagen; Umeå/SE

**Learning objectives:** To increase knowledge of oral manifestations of Tuberous Sclerosis Complex (TSC) in the head and neck region and, to present defining imaging features of intra-osseous fibrous jaw pathologies.
**Background:** Tuberous Sclerosis Complex (TSC) is a rare condition (1/10000 newborn), often new mutation but sometimes inherited phacomatosis, characterized by multiple systemic manifestations. The diagnosis is based on a combination of clinical and genetic features. Central nervous system findings are common and play a crucial role in diagnosis. Although oral findings are not prerequisites, gingival fibromas are included in the diagnostic criteria as one of the minor features of the disease. Contrary to other more common oral findings like gingival fibromatosis or enamel pits, intra-osseous fibroma is a rare pathology.
**Imaging findings and procedure details:** A 17-year-old boy was referred to our hospital with vision impairment and severe headache. Papilledema and retinal bleeding were noted. A acute CT showed an intraventricular mass and hydrocephalus, and MRI revealed an intraventricular tumor and several subependymal hamartomas. Urgent brain surgery was performed, and a Subependymal Giant Cell Astrocytoma (SEGA) was histopathologically proven. Following clinical and genetic investigations, the patient was diagnosed with TSC with a mutation in the TSC-1 gene. He was referred to the department of Pediatric dentistry due to gingival changes. A panoramic radiographic examination of the jaws showed a radiolucency in the right mandible. Further examination with CBCT demonstrated a central expansive osteolytic lesion and MRI revealed a diffuse contrast enhancing mass with low signal intensity in both T1w and T2w sequences. As the mass was judged to represent benign features, total excision was performed. The following histopathological examination showed numerous islands of odontogenic epithelium, calcifications, and hyalinization. The lesion was diagnosed as odontogenic fibroma. A 6-month CBCT follow-up showed no recurrence.
**Conclusion:** In the literature there are very few cases describing intra-osseous fibroma and the association between odontogenic fibroma and TSC. However, TSC should be considered in patients with odontogenic fibroma, and it may be beneficial to include and evaluate the complete facial skeleton in the radiological examination of these patients in order to increase the chance for early detection and identification of TSC-related lesions.

### Creation of a semi-automated pipeline for parotid segmentation

*Authors:* S.W. Goh, J.L. Tan, K. Choy, A.Y.Q. Soon; Singapore/SG

**Learning objectives:** We aim to detail the steps to create a similar model using easy-to-use software for general radiologists, starting from raw Digital Imaging and Communications in Medicine (DICOM) files:
1. Anonymisation
2. Coregistration
3. Segmentation
Similar techniques can be applied elsewhere in the head & neck, for which there are limited publicly available segmentation models.
**Background:** There are a variety of parotid segmentation models trained from scratch with convolutional neural networks ([1],[2]). Recent advances enable the training of similar models with limited technical expertise required and provide good results with limited training data. This can turbocharge future research through the more rapid production of ground-truth labels.
**Imaging findings and procedure details:**
1. Anonymising DICOM
Open-source tools can provide precise control on which identifying tags to strip, which to replace (e.g. patient identifiers with study-level identifiers) and which to keep (e.g. sequence information), to anonymise data but keep needed technical information ([3]). These are called by batch files to provide automation for the multitude of DICOM files in the project.
2. Coregistration
Sequences must be properly coregistered for analysis to avoid contamination from surrounding tissues. Tools like SimpleITK ([4]) can help expediently register the desired sequences.
There may be a need to repackage the files into the Neuroimaging Informatics Technology Initiative (NIfTI) format, a popular format for imaging research. The whole sequence is stored in one file, with metadata in the header.
3. Segmentation
MONAI-label ([5]) is a free backend which simplifies the technical process of training; this was leveraged to train the model, with 3DSlicer as the frontend for the interactive segmentation. Interactive segmentation allows for progressive time savings as the model becomes more accurate with each round of training.
Two models were trained on 63 MRIs of parotid tumours – One of the parotids only (including the tumour, henceforth referred to as "parotid-only"), and another of the parotids with the tumour separated ("parotid+tumour"). A further 5 scans were used for validation.
The parotid-only model was trained for a total of 3350 epochs, and the parotid+tumour model for 2350 epochs.
4. Benchmarks
Training the parotid+tumour model took 14 hours on a single RTX 3070 TI.
The mean Dice coefficient of the parotids-only model (reported by MONAI) was 0.851, while that of the parotid+tumour model was 0.764. The mean Dice coefficient of the parotid-only model on the validation set was 0.991, while that of the parotid+tumour model was 0.778.

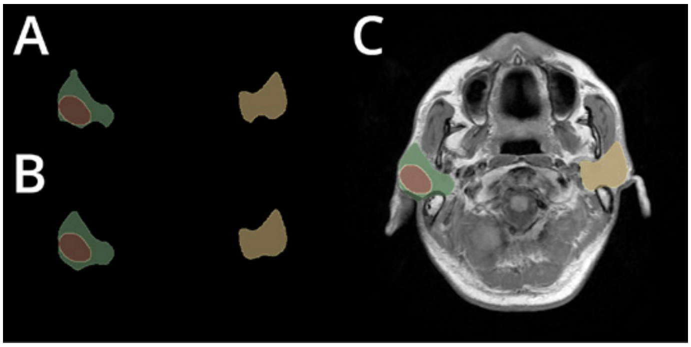

Example of one of the segmentation outputs (parotid+tumour). A. Predicted segmentation; B. Ground Truth segmentation; C. Predicted & Ground Truth overlaid on the original image. Non-overlapping areas are shown in darker colours.

**Conclusion:** This poster details a method for achieving coregistered, anonymised data for further use in parotid tumour analysis or for segmenting other organs in the head & neck,

References:
[1] Zi'an Xu, Yin Dai, Fayu Liu, Siqi Li, Sheng Liu, Lifu Shi, Jun Fu, (2022), Parotid Gland MRI Segmentation Based on Swin-Unet and Multimodal Images, arXiv, arXiv, Shenyang, -, -, https://arxiv.org/abs/2206.03336v2, 2024-03-24, China, Northeastern University
[2] Jennifer P. Kieselmann, Clifton D. Fuller, Oliver J. Gurney-Champion, Uwe Oelfke, (2020), Auto-segmentation of the parotid glands on MR images of head and neck cancer patients with deep learning strategies, medRxiv, medRxiv, Sutton, -, -, https://doi.org/10.1101/2020.12.19.20248376, 2024-03-24, United Kingdom, Joint Department of Physics, The Institute of Cancer Research and The Royal Marsden NHS
[3] DICOM Standards Committee, Working Group 18 Clinical Trials, (2011), Digital Imaging and Communications in Medicine (DICOM) Supplement 142: Clinical Trial De-identification Profiles, DICOM Standards Committee, -, Rosslyn, Virginia, -, -, https://www.dicomstandard.org/News-dir/ftsup/docs/sups/sup142.pdf, 2024-03-24, DICOM Standards Committee, DICOM Standards Committee
[4] Bradley C. Lowekamp, DavidT.Chen, Luis Ibáñez, Daniel Blezek, (2013), The Design of SimpleITK, Frontiers in Neuroinformatics, Frontiers in Neuroinformatics, Bethesda, Maryland, 1-14, Vol 7, Article 45, https://doi.org/10.3389/fninf.2013.00045, 2024-03-24, United States of America, National Library of Medicine, Office of High Performance Computing and Communications, National Inst
[5] Andres Diaz-Pinto, Sachidanand Alle, Vishwesh Nath, Yucheng Tang, Alvin Ihsani, Muhammad Asad, Fernando Pérez-García, Pritesh Mehta, Wenqi Li, Mona Flores, Holger R. Roth, Tom Vercauteren, Daguang Xu, Prerna Dogra, Sebastien Ourselin, Andrew Feng, M. Jorge Cardoso, (2022), MONAI Label: A framework for AI-assisted Interactive Labeling of 3D Medical Images, arXiv, arXiv, London, -, -, https://arxiv.org/abs/2203.12362, 2024-03-24, London, School of Biomedical Engineering & Imaging Sciences, King's College London

### CT staging of thyroid cancer: Common pitfalls and how to avoid them

*Authors: E. Mpiliouri, V. Alexiadou, E. Xinou, E. Sinduka, E. Karoglou; Thessaloniki/GR*

**Learning objectives:**
- Imaging findings regarding N and M staging of thyroid cancer on CT scan
- Imaging pitfalls in staging and how to avoid them

**Background:** Thyroid cancer (TC) is divided into 4 subtypes: papillary and follicular (well-differentiated - DTC), medullary (MTC) and anaplastic. According to ACR guidelines, CT and US are the modalities of choice for chest and neck staging of patients with high risk of recurrence. In most cases CT and US are both necessary and supplementary.
CT is performed preoperatively for surgical planning in patients with clinical suspicion of extrathyroidal extension and in the setting of MTC with high calcitonin levels. The indication for postsurgical CT is high tumor markers, depending on the histological subtype. Following total thyroidectomy, the use of iodinated contrast should be avoided if postoperative RAI therapy is considered within the next 3 months.
TC metastases have some characteristics which lead to staging pitfalls, regarding lymph nodes, lung and hepatic metastases.

**Imaging findings and procedure details:**
- TC metastatic lymph nodes are usually strongly enhancing and easily mistaken for vessels in axial planes, hence coronal reconstructions are mandatory in order to make the differential diagnosis.
- Small enhancing lymph nodes in patients with TC and high thyroglobulin or calcitonin, should be considered suspicious regardless of their size (Fig 1).
- Metastases to retropharyngeal lymph nodes are common not only when lower lymph node compartments are infiltrated or after lymph node dissection, but also as skip metastases.
- Calcifications can be encountered in MTC lymph nodes and can be misdiagnosed as granulomas. Calcifications are easily depicted in images without IV contrast (Fig 2).
- Metastatic lymph nodes can be subjected to cystic degeneration (Fig 2).
- DTC lung metastases are usually multiple and subcentimeter in size, therefore MIP (maximum intensity projection) visualisation is necessary in order to detect tiny lung metastases.
- MTC metastases (usually lung and liver) can have calcifications and can be misdiagnosed as granulomas or inflammatory lesions (Fig 2,3).
- TC hepatic metastases are often misdiagnosed as hemangiomas, because of their arterial enhancement. For the characterisation of a lesion as hemangioma we should compare the lesion to the blood pool: hemangiomas always follow the blood pool, in contrast to TC hepatic metastases (Fig 4).

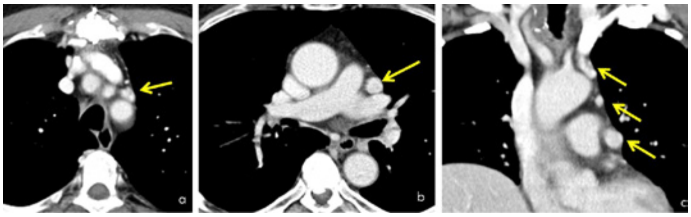

Figure 1: Enhancing metastatic lymph nodes: a,b) In axial planes enhancing lymph nodes can be mistaken for blood vessels. c) In coronal views most vessels are longitudinal and differential diagnosis is easier.

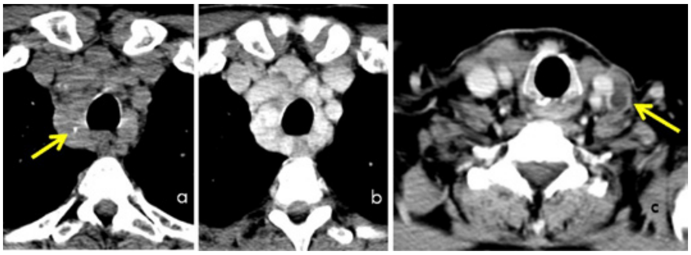

Figure 2. Metastatic lymph nodes: a,b) Metastatic lymph nodes, especially from MTC can appear with calcifications, which are depicted without IV contrast. c) Cystic degeneration of metastatic lymph nodes can be encountered regardless of their size.

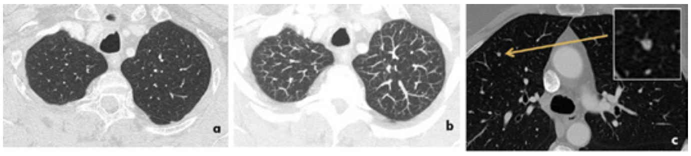

Figure 3. Lung metastases: a) Lung metastases are usually subcentimeter in size and multiple, and therefore they are very difficult to detect in thin 1mm slices with lung window. b) Using MIP in the same patient as a) multiple lung nodules are visualised. c) Microcalcifications in metastatic lung nodules can be encountered in MTC.

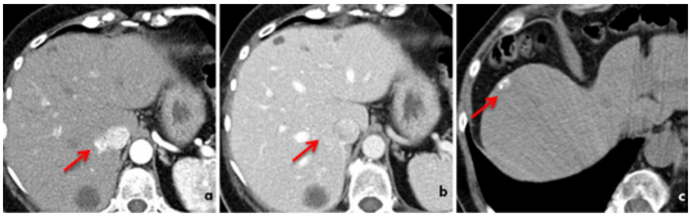

Figure 4. Hepatic metastases: TC hepatic metastasis showing strong enhancement in arterial phase (a) and isointensity in venous phase (b). The enhancement pattern is different from the blood pool, and therefore, the diagnosis of hemangioma can be excluded. c) Images without contrast are necessary to detect calcifications in MTC liver metastases.

**Conclusion:** Improving basic knowledge and determining typical imaging findings of TC metastatic disease through a systematic approach of CT scans may help in avoiding pitfalls and improve the diagnostic accuracy of CT staging.

### Demystifying Segmentation and Data Extraction: A Step-by-Step Guide for Initiating a Scientific Project on Oropharyngeal Cancer Images

*Authors: S. Medrano-Martorell[1], M. Fresno-Vallespir[1], Y. Rodríguez-Álvarez[1], A. Massuet-Vilamajor[1], D. Choque-Chávez[1], A. Jareño-Badenas[1], S. González-Ortiz[1], C. Pineda-Ibarra[1], A. Pérez-Lara[2], L. Oleaga-Zufiría[1]; [1]Barcelona/ES, [2]Málaga/ES*

**Learning objectives:**
- To be able to identify opportunities for the development of a scientific project based on image data extraction, considering the image characteristics and the field of study.
- To demonstrate the steps involved in building a database from image segmentation.
- To illustrate the process with the example of a cohort of patients with oropharyngeal cancer.

**Background:** Technological advances in imaging, with the advent of radiomics and artificial intelligence, make it necessary to integrate advanced image management and processing workflows into the educational objectives of radiology residency.

**Imaging findings and procedure details:** This work illustrates the steps involved in the process of image segmentation, radiomics data extraction, and database configuration, using an example of CT images from a cohort of patients with oropharyngeal cancer from our center.

**Conclusion:** This work aims to familiarize radiologists of head and neck with the tasks of image data extraction for further analysis, as well as to provide a method that encourages the development of scientific research in the era of the artificial intelligence revolution.

### Differential diagnosis and radiotherapy treatment of different types of radio-induced malignancies of the head and neck region: Our experience at the National Center for Oncological Hadrontherapy (CNAO) in Pavia, Italy

*Authors:* *M. Pecorilla, S. Imparato, M.F. Achilli, C. Fichera, A. Chalaszczyk, M. Rotondi, M.R. Fiore, E. Orlandi, L. Preda; Pavia/IT*

**Learning objectives:** To provide an overview of radio-induced neoplasms in the patient population undergoing radiotherapy in the head and neck district, through our experience at the Center National Center for Oncology Hadrontherapy (CNAO) in Pavia, comparing the characteristics of signal in magnetic resonance imaging (MRI) in order to support differential diagnosis, and illustrating the hadrontherapy treatment performed with protons or carbon ions.

**Background:** We reviewed 6 cases of radio-induced tumors treated at our center from 2014 to 2023. Clinical data were collected on primary diagnosis, radiotherapy history and latency period. We examined MRI images with contrast acquired before and after treatment and CT scan without contrast acquired before treatment.

The type of hadrontherapy particles used and the radiation dose were specified.

The histological types of the 6 selected cases were chondroblastic osteosarcoma, spindle cell sarcoma, schwannoma, squamous cell carcinoma, meningioma and leiomyosarcoma. The patients included were 4 women and 2 men, with a mean age at diagnosis of 35 years (range 21-55 years) and mean latency period for neoplasm development of 21.84 years (range 4-45 years).

| CASES | YEAR OF RT TREATMENT | RT DOSE TREATMENT (Gy) | AGE AT DIAGNOSIS OF RIT | YEAR OF ONSET RIT | LATENCY PERIOD |
|---|---|---|---|---|---|
| Chondroblastic osteosarcoma | 1998 | 100 | 30 | 2013 | 15 |
| Spindle cell sarcoma | 2012 | 59 | 27 | 2016 | 4 |
| Schwannoma | 2012 | 167 | 21 | 2016 | 4 |
| Squamous cell carcinoma | 1989 | 180 | 55 | 2021 | 32 |
| Meningioma | 1964 | 12 | 46 | 2009 | 45 |
| Leiomyosarco ma | 1990 | 50 | 31 | 2022 | 31 |
| | | mean RT dose: 95 Gy | mean age: 35 years | | mean latency period: 21.83 years |

Time of onset of radio-induced malignancies of the head and neck

**Imaging findings and procedure details:** We performed MRI examinations with a 3T system (Skyrafit - Siemens) analyzing the signal in T1- and T2-weighted sequences, DWI features, and contrast enhancement of lesions. Sequences acquired in the axial and coronal planes had slice thickness of 2 or 3 mm with distance factor of 0-10%.

Analysis of MRI and CT images showed for each case different signal characteristics, specific for each histotype and overlapping with those described in the literature.

The mean dose of radiotherapy treatments performed on the primary tumors was 95 Gy. The radiation treatment was conducted with carbon ions (50%) or protons (50%).

| CASES | T1w | T2w | FS | DWI Restriction | CE | Non-Contrast CT |
|---|---|---|---|---|---|---|
| Chondroblastic osteosarcoma | iso/hypointense | hypo/slightly hyperintense | no | yes | inhomogeneous, predominantly peripheral | isodense, with huge calcifications |
| Spindle cell sarcoma | isointense | slightly hyperintense | no | yes | inhomogeneous, predominantly peripheral | isodense |
| Schwannoma | iso/hypointense | hypointense centrale core, hyperintense peripheral | no | slightly yes | salt and pepper | iso/hypodense |
| Squamous cell carcinoma | iso/hypointense | iso/hypointense | no | yes | inhomogeneous | isodense |
| Meningioma | iso/hyperintense | iso/slightly hyperintense | no | slightly yes | inhomogeneous | isodense |
| Leiomyosarcoma | isointense | iso/hypointense | / | yes | inhomogeneous | / |

MR and CT features of radio-induced malignancies of the head and neck

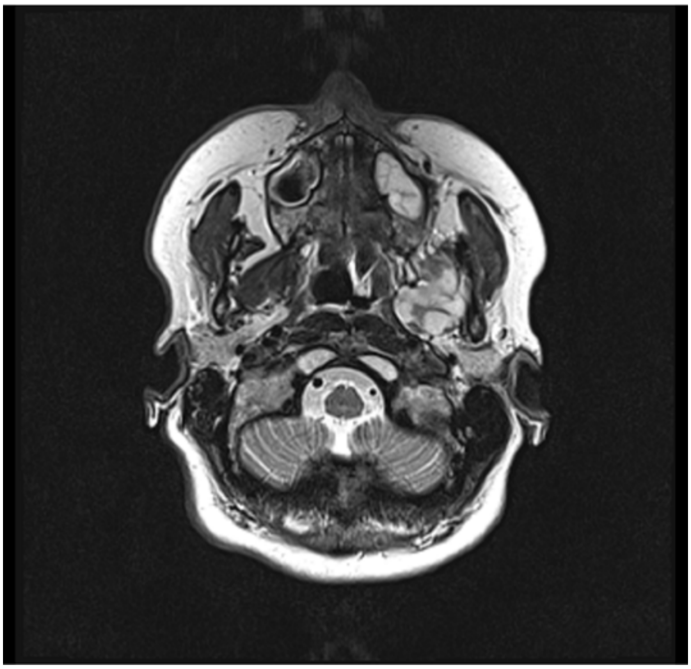

T2w section of radio-induced schwannoma of the left trigeminal nerve (case 3)

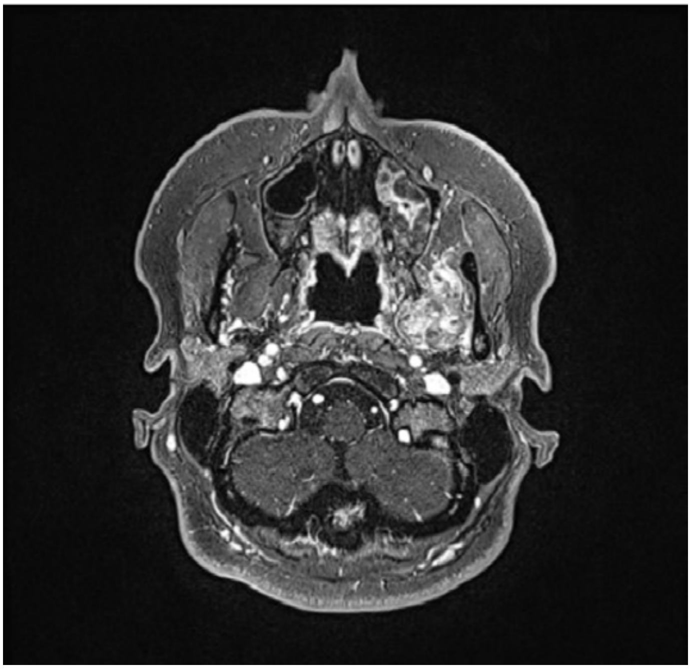

T1w post contrast agent section of radio-induced schwannoma of the left trigeminal nerve (case 3)

**Conclusion:** Radio-induced neoplasms are a rare serious complication of radiotherapy treatments of the head and neck region. Knowledge of imaging features, which are different and specific for each histologic type of radio-induced neoplasm, is essential to guide diagnosis and help define the treatment course.

### Facial Aesthetic Injections: What a Radiologist Needs to Know

*Authors: A. Nowelli, T. Nowicki, K. Markiet, E. Szurowska; Gdansk/PL*

**Learning objectives:**
- To recognize the significance of identifying aesthetic medicine procedures in computed tomography (CT) and magnetic resonance imaging (MRI).
- To understand the diversity of substances used in esthetic procedures and their imaging characteristics.
- To learn about the evolving characteristics of injected substances over time, their effects on tissues, and their distinct patterns of placement in facial anatomy.

**Background:** The demand for aesthetic medicine procedures, including facial injections, has surged dramatically in recent years. While hyaluronic acid injections have been already well-characterized in MRI, numerous other substances, such as calcium hydroxyapatite and poly-L-lactic acid (PLLA), are increasingly used for facial augmentation, but lack recognition in literature. Many of these procedures are performed by non-physicians increasing the risk of complications requiring medical intervention. Radiologists play a crucial role in diagnosing complications and guiding patient care, necessitating familiarity with the imaging characteristics of various aesthetic substances.
**Imaging findings and procedure details:** In CT and MRI, aesthetic substances exhibit diverse appearances and behaviours. The characteristics of these substances evolve over time post-injection, impacting tissue response and distribution within facial structures. This section will provide detailed insights into the imaging features of various fillers and substances used in aesthetic medicine procedures. Examples from clinical cases illustrate the nuances in appearance and distribution, aiding radiologists in accurate diagnosis and management of complications.
**Conclusion:** This poster enhances radiologists' proficiency in identifying aesthetic injections and related complications. Increased awareness among healthcare providers is essential to address challenges and to ensure optimal patient outcomes.

### Imaging in congenital sensorineural hearing loss: Radiologist's perspective from Cochlear implantation

*Authors: M.S. Swarup, M. Jain, K. Gehlot; New Delhi/IN*

**Learning objectives:**
1. To discuss the role of HRCT and MR imaging in patients presenting with congenital sensorineural hearing loss
2. To review imaging fifindings of various forms of congenital inner ear malformation using a case-based approach
3. To discuss the role of radiologists in guiding surgeons in planning cochlear implantation
4. To provide a detailed reporting checklist for generating a clinically relevant report for proper management

**Background:** VCongenital sensorineural hearing loss may result from structural or functional abnormalities in the inner ear, vestibulo-cochlear nerve, or auditory processing centers of brain. The abnormality may be genetic or sequela of perinatal insult; and in many cases, it is idiopathic. Cross sectional imaging plays a central role in evaluation of childrens presenting with congenital sensory neural hearing loss. HRCT temporal bone and MRI of Inner ear-IAC and brain are complimentary to each other in comprehensive evaluation of these patients.
**Imaging findings and procedure details:** We assessed all the imaging studies done in our department in patients presented with congenital sensorineural hearing loss and were evaluated as possible cochlear implantation candidates. HRCT temporal bone is the main workhorse in evaluating osseous labyrinth, bony facial canal, cochlear aperture and mastoid pneumatization, identifying difficulties while cochlear implantation with features of small mastoid cavity, anteriorly displaced facial nerve and/or decreased facial recess width. MRI inner ear particularly 3D-T2 sequences (CUBE/ FIESTA) helpful in evaluating membraneous labyrinthine abnormalities and vestibulo-cochlear nerve.
The following types of congenital inner ear malformations will be discussed using a multi-modality imaging approach (HRCT and MRI) highlighting important differentiating points. Various anatomical variations and disease processes of the mastoid, middle ear and inner ear that have an impact on cochlear implant surgery will be discussed briefly from a radiologist's perspective.
- Complete labyrinthine aplasia
- Cochlear aplasia
- Common cavity malformation
- Incomplete partition type 1, type 2, type 3
- Cochlear hypoplasia
- Dilated vestibular aqueduct
- Vestibular and semicircular canal anomalies
- Vestibulocochlear nerve (VCN) anomalies - VCN aplasia, hypoplasia
- Internal auditory canal (IAC) and cochlear aperture abnormalities

**Conclusion:** Familiarity with imaging findings of various congenital inner ear malformations and differentiating points for correct diagnosis is imperative for formulating a management strategy and deciding indication for cochlear implantation. Providing a combined HRCT temporal bone and MRI inner ear report with a proper checklist and mention of all surgically relevant findings will help surgeons to correctly plan cochlear implantation.

### Imaging in Deep Neck Abscesses: Radiologic Anatomy of Spaces and Case Examples

*Authors: Y. Gül, G. Kılıçarslan; Elazıg/TR*

**Learning objectives:**
- Learning about neck fascia and its spaces.
- Understanding the extension of deep neck abscesses into spaces and the importance of imaging.

**Background:** Deep neck abscesses occur in spaces formed by layers of deep cervical fascia. Most commonly seen in peritonsillar, parapharyngeal localizations. It is more common in men, young age and diabetic elderly patients. Mostly odontogenic origin (50-70%). It can progress rapidly to airway obstruction, venous thrombosis, arterial erosion, mediastinitis and sepsis, causing significant mortality and morbidity. Anatomy of the neck space is useful in assessing the spread of abscesses. The SDCF adheres anteriorly to the hyoid, superiorly to the mandible, mastoid process and external occipital protuberance. The MLDCF extends from the skull base to the mediastinum. It is called the buccopharyngeal fascia posteriorly at the suprahyoid level. The DDCF has two components as prevertebral and alar fascia. The carotid sheath is formed by three layers of the deep cervical fascia.
Spaces are grouped according to the hyoid bone (Tablo 3, Figure 1,2,3). Knowledge of the tissues in the spacings will be of great benefit to us radiologists in the differential diagnosis of pathologies that may develop.

| Suprahyoid spaces (Figure 1) | Supra-infrahyoid spaces (Figure 2) | İnfrahyoid spaces (Figure 3) |
|---|---|---|
| Parapharyngeal space | Carotid space (poststyloid parapharyngeal space) | Visceral space |
| Pharyngeal mucosal space | Retropharyngeal/ retroesophageal space | Posterior cervical space |
| Parotid space | Danger space | |
| Masticator space | Perivertebral space (prevertebral and paravertebral) | |
| Buccal space | | |
| Sublingual space | | |
| Submandibular space | | |
| Submental space | | |

Table 3: Suprahyoid, infrahyoid and spaces extending to both regions.

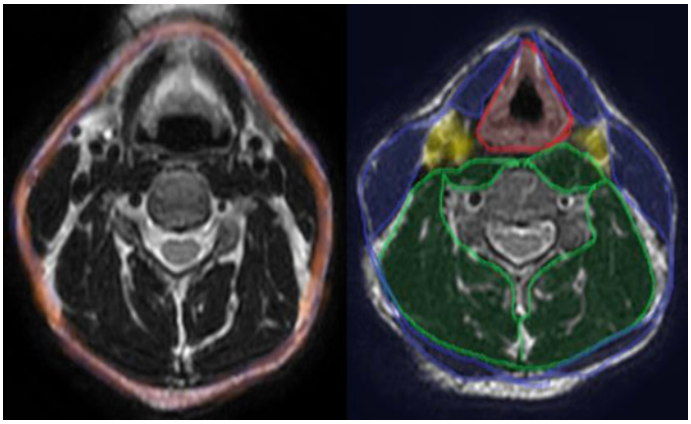

Figure 1. Radiologic images of the cervical faciae

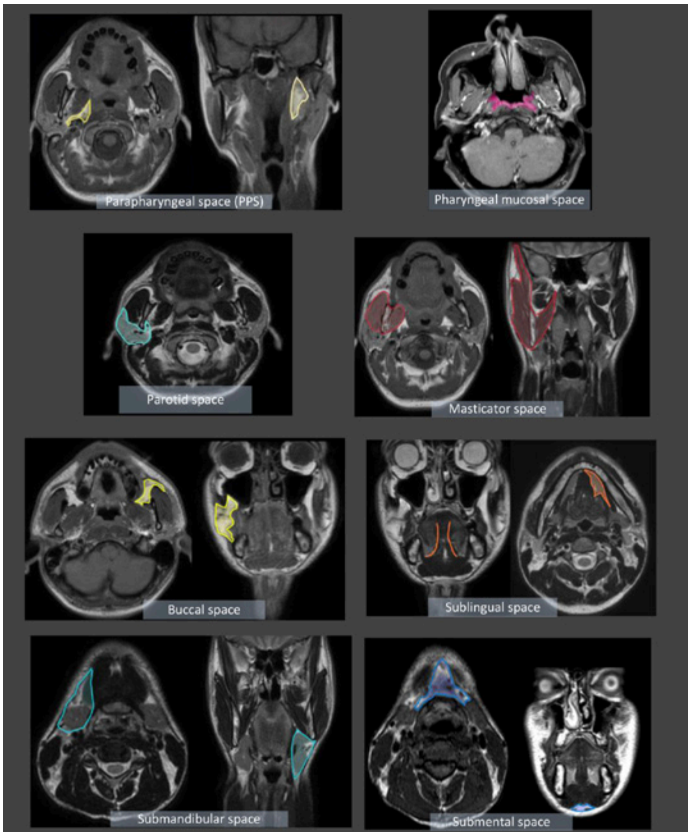

Figure 2. The suprahyoid spaces

**Imaging findings and procedure details:** Contrast-enhanced computed tomography (CT) plays an important role in the diagnosis. The size of the lesion, extension and possible complications should be evaluated. MR imaging shows soft tissues better and possible intracranial spread. MRI shows abscess, intermediate-hypointense on T1A and hyperintense on T2A, peripheral contrast enhancement on contrast-enhanced T1A, and diffusion restriction on ADC. Retropharyngeal collections that have entered the danger or prevertebral spaces can descend to the mediastinum and as far inferiorly as the coccyx. Retrofaringeal space ve danger space görüntülemede ayırtedilemez. Asymmetric appearance at the level of nasopharynx should suggest retropharyngeal abscess and unilocular appearance should suggest danger space involvement.

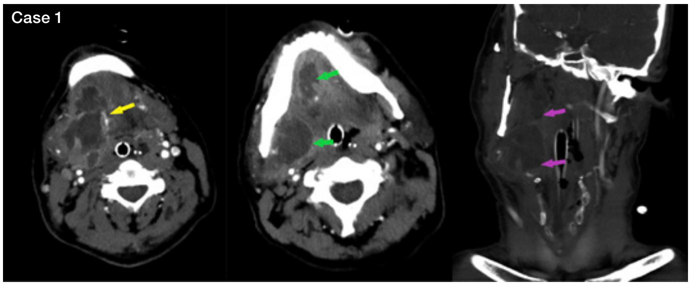

40 Y, M, A patient with pansinusitis and diabetes mellitus developed an abscess after tooth extraction. Contrast enhanced CT scan of the neck showing spread to sublingual space (yellow arrow), submandibular space (green arrow), parapharyngeal space (pink arrow)

**Conclusion:** An anatomy-oriented approach to deep neck spaces facilitates the process and improves diagnostic accuracy. It allows the radiologist to better assess possible routes of spread, understand clinical risks and identify possible causes.

**Keywords:** Servikal facia, space, deep neck abcess

References:
[1] Juerg Hodler · Rahel A. Kubik-Huch · Gustav K. von Schulthess, (2020), Head and Neck, Spine 2020–2023, IDKD Springer Series, https://doi.org/10.1007/978-3-030-38490-6_16
[2] Blake W, Hansen MD, Stanislav Ryndin DO, Katherine M. Mullen MD, (2019), Infections of Deep Neck Spaces, Seminars in Ultrasound CT and MRI , doi: https://doi.org/10.1053/j.sult.2019.10.001
[3] Bing W, Bu-Lang G, Guo-Ping X and Cheng X, (2013), Images of deep neck space infection and the clinical significance, Acta Radiol, https://doi.org/10.1177/0284185113509093

### Imaging of high-flow vascular lesions in the head and neck region

*Authors:* X.Y. Tan, G.W. Tan, L. McAdory, S. Guo, C.L. Ho; Singapore/SG

**Learning objectives:** To review imaging features of non-traumatic high-flow vascular lesions in the head and neck encountered in our institutions.
**Background:** According to Mulliken and Glowacki, vascular malformations are classified based on their flow characteristics into slow and high-flow types[1]. High-flow vascular lesions in the head and neck often present diagnostic and therapeutic challenges, due to unfamiliarity of their imaging features and clinical course as well as paucity of cases in routine radiology practice.
**Imaging findings and procedure details:** In retrospective review of head and neck high-flow vascular lesions from two tertiary hospitals in Singapore, we have identified the following conditions:

1. Carotid blowout post chemo-radiotherapy of nasopharyngeal cancer
2. Bilateral dissecting pseudoaneurysms of the internal carotid arteries
3. Carotid pseudoaneurysm from persistent hypoglossal artery
4. Pinna and peri-auricular arteriovenous malformation
5. Falcotentorial arteriovenous fistula
6. Vein of Galen dural arteriovenous fistula
7. Occipital scalp pseudoaneurysm

Complications related to these high-flow vascular lesions can include life-threatening intra- and extra-cranial haemorrhages and debilitating stroke. It is therefore crucial to promptly identify these lesions with multimodality imaging including magnetic resonance imaging (MRI), computed tomography (CT) and digital subtraction angiography (DSA). Timely management include endovascular and/or surgical treatment, essential to prevent patient deterioration.

Brief example:

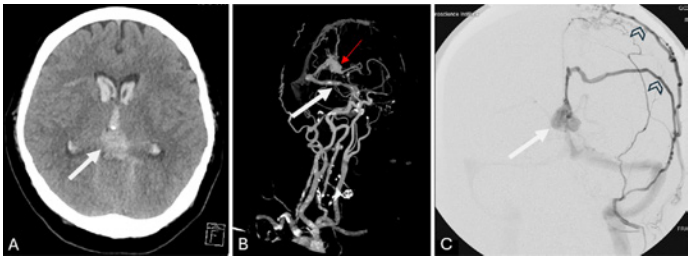

A 61-year-old female presented with vertiginous giddiness and drowsiness. (A) Non-contrast CT brain shows extensive subarachnoid and intraventricular haemorrhages and hyperdensity at the confluence of the deep cerebral veins (arrow). (B) CT Circle of Willis/delayed CT cerebral venogram demonstrate an aneurysmal dilation of the vein of Galen (red-arrow) with arteriovenous fistula involving tributaries from both MCAs and PCAs. 3D rotational angiogram shows the malformation preferentially draining into the straight sinus and left transverse sinus (arrow). (C) A-P projection of DSA during angioembolisation confirms the vein of Galen malformation (arrow) with additional feeders from bilateral ICA and ECA (arrowheads).
**Conclusion:** High-flow vascular lesions of the head and neck can be associated with life-threatening complications and require prompt recognition with multimodality imaging and timely management.

References:
[1] Mulliken JB, Glowacki J. Hemangiomas and vascular malformations in infants and children: a classification based on endothelial characteristics. Plast Reconstr Surg. 1982;69:412-22. doi: 10.1097/00006534-198203000-00002.

### Imaging of isolated congenital middle ear malformations

*Authors:* K. Bergaoui, A. Ntorkou, M. Alison, M. Elmaleh-Bergès; Paris/FR

**Learning objectives:**
- Understand the diagnostic challenges associated with isolated congenital middle ear malformations (CMEM).
- Accurately describe the anatomical anomalies encountered in CMEM.
- Recognize key imaging findings indicative of different malformation subtypes, based on an adaptation of the Teunissen Classification.

**Background:** Isolated CMEM, i.e., without associated abnormalities of the external ear or the tympanic membrane, poses diagnostic challenges for clinicians and pediatric radiologists. Accurate characterization remains essential for effective clinical management
**Imaging findings and procedure details:** Congenital isolated middle ear malformations encompass a spectrum of anatomical variations, ranging from subtle anomalies to complex deformities. The Teunissen classification system, based on surgical findings, provides a structured framework for categorizing these malformations according to their anatomical features and can be utilized for preoperative imaging. In this context, high-resolution computed tomography (HRCT) plays a pivotal role.
Stapes malformations, the most common in isolated middle ear deformities, often involve combined deformities of the stapes suprastructure and incus. Incudostapedial joint fusions, stapes aplasia or hypoplasia, and stapes fixation due to bony plates or ligament dysplasia may occur. Isolated stapes fixation (Teunissen class I) is difficult to assess in preoperative imaging.
Incus malformations often involve absence or hypoplasia of the long process, along with separation of the incudostapedial joint. Variations in position, such as horizontal rotation, and complete aplasia of the incus are less frequent. Synostotic or synchondrotic incudomalleolar joint abnormalities and epitympanic recess fixation are also observed. The malleus, the least affected ossicle, shows minimal involvement in middle ear malformations. Common findings include deformities, hypoplasia of its head and manubrium, fixations in the epitympanic recess, and abnormalities in the malleoloincudal joint. Malleus and/or incus anomalies are characteristic of Teunissen Class II and III CMEMs. Oval or round window involvement may manifest as aplasia or severe dysplasia. Additionally, in this case, aberrant facial nerves, including dehiscence or inferior displacement of the tympanic segment, are commonly observed. These anomalies are characteristic of Class IV Teunissen CMEM.
**Conclusion:** Accurate diagnosis of isolated congenital middle ear malformations (CMEM) presents challenges that demand precise characterization through HRCT imaging. Preoperative assessment can suggest the malformation subtype based on a the Teunissen Classification.

### Imaging Spectrum of Trigeminal Nerve Pathology

*Authors:* *J. Lee[1,2], M. Lee[2,3], R. Wiggins[2]; [1]Victoria/AU, [2]Salt Lake City/US, [3]Singapore/SG*

**Learning objectives:**
- Review common and rare pathologies of the trigeminal nerve
- Understand anatomical course of trigeminal nerve and its branches, and adjacent structures, to inform imaging approach
- Recognize typical imaging features on both CT and MRI imaging to guide differential diagnosis and management.
- Highlight imaging pitfalls in the imaging of the pathology of trigeminal nerves.

**Background:** Due to its extensive sensory innervation to the face and motor innervation to the muscles of mastication[1], the trigeminal nerve is commonly affected by a variety of benign and malignant pathologies. Imaging is critical to assess its pathology and to guide management.
We will review common and rare pathologies of the trigeminal nerve and consider its anatomical course and relationship to adjacent structures to inform imaging approach. Typical imaging features on both CT and MRI imaging will also be discussed to guide differential diagnosis and management.

**Imaging findings and procedure details:** Benign pathologies of the trigeminal nerve can be split into pathologies that directly involve the nerve and secondary pathologies from adjacent structures. Common benign pathologies that directly involve the trigeminal nerve include nerve sheath tumors, such as neurofibromas and schwannomas, and inflammatory conditions such as IgG4 disease and sarcoidosis. Imaging features to differentiate between these pathologies will be discussed, such as target sign[2].

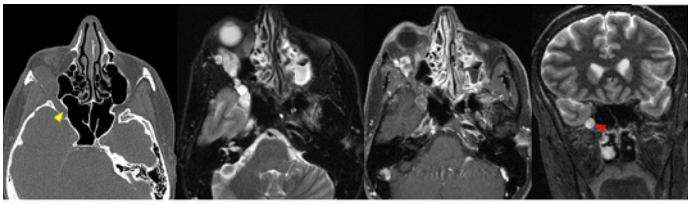

Right biopsy-proven neurofibroma, thinning the adjacent bone and widening the inferior orbital fissure (yellow arrowhead). On MRI imaging, it returns high T2 signal, minimal enhancement and target sign (red arrowhead), more typical of neurofibromas compared with schwannomas[3].

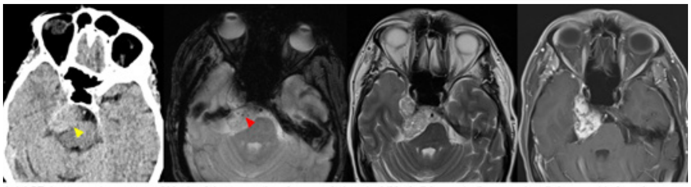

Axial CT demonstrating a mass within the right pre-pontine cistern and trigeminal (Meckel's) cave (yellow arrowhead), inseparable from the trigeminal nerve. The mass demonstrates minimally increased density (yellow arrowhead), suggesting a degree of haemorrhage, which was confirmed on MRI imaging, with foci of low signal on gradient-weighted imaging (red arrowhead). The mass returns heterogenous, predominantly intermediate T2 signal, with small foci of high T2 signal, and enhancement.

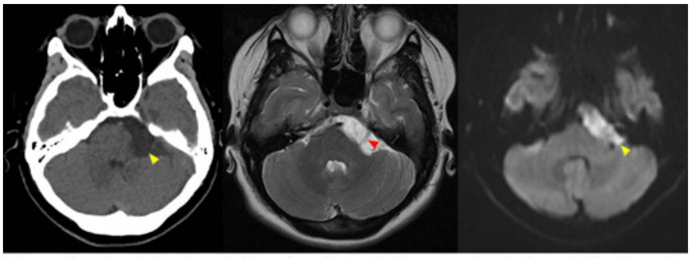

Left pre-pontine and cerebellopontine angle cistern epidermoid cyst, which demonstrates hypodensity on axial CT and abnormal restricted diffusion on axial MRI images (yellow arrowhead). The epidermoid cyst encases and displaces the left cisternal trigeminal nerve (red arrowhead).

Secondary benign pathologies of the trigeminal nerve include compression from adjacent pathologies or structures, such as meningioma, neurovascular compression[3] or cysts within the pre-pontine cistern, including epidermoid cysts.
The trigeminal nerve can also be involved by primary or secondary malignancies, including perineural spread[4]. Typical imaging features and imaging pitfalls will also be discussed.

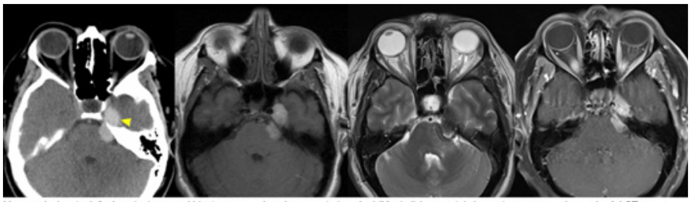

Mass replacing the left trigeminal nerve within the pre-pontine cistern and trigeminal (Meckel's) cave. It is hyperdense on unenhanced axial CT scan (yellow arrowhead). On MRI imaging, it returns high T1 (left) and low T2 (right) signal, suggesting melanocytic or haemorrhagic contents. The mass returns homogenous enhancement (right). Biopsy revealed melanoma.

**Conclusion:** Understanding imaging features and considerations when imaging the trigeminal nerve will allow the radiologist to improve our diagnosis of trigeminal nerve pathologies to guide management.

References:
[1] Romano N, Federici M, Castaldi A. , (2019), Imaging of cranial nerves: a pictorial overview, Insights Imaging, 33, 10(1), https://doi.org/10.1186/s13244-019-0719-5
[2] Haller S, Etienne L, Kövari E, Varoquaux AD, Urbach H, Becker M, (2016), Imaging of Neurovascular Compression Syndromes: Trigeminal Neuralgia, Hemifacial Spasm, Vestibular Paroxysmia, and Glossopharyngeal Neuralgia. , Am J Neuroradiol, 1384-92, 37(8), https://doi.org/10.3174/ajnr.A4683
[3] Banks KP, (2005), The target sign: extremity. , Radiology, 899-900, 234(3), https://doi.org/10.1148/radiol.2343030946
[4] Dankbaar JW, Pameijer FA, Hendrikse J, Schmalfuss IM. , (2018), Easily detected signs of perineural tumour spread in head and neck cancer., Insights Imaging, 1089-1095, 9(6), https://doi.org/10.1007/s13244-018-0672-8

### Imaging update of extranodal glandular involvement of Lymphoma in Head and Neck: The Parotid, Thyroid and Lacrimal

*Authors:* *M.S. Swarup, U. Singh, S. Sree V S, G. Sindhwani, R. Gupta Ghasi; New Delhi/IN*

**Learning objectives:**
1. To illustrate the imaging features of extranodal Head and Neck lymphomas involving glandular sites like Parotid, Thyroid and Lacrimal glands on different imaging modalities (Ultrasound, CECT, MRI, 18F-FDG PET /CECT)
2. To discuss the role of imaging in the diagnosis and evaluation of extranodal extra-lymphatic Head and Neck lymphoma
3. To highlight the role of ultrasound Elastography, Dynamic CT perfusion imaging, Dynamic MR perfusion and diffusion-weighted imaging in the diagnosis of lymphomatous involvement in these Head and Neck subsites and differentiation from other common glandular neoplasms with a brief discussion on possible differentiating points in imaging

**Background:** Extra-nodal lymphoma refers to lymphoma originating from soft tissues other than the lymph nodes. These constitute a minority of all malignant neoplasms in the head and neck. However, the head and neck region is the second most common site for extra-nodal lymphomas after the gastrointestinal tract. Most cases are non-Hodgkin's lymphomas (NHL) of B-cell lineage, with diffuse large B-cell lymphoma (DLBCL) being the predominant type. Variable imaging appearances across different extranodal anatomical sites in the head and neck, specifically in the salivary, thyroid and lacrimal glands pose a significant task for radiologists in establishing diagnosis and formulating differential diagnosis. Lymphomatous involvement of salivary/ parotid, thyroid and lacrimal glands is relatively uncommon compared to other neoplasms in these glands. Imaging morphology may be nonspecific and Ultrasound-guided biopsy remains the best method to obtain a definitive diagnosis.

**Imaging findings and procedure details:** The lymphomatous glandular involvement can occur in the diffuse form (diffuse enlargement of the gland without focal nodules), or the focal form (as a focal nodule on a background of Hashimoto thyroiditis in the thyroid or focal mass in the parotid gland). The nodule/ mass may be completely solid or mixed solid cystic and can be either solitary or multifocal.

A multimodality imaging review of extranodal lymphomatous glandular involvement will be provided using the available imaging modalities ( Ultrasound, CECT, CEMRI, 18F-FDG PET /CECT)
1. Three cases of lymphomatous involvement of the parotid gland (Hodgkin's and Non-Hodgkin's lymphoma) - Primary and secondary
2. Three cases of lymphomatous involvement of thyroid gland (Non-Hodgkin's lymphoma)
3. Two cases of orbital lymphoma with involvement of lacrimal gland

**Conclusion:** CT, MRI, and PET/CT play a crucial role in the diagnosis and accurate staging of Head and Neck lymphomas, which is essential for making management decisions and treatment planning. A radiologist's familiarity with these appearances facilitates early diagnosis and targeted oncological treatment.

### Lemierre syndrome, A comeback story

*Authors:* *A. Péporté[1], A. Venkatasamy[2]; [1]Frauenfeld/CH, [2]Strasbourg/FR*

**Learning objectives:**
This educational poster aims:
a.) To familiarize junior trainees/residents with the imaging patterns of Lemierre syndrome (LS).
b.) To highlight diagnostic pearls and potential pitfalls in the radiological diagnosis of LS.
c.) To describe a checklist of certain crucial anatomical sites, which must be carefully assessed when reporting cases of LS.

**Background:** LS is a severe infection, caused by the anaerobic bacterium Fusobacterium necrophorum[4]. Typically affecting otherwise healthy teenagers and young adults, the infection originates in the throat, and progresses to a septic thrombophlebitis of the tonsillar and internal jugular veins. Subsequent bacteraemia is exacerbated by the presence of septic emboli in various locations, including the lung, joints, and bones[1][2]. Despite its rarity, there has been evidence of a resurgence in recent years, possibly due to reduced antibiotic use for sore throats[3]. The clinical presentation is distinctive, yet many clinicians remain unfamiliar with the condition, leading to frequent diagnostic delays and potentially fatal outcomes.

**Imaging findings and procedure details:** Contrast enhanced CT is considered the imaging modality of choice due to its widespread availability and its ability to visualize complications and underlying infections effectively. MRI is typically reserved for cases where diagnosis remains unclear.

Both modalities can provide a high level of confidence in diagnosis:
a.) showing an intraluminal filling defect(s) in the jugular venous wall, often better visualized on CT compared to ultrasound, particularly in assessing deeper venous segments.
b.) demonstrating complications, such as dural venous sinus thrombosis, which are often better visualized by CT than ultrasound, particularly in assessing deeper venous segments.
c.) identifying sites of septic emboli, most often encountered as pulmonary septic emboli, more readily visualized by CT.
d.) often depicting the primary site of infection[1][3][4].

Potential alternative diagnoses, such as isolated oropharyngeal infection, isolated jugular vein thrombosis, cervical lymphadenitis, pneumonia, metastatic disease, or primary neoplasms should be considered and ruled out.

**Conclusion:** Radiological findings (such as internal jugular vein thrombophlebitis) often provide the first diagnostic clue, before positive bacterial cultures confirm the diagnosis. A high degree of suspicion together with a relevant clinical context is crucial for a timely diagnosis. Since there has been a noticeable rise in the number of reported cases, possibly due to inappropriate antibiotic use, radiologists should be familiar with this entity and typical imaging features.

References:
[1] Mitchell R Gore, (2020), Lemierre Syndrome: A Meta-analysis, Int Arch Otorhinolaryngol, e379-e385, 24(3), https://pubmed.ncbi.nlm.nih.gov/32754251/
[2] Carius BM, Koyfman A, Long B, (2022), High risk and low prevalence diseases: Lemierre's syndrome, 98-104, 61, https://pubmed.ncbi.nlm.nih.gov/36058204/
[3] Lee WS, Jean SS, Chen FL, Hsieh SM, Hsueh PR, (2020), Lemierre's syndrome: A forgotten and re-emerging infection, J Microbiol Immunol Infect, 513-517, 53(4), https://pubmed.ncbi.nlm.nih.gov/32303484/
[4] Kuppalli K, Livorsi D, Talati NJ, Osborn M, (2012), Lemierre's syndrome due to Fusobacterium necrophorum, Lancet Infect Dis, 808-15, 12(10), https://pubmed.ncbi.nlm.nih.gov/22633566/

### LOOK OUT: Orbital Emergencies. An interesting case series of pathology not to be overlooked

*Authors:* *L. Bell, M. Maviki, M. Tomek; Plymouth/GB*

**Learning objectives:**
- Present a collection of interesting orbital emergency cases from a busy neurosurgical and trauma centre.
- Highlight review areas and vital orbital structures.
- Helpful tips for reformatting.
- Suggest helpful protocols.
- Improve confidence and efficiency in assessing the orbits on CT and MR imaging.

**Background:** Orbits are imaged across an array of studies (trauma, neurological SOL, stroke imaging, neck imaging ) and can often be under scrutinised if the study is not specific for orbital pathology. This is seen especially in the setting of trauma; orbital pathology can be under reported with the focus of bony injuries on the standard trauma CT. This poster will guide the radiologist through important review areas via a collection of interesting cases.

**Imaging findings and procedure details:**
pictorial review to include: globe rupture, wood foreign body, muscle herniation, superior blowout injury

**Conclusion:** Orbits are an interesting and vital review area for any study in which they are included.
Radiologists (Specialist and General) should approach the orbits systematically to avoids missed or under estimation of pathology.

### Ménière's Disease: What to say in the MR report

*Authors:* *A. Massuet-Vilamajó[1], S. Medrano-Martorell[1], S. Castañer-Llanes[2], Y. Rodríguez-Álvarez[1], S. González-Ortiz[1], C. Pineda-Ibarra[1], F.D. Choque-Chávez[1], A. Jareño-Badenas[1]; [1]Barcelona/ES, [2]Badalona/ES*

**Learning objectives:** To be able to identify the relevant findings described in patients with Ménière's disease (MD), learn to grade cochlear and vestibular hydrops, assess the status of the hemato labyrinth membrane and know how to identify the obliteration of the vestibular aqueduct.

**Background:** The 2015 criteria (AAO-HNS 1995 and Barany Society) recognise two probabilistic categories of disease: definite DM and probable DM. However, diagnosis remains difficult in patients who do not have the typical triad of symptoms. Furthermore, there is no specific test for DM.
One of the major advances in the diagnosis of DM has been the visualisation of endolymphatic hydrops by magnetic resonance imaging (MRI) with delayed acquisition after intravenous administration of gadolinium-based contrast media (GCBM).
The combined presence of endolymphatic hydrops and increased blood-perilymphatic barrier permeability is associated with the clinical diagnosis of definite MD and not with other inner ear pathologies associated with vertigo. These findings may help to differentiate patients with vertigo due to MD.

**Imaging findings and procedure details:** We have selected the most representative images from the cases seen at our centre to illustrate our findings.
We show images with cochlear and vestibular hydrops of different severity and how to identify the different degrees of dilatation of the endolymphatic space at the level of the cochlea and vestibule according to the Bernaerts classification scale. We show how to identify the alteration of the blood-perilymphatic membrane by identifying increased uptake in the basal spiral of the cochlea of the affected ear in the 3D FLAIR sequence. We show how to identify the presence of the vestibular aqueduct, a linear duct from the posterior edge of the temporal bone to the vestibule, and how to assess its degree of obliteration using Attyéa's three-stage grading system.

**Conclusion:** The aim of this work is to familiarise head and neck radiologists with magnetic resonance imaging of Ménière's disease, how to grade hydrops and identify changes in the blood-perilymphatic membrane, and how to assess the status of the vestibular aqueduct, which will allow the diagnosis of Ménière's disease in patients with possible Ménière's disease and guide management towards a more conservative approach if bilaterality is identified, and avoid endolymphatic sac surgery in patients without visible VA.

### More than just a toothache: Magnetic resonance imaging of dental pathology

*Authors: H. Atkinson, P. Broadhurst, V. Vishwanath, A. Sengupta, D. St Leger, M. Dykes; Manchester/GB*

**Learning objectives:**

1. Describe the normal dental anatomy as visualised on MRI and suggest protocol optimisation to best display the relevant structures.
2. Examine selected clinical scenarios in which MRI can complement CT in the assessment of dental disease.
3. Review the MRI appearances of inflammatory and neoplastic dental disease.

**Background:** A range of dental pathologies commonly present for imaging evaluation and are generally assessed using multi-detector or cone-beam CT due to its ability to resolve minute bony detail. In contrast there has been more restricted use of MRI and comparatively little discussion in the radiological literature regarding its role in this area.

MRI provides additional useful information to CT, specifically relating to superior delineation of the soft tissue structures of the mouth, excellent sensitivity for early bone marrow changes and supplementary information such as diffusion restriction. In view of this, MRI may be under recognised in the assessment of odontogenic infection and as a problem solving tool for ambiguous mandibular lesions. Moreover dental disease is common in the general population worldwide and it is important for radiologists working in this area to recognise any dental pathology incidentally identified on MRI of the head and neck performed for other reasons in order to enable early clinical evaluation.

**Imaging findings and procedure details:** In this educational review we present several cases from our single tertiary centre that highlight the utility of MRI in imaging dental pathology and provide guidance on its use in key clinical scenarios. We examine using MRI for the assessment of the origin and complications of odontogenic infection, differentiation of cystic mandibular lesions that may appear identical on CT and the relevance of dental findings in post-treatment oral cancer patients, with a particular focus on osteoradionecrosis and medication-related osteonecrosis. In addition we discuss the possible pitfalls in image acquisition specific to the dental region and suggest areas for protocol optimisation. All cases utilise standard sequences available on commercially available MRI scanners and were acquired at 1.5T.

**Conclusion:** MRI is an underutilised element of the imaging assessment of patients with dental pathology and provides supplementary information compared to CT in the evaluation of odontogenic infection, characterisation of primary dental tumours and in the post-treatment imaging of patients with oral cancer. Awareness of this area is crucial for all radiologists working with dental and maxillofacial clinicians or reporting MRI of the head and neck more generally.

### More Than Meets The Eye: A pictorial review of key orbital imaging features to aid difficult diagnosis

*Authors: C. Ritchie, R. Dwivedi; Edinburgh/GB*

**Learning objectives:** This case series will focus on imaging for orbital pathologies based on four common clinical presentations. Imaging clues will be highlighted to show how radiology plays a key role in difficult orbital pathology diagnosis.

**Background:** Imaging can play a crucial role in patient pathways for acute orbital presentations, given potential sight threatening consequences. A variety of imaging modalities can be utilised, with CT imaging being the mainstay of acute presentations and MR imaging for better soft tissue assessment. Understanding key imaging features and lesion characteristics in orbital pathologies can help the radiologist to provide more focused differentials and play a vital role in diagnosis and management.

**Imaging findings and procedure details:** Case-based pictorial reviews will be used to illustrate key pathological features for four common ophthalmology presentations - pain, proptosis, ophthalmoplegia and orbital mass. Important differentials and key imaging clues will be explored for each presentation.

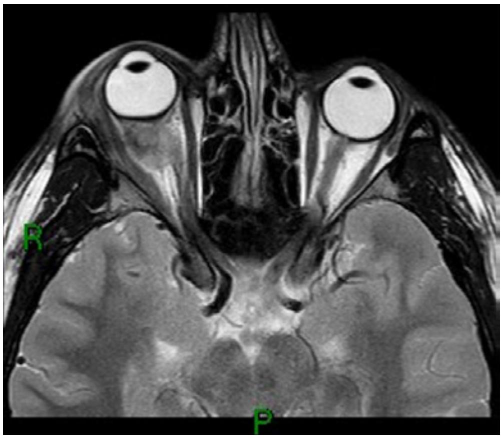

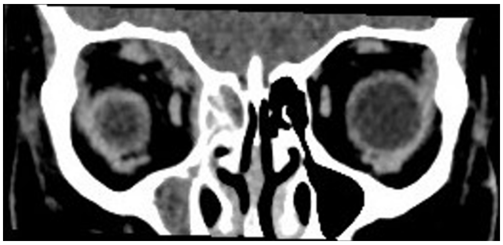

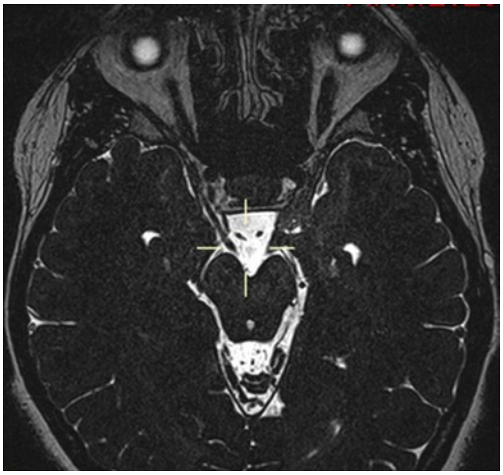

**Conclusion:** Orbital pathology can present with a variety of imaging features, and potential sight-threatening consequences, requiring a good understanding of clinical and radiological knowledge to tease differentials. Using case-based series to illustrate key imaging features and clues, this project provides a guide to diagnosis based on four most common presentations encountered in the clinical setting, ultimately to improve patient management.

### MRI of Parotid Gland Tumours Impacting Management Decisions: From diagnosis to post treatment assessment

*Author:* *N. Chakrabarty; Mumbai/IN*

**Learning objectives:**

1. To be well versed with MRI anatomy and variations of parotid gland.
2. To be able to differentiate benign from malignant and characterize majority of the parotid tumours using MRI signatures.
3. To be able to stage malignant parotid tumours on MRI which will impact treatment decisions.
4. To be able to differentiate post-treatment change from recurrence on MRI and use neck imaging reporting and data system (NI-RADS) template to help decide further management.

**Background:** 70% of salivary gland tumours are located in parotid gland; which is divided into superficial and deep lobe based on the facial nerve line, Utrecht line, and retromandibular vein on MRI. MRI provides excellent soft tissue resolution and is the modality of choice for characterizing parotid gland tumours and identifying tumours in deep lobe. Pleomorphic adenoma is the commonest parotid gland tumor followed by Warthin tumor (seen exclusively in parotid gland). Mucoepidermoid carcinoma (MEC) is the commonest malignant parotid tumor.

**Imaging findings and procedure details:** T1WI, T2WI, DWI, ADC, and post contrast T1WI are the basic MRI sequences for parotid gland, and 3D double-echo steady-state with water excitation sequence may be additionally performed for facial nerve evaluation.

MRI provides accurate location of parotid gland tumour which is important for surgical planning; superficial versus total parotidectomy with/without facial nerve resection. Conservative non-surgical approach may be suggested for willing patients with Warthin tumour as they do not undergo malignant transformation, and for this, precise diagnosis is essential. Fine needle aspiration is inadequate and non-diagnostic in 8% of cases, and if MRI findings are highly suggestive of Warthin (figure 1), then further invasive core needle biopsy can be obviated.

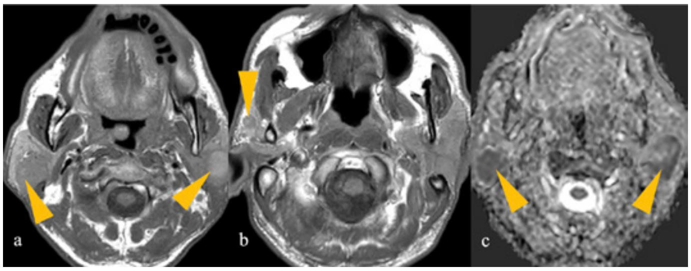

Axial T1WI (a and b) reveal well defined lesions (arrowhead) in superficial lobes of bilateral parotid glands showing hyperintense components and diffusion restriction on ADC (arrowheads in c) suggestive of Warthin tumours.

MRI plays a pertinent role in providing AJCC TNM staging for malignant parotid tumors, which has bearing on management decisions. Adenoid cystic carcinoma has a high propensity for perineural spread; which is best evaluated on MRI. Imaging features highly suggestive of malignancy are extraglandular extension, infiltrative margins, size > 4 cm, perineural spread, metastatic nodes with/without extranodal extension (ENE). MRI can also help in characterizing malignant tumours (figure 2).

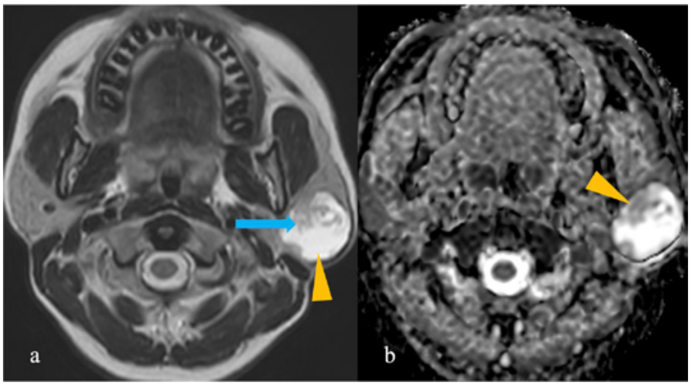

Axial T2WI (a) shows a 3 cm sized well-defined cystic lesion (arrowhead) in superficial lobe of left parotid gland, with solid intermediate signal intensity papillary projections within the cyst (arrow) showing diffusion restriction (arrowhead in b) on ADC (b), suggestive of stage T2 mammary analogue secretory carcinoma. N0 (as no neck nodes).

MRI helps differentiate post-treatment change from recurrence (figure 3) and based on the American College of Radiology (ACR) NI-RADS category, further management plan can be decided upon.

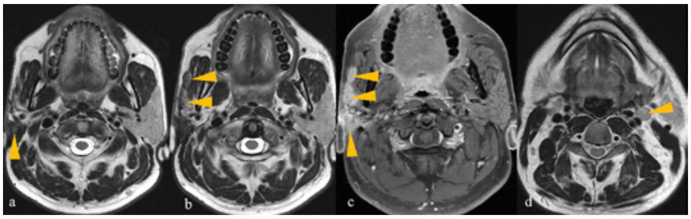

MRI of recurrent mucoepidermoid carcinoma post total right parotidectomy. Axial T2WI (a & b) reveal intermediate signal intensity lesions at operative bed, showing homogeneous post contrast enhancement (c), highly suspicious for recurrence, NI-RADS 3. Axial T2WI (d) shows a round left level II node with loss of fatty hilum, NI-RADS 2 for neck node.

MRI reporting template for parotid gland tumour is shown in figure 4.

**Magnetic resonance imaging (MRI) reporting template for parotid gland tumour**

Clinical history:

Physical examination findings:

Indication of MRI: Treatment naïve (Baseline imaging)/Posttreatment scan

MRI sequences:

**Findings:**

***Treatment naïve (Baseline imaging)***

Laterality: Right/Left/Bilateral Multicentricity: Present/Absent

Location within parotid gland: Superficial lobe/Deep lobe/ Entire parotid gland

Size of tumour: ≤ 2 cm/ > 2 to ≤ 4 cm/ > 4 cm

T1 hyperintensity: Present/Absent

Signal intensity on T2WI (> 50% of the solid component): Hyperintense/Low to intermediate

Cyst: Present/Absent

Cyst with papillary projections: Present/Absent

Diffusion restriction: Present/Absent

Extraglandular infiltration: Present/Absent

Structures infiltrated (if extraglandular extension present):

Facial nerve: Involved/ Not involved/ Cannot be commented

Perineural spread: Present/Absent

Location of perineural spread (if present):

Intracranial extension of tumour/Perineural spread: Present/Absent

Bone involved: Yes/ No/Indeterminate* Name of bone/bones involved:

Other findings:

Metastatic neck nodes: Present/Absent/Indeterminate$

Level of nodes if involved:

Size of nodes (if involved):

Extranodal extension (ENE): Present/Absent

***Posttreatment scan#***

Primary site:

Neck node:

Perineural spread:

Impression: Benign/Indeterminate/Malignant

Radiological primary tumour (T) and regional node (N) stage (if malignant) on baseline scan:

Diagnosis or differential diagnosis:

* Computed tomography (CT) required for further evaluation

$ USG guided fine needle aspiration (FNA) required

\# Neck Imaging Reporting and Data System (NI-RADS) category to be mentioned

Magnetic resonance imaging (MRI) reporting template for parotid gland tumour

**Conclusion:** MRI plays a pertinent role in parotid tumour management by localizing and characterizing parotid gland tumours, staging malignant tumours, and differentiating post-treatment change from recurrence.

References:
[1] Kim, S. Y., Borner, U., Lee, J.-H., Wagner, F., & Tshering Vogel, D. W, (2022), Magnetic resonance imaging of parotid gland tumors: a pictorial essay, BMC Medical Imaging, 22(1), 191.
[2] Fujii H, Fujita A, Kanazawa H, Sung E, Sakai O, Sugimoto H, (2019), Localization of Parotid Gland Tumors in Relation to the Intraparotid Facial Nerve on 3D Double-Echo Steady-State with Water Excitation Sequence. , AJNR Am J Neuroradiol, 40(6):1037-1042.
[3] Geiger, J. L., Ismaila, N., Beadle, B., Caudell, J. J., Chau, N., Deschler, D., Glastonbury, C., Kaufman, M., Lamarre, E., Lau, H. Y., Licitra, L., Moore, M. G., Rodriguez, C., Roshal, A., Seethala, R., Swiecicki, P., & Ha, P, (2021), Management of Salivary Gland Malignancy: ASCO Guideline. , Journal of Clinical Oncology, 39(17), 1909–1941
[4] Vlantis AC, Ng SK, Mak CK, Cheung JM, Chan AB, van Hasselt CA, (2016), If cytology of Warthin tumor is accurate, can management be conservative? , Ear Nose Throat J. , Apr-May;95(4-5):185-8.
[5] Kashiwagi N, Nakatsuka SI, Murakami T, Enoki E, Yamamoto K, Nakanishi K, Chikugo T, Kurisu Y, Kimura M, Hyodo T, Tsukabe A, Kakigi T, Tomita Y, Ishii K, Narumi Y, Yagyu Y, Tomiyama N. , (2018), MR imaging features of mammary analogue secretory carcinoma and acinic cell carcinoma of the salivary gland: a preliminary report. , Dentomaxillofac Radiol. , Jul;47(5):20170218

### Multiparametric magnetic resonance of salivary gland tumors – Can we be mistaken?

*Author: K. Markiet; Gdansk/PL*

**Learning objectives:** To understand the reasons beyond radiological misdiagnoses in salivary gland tumors.
**Background:** Multiparametric magnetic resonance imaging (mpMRI) of salivary gland tumors is a well established method allowing discrimination of focal lesions with high sensitivity, specificity and diagnostic accuracy. However, in everyday clinical practice we encounter cases in which histopathological results come in contradiction with initial radiological diagnosis.
**Imaging findings and procedure details:** This education poster is a case based review with focus on step-by-step image analysis in patients with contradictory radiological and histopathological diagnoses. Different aspects of possible incongruities are discussed.
**Conclusion:** mpMRI is a valuable tool in diagnostic algorithm of salivary gland tumors imaging. Understanding of its advantages and limitations is crucial when approaching a diagnosis.

### Neoplastic transformation of ectopic glandular tissues in the head and neck region: A pictorial review

*Authors: N. Ranjakunalan, C. Wang, S. Foo QW, C.L. Ho; Singapore/SG*

**Learning objectives:** Identify characteristic imaging features and location of ectopic glands with neoplastic transformation in the head and neck region.
**Background:** Unlike the native salivary and thyroid glands, ectopic glandular tissues found in the head and neck region have a greater tendency for malignant transformation. Early tumour detection on imaging is essential for timely and effective management.
**Imaging findings and procedure details:** Patients with head and neck neoplasms were identified during a retrospective review of imaging performed at our tertiary hospital in Singapore from 2018 to 2023. Examples of neoplasms arising from ectopic salivary include carcinoma ex-pleomorphic adenoma, mucoepidermoid carcinoma, adenoid cystic carcinoma and lymphoma. Ectopic thyroid tissues found anywhere between the tongue and diaphragm, can undergo malignant transformation. These neoplasms are highlighted in the following cases.

Case 1

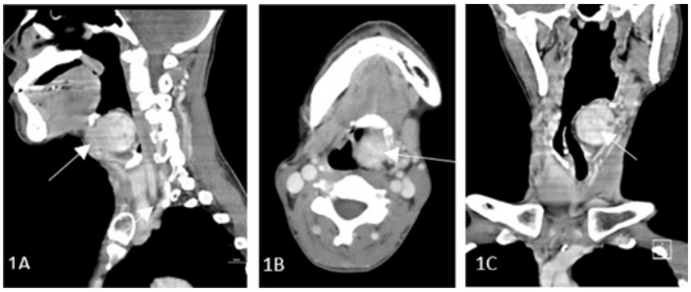

Figure 1. A 39-year-old female presents with anterior neck swelling.

Contrast-enhanced (CE) CT neck in axial (1A), sagittal (1B) and coronal (1C) planes reveal a 4cm enhancing submucosal lesion (arrows) in the pre-epiglottic space and left paraglottic region of the larynx with compression of the supraglottic airway and the left pyriform sinus. The patient underwent surgical excision and histopathology revealed a papillary thyroid carcinoma. Total thyroidectomy and neck dissection was subsequently performed.

Case 2

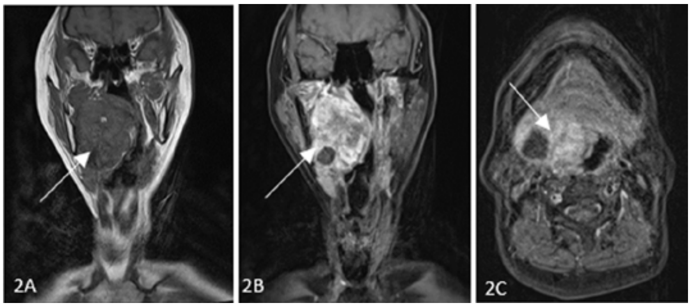

Figure 2. A 66-year-old male presents with a palpable mass within the oral cavity.

MRI neck in coronal T1w (2A), coronal post-contrast T1w fat suppressed (2B) and axial post-contrast T1w fat suppressed (2C) planes reveal an 8 cm well-defined, avidly enhancing right parapharyngeal mass with narrowing of the oropharynx (arrows). The patient underwent surgical resection and histopathology revealed carcinoma ex pleomorphic adenoma.

Case 3

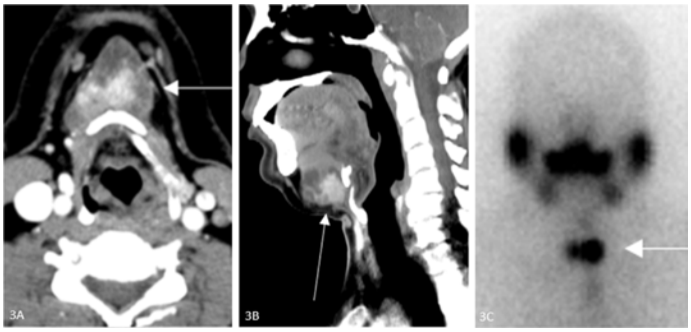

Figure 3. A 44-year-old male presents with an anterior neck lump.

Axial (3A) and sagittal (3B) contrast-enhanced CT neck shows a 2.5cm lesion (arrows) anterior to the hyoid and thyroid cartilages. Initial diagnosis was thyroglossal duct cyst and he underwent a Sistrunk procedure. Histopathology revealed papillary thyroid carcinoma with nodal metastasis. After total thyroidectomy, he underwent Iodine-131 scintigraphy (3C) which revealed remnant thyroid lesion (arrow). Radioactive iodine ablation therapy was administered subsequently.

**Conclusion:** Ectopic salivary and thyroid tissues tend to undergo malignant transformation. Multimodality imaging plays a vital role in timely patient management including tumour detection, pre-operative planning and post-operative surveillance.

### Non epithelial tumor of larynx causing “Pseudo-palsy” of vocal cord

*Author:* *S. Solav; Pune/IN*

**Purpose/Objectives:** Non epithelial lesion of vocal cord is identified as vocal cord palsy/ impaired movement without obvious mucosal ulceration. There are several nonepithelial lesions of vocal cord. Lymphoma, plasmacytoma, liposarcoma, lipoma, myxofibrolipoma, hemangioma, rhandomyosarcoma are amongst the important differential diagnosis. These tumors occur in the submucosal layer of larynx and hence the mucosa appears intact at endoscopy. The lesions are typical non circumscribed and invasion of adjoining structures helps in differentiating benign from malignant lesions.Presented here is proposed new terminology of “pseudo-palsy” of vocal cord secondary to its involvement by NHL.

**Methods and materials:** A 39 years old woman presented with change of void and dysphonia. Videolaryngoscopy revealed impaired movement of left vocal cord suggesting its palsy. There was no superficial ulceration.
F18 FDG PET CT scan was performed using 8 millicurie of F18- FDG (on Siemens Horizon PET CT system) on overnight fasting state. Images were interpreted by one expert and verified by another expert independently.

**Result:**

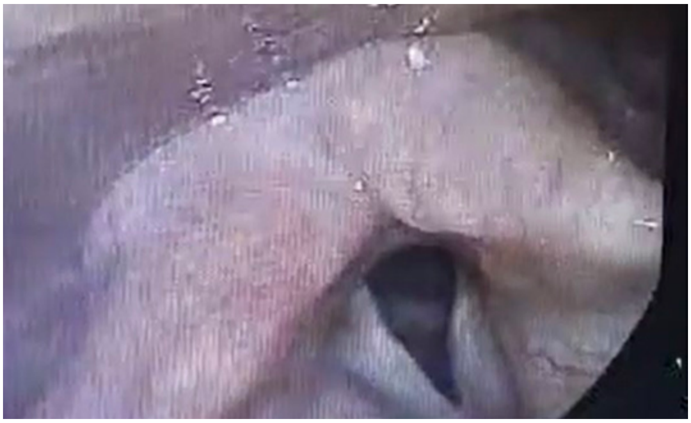

Laryngoscopy

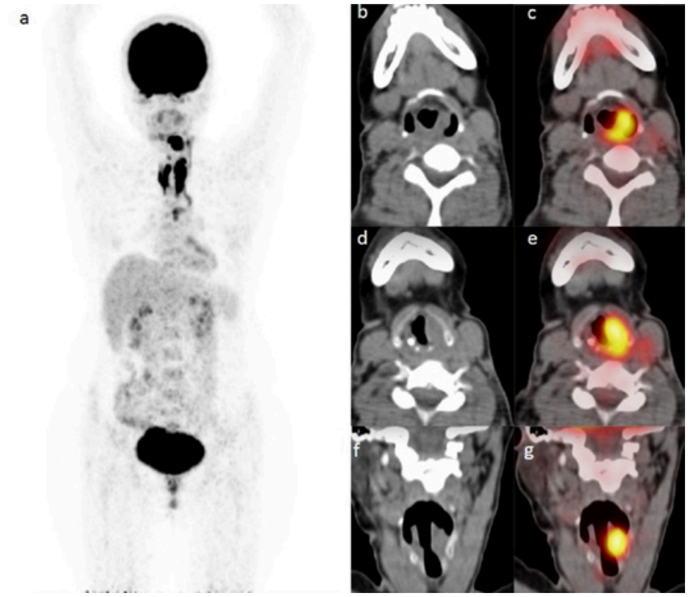

FDG PET CT

Videolaryngoscopy showed left vocal cord palsy with no mucosal ulceration or lesion. (Fig 1)
CT showed an ill-defined enhancing lesion in left vocal cord 15 x 11 x 18 mm. PET CT scan performed showed hypermetabolic transglottic left vocal cord lesion 17 x 11 mm SUV 17.6. (Fig 2 b,c,d,e,f,g) The lesion showed contiguous extension in left aryepiglottic fold with effacement of left laryngeal ventricle. Incidentally Thyroid gland showed diffuse inflammation. There was no node in AP window or in the path of vagus nerve from skull base. Histology confirmed the diagnosis of NHL.

**Conclusion:** Non epithelial lesions of vocal cord present with impaired vocal cord motion. The superficial mucosa is usually intact. Hence, on endoscopy it gives an impression of vocal cord palsy as in our present case. FDG PET revealed hypermetabolic left transglottic lesion. There was an FDG avid left level III neck node. However, there was no lesion in the pathway of vagus nerve to suggest neurogenic involvement. Hence, we would like to coin the term "Pseudo-palsy" of vocal cord due to non-epithelial lesion (NHL in this casee) which implies impaired vocal cord movement secondary to tumor bulk giving a false impression of palsy on endoscopy.

References:
Katilmis H, Ozturkcan S, Ozdemir I, et al. A clinico-pathological study of laryngeal and hypopharyngeal carcinoma: correlation of cord-arytenoid mobility with histopathologic involvement. Otolaryngol Head Neck Surg. 2007;136(2):291–295. doi: 10.1016/j.otohns.2006.08.022.
Saleh EM, Mancuso AA, Stringer SP. CT of submucosal and occult laryngeal masses. J Comput Assist Tomogr 1992;16(1):87–93.

### Not so innocent: Practical approach to complications of sinonasal infections

*Authors: A. Bozer, Y. Pekçevik, I.B. Arslan; Izmir/TR*

**Learning objectives:**
- Understanding the anatomic relationship of paranasal sinuses to orbital and intracranial compartments.
- Recognizing imaging characteristics of orbital and intracranial complications of acute bacterial rhinosinusitis.
- Understanding the importance of early diagnosis in preventing life-threatening complications.

**Background:** Clinical findings help predicting complication of rhinosinusistis include eyelid edema, erythema, chemosis, proptosis, blurred vision, fever, headache, altered mental status and double vision. Early identification of complications of the acute bacterial rhinosinusitis particularly orbital and intracranial, is critical due to associated risks and high morbidity and mortality rates. Computed Tomography (CT) or Magnetic Resonance Imaging (MRI) aid in diagnosis, with MRI preferred for its sensitivity in detecting orbital and intracranial complications. We aim to explain the anatomical relationship of paranasal sinuses and give a practical approach for early detection of the complications.

**Imaging findings and procedure details:**

Anatomic Relationship of Paranasal Sinuses: The anatomical relationship of paranasal sinuses to orbital and intracranial compartments is crucial to understand for understanding route for infection spread and preticting early complications.

Orbital Complications: Complications classified by Chandler et al. include:
–Preseptal cellulitis
–Orbital cellulitis
–Subperiosteal abscess
–Orbital abscess
–Cavernous sinus thrombosis

Intracranial Complications:
–Subdural and epidural empyema
–Focal cerebritis and brain abscess
–Meningitis
–Sinus thrombosis

Osseous and extracranial:
–Pyocele
–Osteomyelitis
–Facial cellulitis
–Pott's Puffy tumor

**Conclusion:** Prompt recognition and management of complications are crucial in acute bacterial rhinosinusitis to prevent life-threatening outcomes. Understanding anatomical relationships and employing appropriate imaging modalities facilitate accurate diagnosis and intervention.

**Keywords:** Acute bacterial rhinosinusitis, Complications, Magnetic Resonance Imaging, Computed Tomography.

### Orbital fractures – A practical approach to facial trauma

*Authors:* H. Yamauchi, A. Baba, N. Ogino, H. Ojiri; Tokyo/JP

**Learning objectives:**
Orbital fractures are a common craniofacial trauma encountered in daily clinical practice, and are aesthetically significant because they result from a variety of causes, including traffic trauma, sports injuries, and accidents involving falls. CT has a key role in the accurate diagnosis of orbital fractures and in determining the course of treatment. The aim of this poster is to highlight:

1. Radiological anatomy of orbits and its clinical relevance
2. What radiologists need to know while reporting typical orbital fractures
3. Pictorial review of naso-orbital-ethmoid and zygomaticomaxillary fractures associated with orbital fractures

**Background:** The complexity of the facial skeleton makes the characterization of orbital fractures according to the exact anatomical nomenclature of the specific bone affected very difficult. Facial bones have structural fragile or firm areas, and an understanding of the facial structural system is important when considering the diagnosis and treatment of fractures. Therefore, imaging may be more clinically useful not only to evaluate the fracture site and identify the fracture type, but also to describe the fracture according to its relationship and proximity to functionally related and vulnerable soft tissues.
**Imaging findings and procedure details:** The orbits consist of seven bony elements (maxillary, palatine, zygomatic, lacrimal, ethmoidal, sphenoidal, and frontal bone). Orbital fractures are broadly classified into orbital blow-out fractures and orbital rim fractures. In orbital blow-out fractures, important imaging findings to evaluate for linear fracture (closed type), ocular depression, enlargement of the orbital volume, and entrapment of extraocular muscles are important in determining the treatment strategy. Fractures of the orbital rim show fractures that dislocate the supraorbital or inferior wall into the orbit and may be accompanied by impingement of the external ocular muscles. Fractures of the supraorbital wall are often accompanied by other facial fractures such as those of the frontal sinus, and intracranial complications such as pneumoencephalus and cerebral contusion should be noted.
**Conclusion:** Imaging plays an important role in orbital fractures, identifying not only the type of fracture but also the fracture associated with the orbital fracture and ultimately determining treatment.

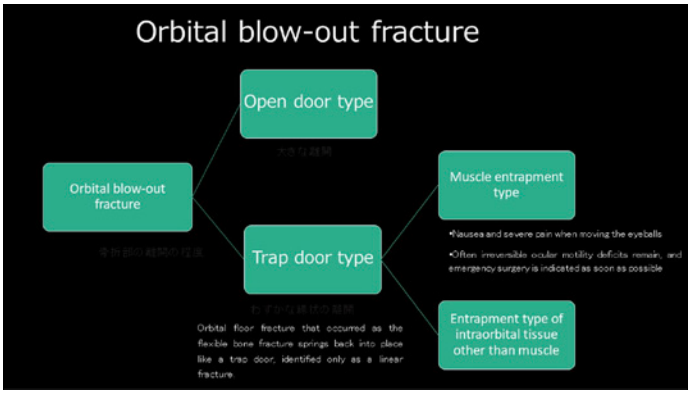

Orbital blowout fracture

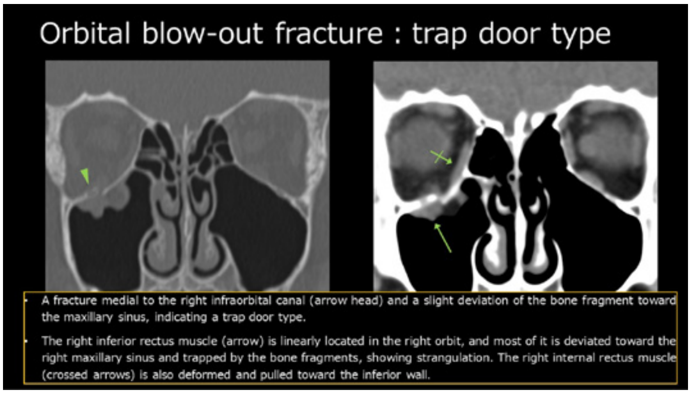

Orbital blow-out fracture : trap door type

### Orbital tumefactive lesions: Our experience

*Author:* M. Douka, A. Galani, V. Koutoulidis; Athens/GR

**Learning objectives:** To display some of the most frequently encountered in our daily practise, orbital lesions, and analyse the imaging charecterestics that orientate the general radiologist to the correct diagnosis.
**Background:** Over a period of a year, we collected the most characterestic cases of orbital pathology from our department. The MRI studies were performed on a 1.5 or a 3 T scanner, while the CT studies were conducted in a 128-slice scanner. The MRI protocol included T1mDIXON and T2mDIXON axial and coronal sequences, axial STIR and DWI as well as T1mDIXON post gadolinium administration.
**Imaging findings and procedure details:** We recorded two cases of orbital lymphoma, the one presenting with a massive low signal intensity, space occupying soft tissue lesion, surrounding the globe and causing marked exophthalmos. It showed diffusion restriction, almost homogenous enhancement, extending to the cavernous sinus, the pterygopalatine and the infratemporal fossa. Following two months of chemotherapy, the mass dramatically reduced in size, with a remnance of ill-defined soft tissue lesion of the intraconal fat, affecting mainly lateral rectus and oblique muscles, while the perineural spread was also present in a lesser extend. In the follow-up exams we recorded minor enhancement and gradual fibrosis, resulting in enophthalmos.
In a woman with a history of breast cancer, presenting with painful reduced orbital motility, we recorded an infiltrative mass, causing enlargement of the lateral and inferior rectus muscles, extending intraconally. In the differential diagnosis, metastasis (histologically proven) and orbital pseudotumor were suggested.
Two cases of perineural spread, from nasopharyngeal and oropharyngeal carcinoma, were presented as space occupying, moderately enhancing lesions of the orbital fissures, infiltrating the inferior and medial rectus muscles, with extension through the V2 and V3 branches to the Meckel's cave[1][2].
In a woman with Waldenstrom macroglobulinemia and gradual visual acuity decline, we found a massive enlargement, abnormal signal and tortuosity of both optic nerves, up to the chiasm, as well as abnormal signal of the left optic tract, compatible with Bing-Neel syndrome. Findings subsided after treatment initiation.
A case of optic nerve meningioma was diagnosed in a young woman, with an indermediate signal intensity lesion surrounding the nerve, enlarging the optic foramen and extending to the cavernous sinus, with homogenous enhancement.
In a man with known NSC lung cancer, with visual disturbancies, a crescent-shaped choroidal lesion was found, compatible with choroidal metastasis and focal retinal detachment[3].

Axial T1W images post contrast administration, at presentation (on the left) and two months after chemotherapy (on the right), show the lesion of the left orbit, histologically proved as lymphoma.

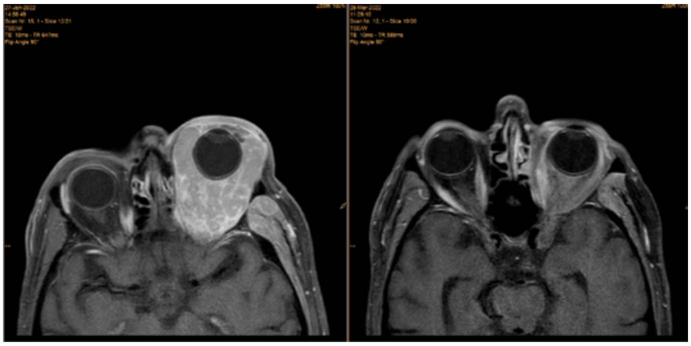

Ill-defined lesion of the right lateral and inferior rectus muscles, with intraconal extension and fat obliteration, histologically proved as metastatic from breast cancer.

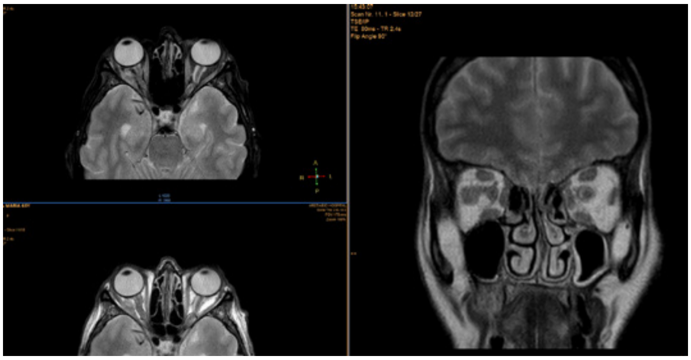

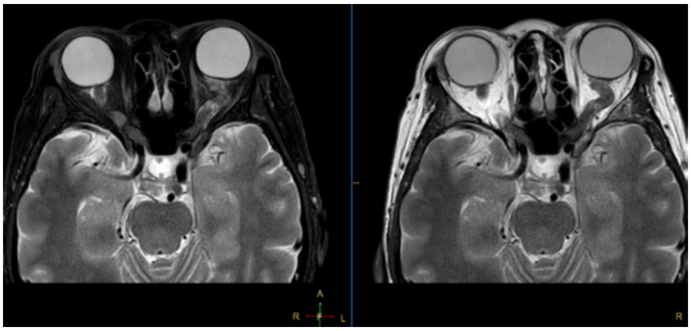

T2W images of the optic pathways, show marked enlargement, abnormal signal and tortuosity of the left and to a lesser extend, the right optic nerve (Bing-Neel syndrome)

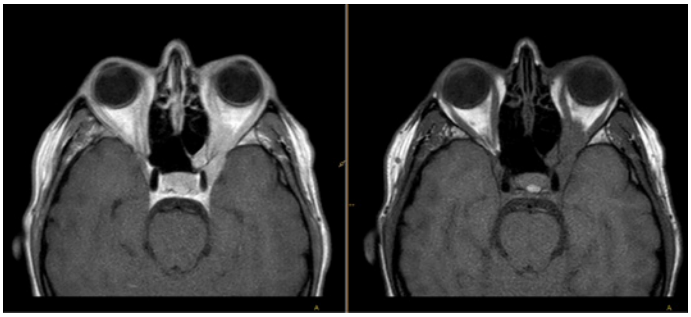

T1W images pre (right) and post (left) contrast enhancement, of a left optic nerve meningioma, extending to the cavernous sinus, 6 months post Cyberknife.

**Conclusion:** Some of the most characterestic cases of orbital space occupying lesions are presented above, with imaging correlation.

References:
[1] Michael O'Neill, (2012), CT and MRI Imaging Spectrum of Orbital Masses: A Pictorial Essay and Review of the Literature, Cureus, e279, 4(10)
[2] Naveen Mysore et al., (2012), Adult Orbital Masses: A Pictorial Review, Canadian Association of Radiologists Journal, 63, 39-46
[3] Lin SH, Xu YG, Zhao JH, Cui H, Jin H, Jia YJ, Zhao J, Li YJ., (2021), Choroidal metastasis with retinal detachment, Medicine (Baltimore), e28009, 100(51)

### Ortner's Syndrome: When the Heart Stops Speaking

*Authors:* *L. Ellis, S. Sadiq; Swansea/GB*

**Learning Objectives:** The objectives of this educational poster are to present a case of Ortner's syndrome in a patient who attended the ENT department with a hoarse voice. After defining Ortner's syndrome and presenting the case, the poster will outline the anatomy of the recurrent laryngeal nerves and discuss different causes of this clinical entity, concentrating on the cardiovascular system. Review areas of the cardiovascular anatomy on a CT chest will also be suggested for use when presented with a patient who has a vocal cord palsy on laryngoscopy or CT, in the absence of a laryngeal cause.
**Background:** Ortner's syndrome is uncommon and characterised by a left vocal cord palsy secondary to an underlying cardiovascular pathology[1]. The left and right recurrent laryngeal nerves innervate the majority of the intrinsic laryngeal muscles allowing movement of the true vocal cords. On the left side the nerve branches from the vagus nerve at the aortic arch level, loops through the aortopulmonary window posterolateral to the ligamentum arteriosum, then ascends in the tracheoesophageal groove[2]. Its close relationship to multiple mediastinal cardiovascular structures puts it at risk of injury or compression when there is pathology here. However, patients with Ortner's syndrome are likely to initially present to ENT clinicians due to their symptom of a hoarse voice. It is therefore an important differential for Head and Neck radiologists to be aware of so that, in the context of a vocal cord palsy in an otherwise normal larynx, the cardiovascular structures in the chest are scrutinised for underlying pathology [3]. Identifying this syndrome enables the patient to be referred to and treated by the appropriate clinical team in a timely manner.
**Imaging Findings and Procedure Details:** This poster describes a patient who presented with a unilateral left vocal cord palsy. CT neck did not identify a cause. CT thorax however identified a dilated pulmonary artery with minimal space in the aortopulmonary window. Compression of the left recurrent laryngeal nerve here was felt to be the cause of the patient's vocal cord palsy.
**Conclusion:** In conclusion, Ortner's syndrome is an uncommon but important differential for both Head and Neck and also General Radiologists to be aware of, as it is a cause of unilateral vocal cord palsy which requires referral to a specialty outside of ENT. These patients however are often initially referred to ENT clinicians due to their presentation of a hoarse voice. Scrutinising the anatomical course of the left recurrent laryngeal nerve, and in particular the cardiovascular mediastinal structures for pathology, will aid timely identification and appropriate treatment for these patients.

References:
[1] Verma S, Talwar A, Talwar A, Khan S, Krishnasastry KV, Talwar A., (2023), Ortner's syndrome: A systematic review of presentation, diagnosis and management., Intractable Rare Dis Res., 141-147, 12(3), doi: 10.5582/irdr.2023.01047, 2024-03-02
[2] Di Muzio B, Ma J, Bell D, et al., (2023), Recurrent laryngeal nerve., Reference article, Radiopaedia.org, https://doi.org/10.53347/rID-41456, 2024-03-02
[3] Paquette CM, Manos DC, Psooy BJ., (2012), Unilateral vocal cord paralysis: a review of CT findings, mediastinal causes, and the course of the recurrent laryngeal nerves., Radiographics. , 721-40, 32(3), doi:10.1148/rg.323115129, 2024-03-02

### Paediatric head and neck lumps: A diagnostic approach and pictorial essay of the common and some unfamiliar pathologies

*Authors:* M. Deshmukh[1], N. Bhatt[1], S. Patel[2]; [1]Luton/GB, [2]Bedford/GB

**Learning Objectives:**
1) Imaging modality of choice of paediatric head and neck lumps.
2) Brief overview of embryology of common developmental neck lumps.
3) A diagnostic approach to paediatric head and neck lumps, categorised into solid vs cystic, and anatomical distribution within the neck.
4) Pictorial essay of some common paediatric head and neck pathologies and not so common pathologies that radiologists should be aware of.

**Background:** Paediatric head and neck lumps are a common presentation in healthcare, encompassing a range of entities including benign and malignant[1]. As such paediatric neck lumps can be a source of anxiety for parents as well for healthcare professionals, including the reporting radiologist[2]. We will demonstrate a simple approach all radiologists can use when faced with a paediatric neck lump, provide a brief overview of basic embryology relevant to a couple of developmental related neck lumps, highlight examples of common paediatric head and neck lumps with a description of typical imaging appearances and finally show a series of imaging of less common pathologies that reporting radiologists should be aware of[3].

**Imaging Findings or Procedure Details:**
Cases include
- Thyroglossal duct cyst
- Branchial cleft cyst
- Dermoid
- Necrotic mass
- Ewing sarcoma
- Giant cell tumour
- Orbital lymphangioma
- Neuroblastoma
- Eosinophilic granuloma

**Conclusion:** With an understanding of basic embryology and anatomy, all reporting radiologists should be able to apply a logical approach to diagnosing paediatric head and neck lumps. Sometimes the radiologist may encounter less common pathologies; we provide examples of such cases with brief descriptions to help radiologists familiarise themselves with these entities.

References:
[1] Meuwly JY, Lepori D, Theumann N, Schnyder P, Etechami G, Hohlfeld J, Gudinchet F, (2005), Multimodality imaging evaluation of the pediatric neck: techniques and spectrum of findings, Radiographics , 931-48
[2] Brown RE, Harave S, (2016), Diagnostic imaging of benign and malignant neck masses in children-a pictorial review, Quantitative Imaging in Medicine and Surgery, 591-604
[3] Mittal MK, Malik A, Sureka B, Thukral BB, (2012), Cystic masses of neck: A pictorial review, Indian Journal of Radiology and Imaging

### Parotid lesions: A retrospective summary of a single institution experience

*Authors:* M. Douka, I. Diamantopoulos, A. Galani, L.A. Moulopoulos; Athens/GR

**Learning Objectives:** To analyse the imaging findings of some common pathologies found in the parotid gland, from our cases in the last year.

**Background:** The majority of the patients presented with a palpable lesion at the anatomical site of the parotid gland, and were referred for an MRI examination. The exams were performed on a 1.5 T scanner, with the imaging protocol including axial and coronal T1 and T2 weighted sequencies, and contrast enhanced axial and coronal T1W sequences[1].

**Imaging Findings and Procedure Details:** The first patient presented with a swelling of the right parotid for more than a year, which on imaging corresponded to a multiloculated, well-demarkated lesion, of the superficial parotid lobe, with markedely increased signal on T2W images, inhomogeneously enhancing. A smaller lesion was also detected on the left parotid, of similar imaging features. The larger lesion was found to be a pleomorphic adenoma on following biopsy (Im.1).
The second patient, with a known history of Sjogren's disease, presented with a left parotid asymmetrical enlargement. On MRI examination we found a bilateral parotid gland swelling, with multiple hyperintense on T2W images small lesions, compatible with the microcystic appearance, typical of Sjogren's syndrome. Moreover, on the left superficial parotid lobe, a well-defined lesion of inhomogenous texture, mixed solid, with areas of cystic degenaration, was noticed, with diffusion restriction and contrast enhancement of the solid compartement. Another lesion of similar imaging characterestics was found on the superficial lobe of the right parotid (Im.2). The lesions were attributed to Warthin tumors (histology expected)[2].
Another examinee was referred with a right parotid gland lesion, but on MRI we found five lesions of similar characterestics, in both parotids (superficial and deep lobe), the largest of which on the right superficial lobe, of a maximum diameter of 4 cm. The lesions, lobulated, mostly cystic, with hemorragic components and enhancing septa, were attributed to Warthin tumors, given a history of formerly surgically excised such lesion (Im.3).
In a patient with Gorlin-Goltz syndrome[3][4], we found a space ocuppying lesion between the maseter muscle and the left parotid, with ill-defined margins and of inhomogeneously increased T2 signal (with septa and foci of low T2 signal). The lesion showed avid contrast enhancement, with small hypovascular regions , and regions of restricted diffusion. Moreover, two ovoid lesions of identical T2 signal were found on the upper cervical region, attributed to infiltrated lymph nodes (level IIb). The findings were suspicious for basal cell carcinoma (Im.4).

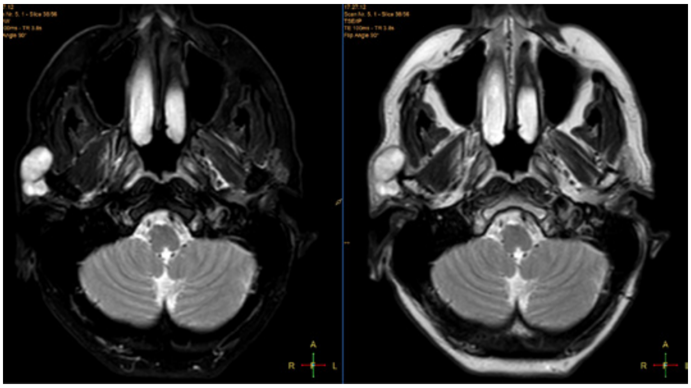

T2W images of a multiloculated markedly hyperintense lesion of the superficial lobe of the right parotid.

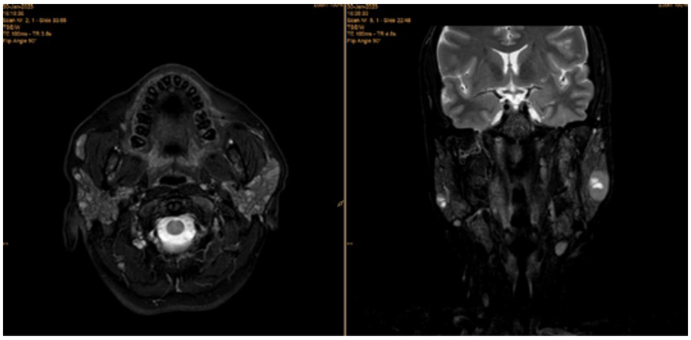

Axial (left) and coronal (right) T2W images of a patient with Sjogren's disease, with bilateral superficial parotid lobe lesions.

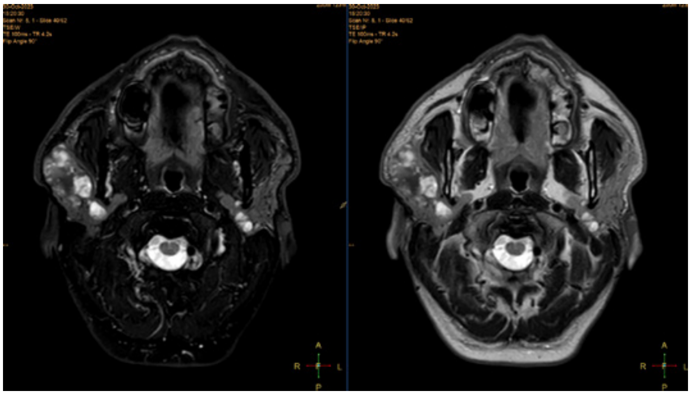

Axial T2W images of bilateral mixed cystic and solid lesions of the superficial and deep parotid lobes (Warthin tumors).

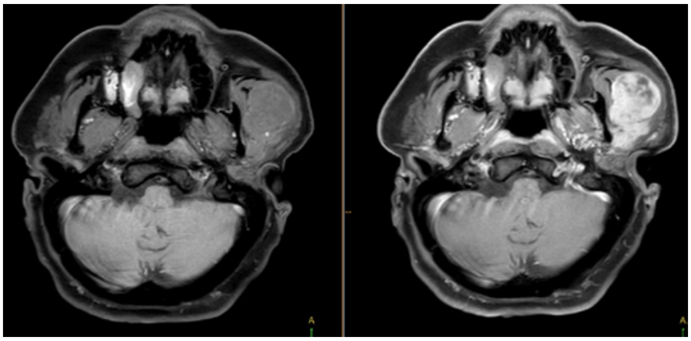

Axial T1W images pre (left) and post (right) contrast administration, showing a space occupying lesion with infiltrative margins, between left parotid and maseter muscle, with avid enhancement, in a patient with Gorlin-Goltz syndrome.

**Conclusion:** Parotid lesions from our experience, with histological correlation.

References:
[1] Suzuki S, Bandoh N, Goto T, Kubota A, Uemura A, Kono M, Sato R, Takeda R, Sakaue S, Yamaguchi Isochi T, Yamaguchi Isochi T, et al, (2022), A retrospective study of parotid gland tumors at a single institution, Oncology Letters, 207, 24, no. 1 (2022)
[2] https://www.eurorad.org/case/1856
[3] https://radiopaedia.org/articles/gorlin-goltz-syndrome-1
[4] Hegde S, Shetty SR, (2012), Radiological features of familial Gorlin-Goltz syndrome, Imaging Sci Dent, 55-60, 42(1)

### Perineural tumour spread (PNS) in head and neck malignancies: A case-based review highlighting radiologic signs and clinical significance

*Authors: M.S. Swarup, G. Sindhwani, M. Jain, R. Gupta Ghasi; New Delhi/IN*

**Learning Objectives:**
1. To discuss briefly relevant anatomy and patterns of perineural tumor spread (PNS) from various primary sites of malignancies
2. To discuss imaging signs (direct and indirect) to detect PNS in various head and neck malignancies
3. To provide key points for evaluation of each nerve for PNS
4. To provide quick search checklist to detect PNS on imaging

**Background:** Perineural spread (PNS) or growth along nerves is a distinctive feature of head and neck maliganancies. Identification of PNS has important therapeutic and prognostic implications and is critical for adequate staging and treatment planning. Presence of PNS/PNI is associted with higher risk of local recurrence, higher risk of metastasis, and significant decreased 5-year survival rate. Radiologic identification and delineation of PNS may impact a change in extent of surgical resection (limited vs extensive), and change in selection of radiation treatment field. Adenoid cystic carcinoma of salivary glands and mucosal squamous cell carcinomas constitute majority of cases. PNS occurs most frequently along maxillary, mandibular and facial nerves. Imaging diagnosis is important to map full tumour extent and to avoid tumour progression from unrecognized PNS. Imaging findings in PNS include thickening and/or enhancement of nerve branches; widening, destruction of skull base neural foramen or canal; small tumoral lesions at some distance from primary site, in a neural 'crossroad' such as pterygopalatine fossa (PPF) or Meckel's cave; and denervation atrophy of muscles supplied by affected nerve.

**Imaging Findings and Procedure Details:** We will provide a case-based discussion of PNS in various head and neck malignancies with a multi-modality imaging approach including contrast-enhanced CT and MRI. These include;
a. Adenoid cystic carcinoma and mucoepidermoid carcinoma parotid – facial nerve
b. Adenoid cystic carcinoma submandibular gland – inferior alveolar nerve
c. Squamous cell carcinoma (SCC) buccal mucosa – inferior alveolar nerve
d. SCC oral cavity – branches of trigeminal nerve
e. SCC lower lip/chin – mental nerve
f. Carcinoma hard palate – greater palatine nerve
g. Nasopharyngeal carcinoma – branches of trigeminal nerve
h. Rhabdomyosarcoma masticator space – mandibular division of trigeminal nerve (TN)
i. Inflammatory myofibroblastic tumour – mandibular division of TN
j. Post operative recurrent SCC in flap margin with PNS along mandibular nerve
k. Post operative SCC hard palate and maxillary alveolus with recurrence involving PPF – PNS along maxillary and vidian nerves

**Conclusion:** Radiologists should be familiar with the pattern of growth and spread in various head and neck malignancies, especially the perineural tumour spread patterns, their radiologic appearance and clinical signifificance for optimal patient management.

### Pictorial review of schwannomas in the head and neck: With histology and pathophysiology

*Authors:* *Y. Kubo, T. Mori, S. Yoshimoto, M. Kusumoto; Tokyo/JP*

**Learning Objectives:**
1. To review imaging findings of schwannomas in the head and neck.
2. To recognize radiological features with histological correlation of schwannoma.
3. To correlate the cranial nerve origins of schwannomas with associated clinical symptoms or sequelae.

**Background:** Schwannomas are benign, common tumors that may arise along the course of the peripheral nerves or cranial nerves, anywhere in the head and neck.
Because schwannomas arise from the sheath of a peripheral nerve, resection of the tumor sometimes causes nerve damage or compromises the affected nerve.
Occasionally, it is clinically difficult to distinguish schwannomas from other soft-tissue tumors.
Therefore, radiologists need to recognize the imaging findings of schwannomas as well as its histology and pathophysiology.
**Imaging Findings and Procedure Details:** Schwannomas are painless masses with well-defined borders, good mobility, and a tendency toward slow enlargement. The characteristic histologic patterns of schwannomas include the Antoni type A and Antoni type B regions.
The target sign is one of the characteristic imaging findings in schwannomas that distinguishes it from other soft tissue tumors.

Typical imaging findings of schwannomas: Target sign

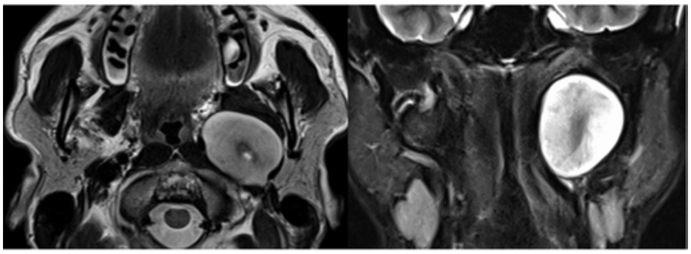

- The target sign is shown on T2-weighted images as signal intensity showing a biphasic pattern in the central and marginal areas
- The central Antoni A region has a hypointense area reflecting the densely arranged cellular and fibrous components, while the Antoni B region with edematous stroma has a hyperintense area.

On T2-weighted images, schwannomas show a heterogeneous mixture of hypointense and hyperintense. This heterogeneity is attributed to the mixture of areas of densely arranged cells (Antoni type A) and areas of loosely arranged cells (Antoni type B), resulting in variable cellularity and water content.

T2WI with histological correlation of schwannoma

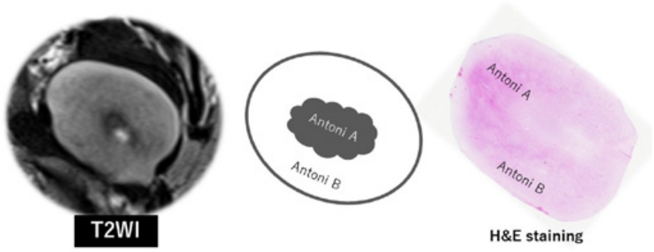

- Typically, Antoni A areas are centrally located and Antoni B areas are peripherally located.
- The location of Antoni A and Antoni B may be biased due to the cut surface of the pathology specimen.

Schwannomas commonly cause neurological symptoms associated with the cranial nerve. Imaging evidence of end-organ compromise can be useful in identifying the cranial nerve as a prognostic indicator of the risk of treatment-related nerve palsy.
**Conclusion:** Recognizing Antoni A and B histotypes that may affect the imaging characteristics of schwannomas is useful in their radiological diagnosis.
Knowledge of cranial nerve anatomy may assist in the diagnosis of schwannomas.

### Picturing Peril: A Radiologist's Odyssey Through Head and Neck Infections

*Authors:* *A. Ecenarro Montiel, A. Arango, V. Trujillo, L.A. Mateos, M.C. Vieira; Santiago de Compostela/ES*

**Learning Objectives:**
- Understanding the importance and causes of deep infections of the head and neck.
- Explaining the urgent management of these entities and the role of the radiology department: indications, protocols, and reporting.
- Reviewing the relevant anatomy for the spread of infections and the main complications.

**Background:** Head and neck infections present diagnostic challenges due to their varied etiology and overlapping clinical features. Radiological imaging plays a crucial role in accurately diagnosing and characterizing these infections, guiding appropriate management strategies.
**Imaging Findings and Procedure Details:** This educational poster comprehensively outlines the imaging characteristics of the most prevalent types of head and neck infections.
The radiological assessment is crucial to decide whether a patient will need surgical drainage or not, which is why performing the appropriate study and knowing how to interpret it will be so important.
Furthermore, it provides a systematic approach to conducting radiological studies of head and neck infections, emphasizing the importance of standardized protocols and comprehensive reporting to ensure accurate diagnosis and effective patient management.
**Conclusion:** The urgent radiological management of acute cervical infections is fundamental in planning their treatment.
It is crucial to perform an imaging study that can assess the infection, its extent, and potential complications.
Understanding the anatomy of deep cervical spaces helps us to make a correct diagnosis, reflect it appropriately in the report, and predict the extent of infections.

### Pleomorphic adenoma of salivary glands – Really pleomorphic on imaging and presentation!!

*Authors:* *M.S. Swarup, K. Gehlot, G. Sindhwani, M. Jain, R. Gupta Ghasi; New Delhi/IN*

**Learning Objectives:**
1. To familiarise with the variable and pleomorphic presentation and imaging appearance of pleomorphic adenomas (PA) of salivary glands
2. To discuss the common and uncommon CT and MR imaging features of PA of the salivary glands

**Background:** Pleomorphic adenoma (PA) is the most common salivary gland tumor, with most common site of origin being the parotid glands. It is characterized by histological and architectural diversity. PA is seen in a wide age group with variety of presentation, most typical being a slowly growing periauricular mass. It can show a great variety of imaging appearance, some of which are specific to diagnosis. Intralesional density and signal intensity varies according to the cellular density, proportion of epithelial and stromal components, and type of stromal components. Besides this, a number of secondary histological changes, such as fibrosis, lipometaplasia, ossifcation, cystic degeneration, hemorrhage, necrosis, and infarction do occur in PAs, albeit rarely. These pose difficulty in preoperative diagnosis and differentiation from other salivary gland tumors.

**Imaging Findings and Procedure Details:** PAs appear as well-defined lesions, often with lobulated contours on cross sectional imaging, with progressive and persistent enhancement in delayed phase on dynamic post contrast perfusion studies. Typical PA shows marked hyperintense signal on T2 weighted images (may be more than CSF signal in spinal canal) due to its abundant myxochondroid stroma, and a hypointense fibrous capsule. PA usually shows high ADC values on Diffusion weighted images, exception being the cellular variant of PA.

Atypical presentations in the form of mass in the cheek or palate can be possible in cases of origin from accessory parotid tissue and minor salivary gland of palate respectively. Malignant transformation can occur in long standing PAs.

We will present a case based review of PA of various salivary glands with common/typical and uncommon/ atypical presentation and imaging morphology.

a. Typical/ classic PA
b. Cellular variant of PA
c. Large PA with infarction, hemorrhage and necrosis
d. PA with predominant fibrosis
e. PA with lipomatous component
f. PA with ossification/ calcification
g. PA with cystic degeneration
h. Recurrent PA - multicentricity
i. Carcinoma Ex-Pleomorphic adenoma
j. Carcinosarcoma Ex-Pleomorphic adenoma
k. PA of accessory parotid tissue
l. PA of palate (minor salivary gland)
m. PA of parotid in infancy

**Conclusion:** Radiologists should be aware of these presentations of PAs and familiar with the diverse histological and radiological findings for correct diagnosis and differentiation.

### Post-thyroidectomy follow-up of well-differentiated thyroid cancer patients: The role of neck ultrasound

*Authors:* *E. Mpiliouri, M. Kosmidou, M. Koutsounida, E. Xinou, E. Sinduka, E. Karoglou; Thessaloniki/GR*

**Learning Objectives:**
- Neck ultrasound (US) techniques and normal US findings in post-thyroidectomy patients with well-differentiated thyroid cancer (DTC)
- US findings of local recurrence in DTC patients
- Recommended workup for suspicious or indeterminate lymph nodes (LNs)

**Background:** Although DTC is related to a high 10-year survival rate, local recurrence is common especially to regional LNs. Post-thyroidectomy follow-up (FU) includes serum tumor markers (thyroglobulin-Tg) and thyroid antibodies combined with neck US. Neck US is considered gold standard regardless of the risk of recurrence or the therapeutic approaches used.

**Imaging Findings and Procedure Details:**
- In post-thyroidectomy US the thyroid bed and neck compartments from the submental region to the jugular notch should be examined.
- In the central compartment the thyroid bed should normally consist of a narrow fibrofatty, triangular or longitudinal hyperechoic tissue.
- Remnant thyroid tissue is an important finding, because it is an indication of radioactive iodine (RAI) therapy (Fig1).

- The risk of recurrence in the central compartment is very high and it should be examined thoroughly.
- Small hypoechoic lesions, taller-than-wide with irregular margins and microcalcifications are typical of local recurrence (Fig2) and should be discriminated from post-operative fibrosis and parathyroid adenoma.
- Postsurgical lymphocele should be differentiated from cystic LN (Fig3).

- The majority of LN local recurrences occurs in neck compartments III, IV and VI, whereas skip metastases in levels IIa and Vb.
- An important rule in evaluating suspicious LNs in the setting of DTC is that size does not matter.
- Round LNs with irregular margins and abnormal blood supply pattern are typical of metastatic LNs.
- Hyperechogenicity, presence of calcifications (macro or micro) and cystic degeneration are also US findings suggestive of LN metastasis (Fig4).
- Small (<8mm), non-palpable, hypoechoic LN without obvious blood supply are considered indeterminate and watch and wait is recommended. In case of size increase biopsy/FNA is needed.
- FNA and Tg washout is recommended for every suspicious LN≥8-10mm. FNA should be avoided if management is unaffected by a negative result.
- Correlation of US findings with serum Tg levels is essential, because when serum Tg is within normal limits, the possibility of LN recurrence is very low (<2%).
- If postsurgical complication is suspected, US may be needed in order to differentiate haematoma from hygroma or oedema and to guide aspiration avoiding important structures (Fig3).

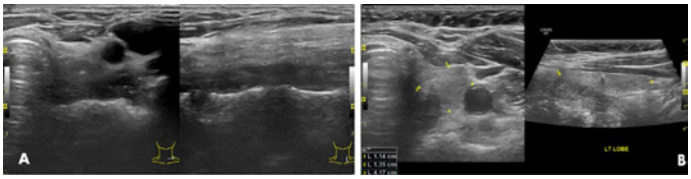

Figure 1. Thyroid bed: a) Normal thyroid bed in transverse (left) and sagittal (right) plane with hyperechoic fibrofatty tissue. b) Sizable hypoechoic and well-marginated tissue suggestive of remnant thyroid gland in transverse (left) and sagittal (right) plane.

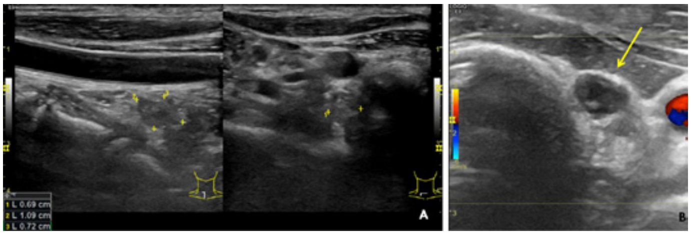

Figure 2. Local recurrence of DTC in the thyroid bed: a) Paratracheal, hypoechoic, ill-marginated area, obliterating the tracheal margin. b) Hypoechoic, well-marginated, oval structure with microcalcifications.

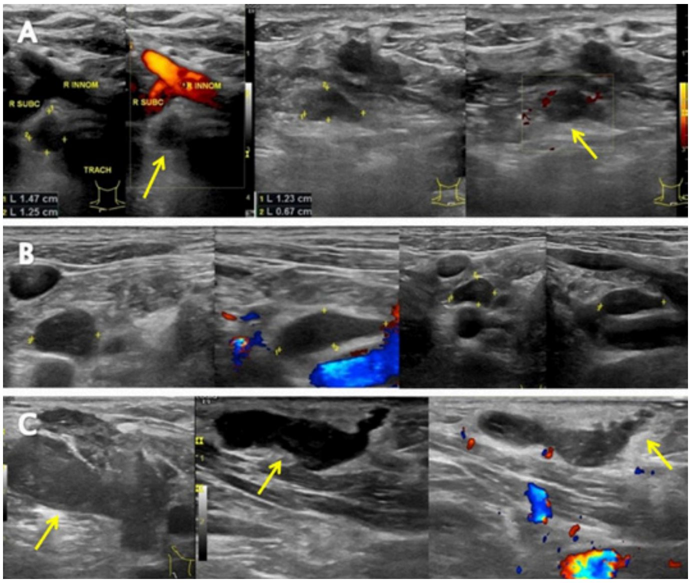

Figure 3. Differential diagnosis of local recurrence: A) Parathyroid adenoma: hypoechoic with peripheral blood supply in level VII, could be mistaken for a LN. B) Postsurgical lymphocele: hypoechoic lesion with irregular margins, could be mistaken for a cystic LN. C) Postsurgical hematoma and hygroma (middle), after a difficult thyroidectomy.

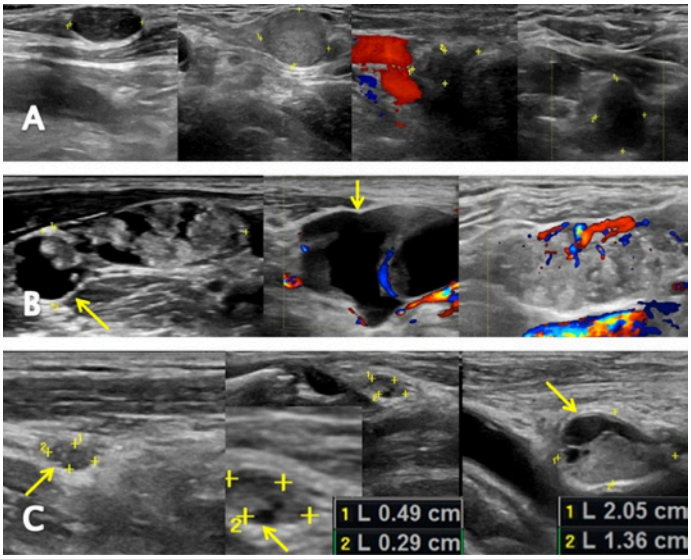

Figure 4. Sonographic features indicative of metastatic LNs: A) Oval or round shaped, hypoechoic or hyperechoic, irregular margins, taller-than-wide. B) Cystic degeneration of varying degrees, chaotic and centripetal blood supply pattern. C) Size doesn't matter; small lymph nodes with cystic degeneration and microcalcifications.

**Conclusion:** Being aware of the neck US technique and the post-thyroidectomy normal and suspicious US findings is essential for diagnosing local recurrence of DTC and for guiding management.

### Radio-induced sarcomas of the head-neck district: Imaging features of a case series in our experience at National Center for Oncological Hadrontherapy (CNAO) in Pavia, Italy

*Authors: M. Pecorilla, S. Imparato, C. Fichera, M.F. Achilli, A. Chalaszczyk, M. Rotondi, M.R. Fiore, E. Orlandi, L. Preda; Pavia/IT*

**Learning Objectives:** To discuss the main imaging features in Magnetic Resonance Imaging (MRI) of radio-induced sarcomas of the head and neck region by retrospectively analyzing the cases that came to our attention at the National Center for Oncological Hadrontherapy (CNAO) in Pavia.

**Background:**

| CASE | YEAR OF RT TREATMENT | AGE AT DIAGNOSIS OF RIS | YEAR OF ONSET RIS | LATENCY PERIOD |
|---|---|---|---|---|
| **Osteosarcoma** | 1983 | 45 | 2020 | 37 |
| **Osteo-chondroblastic osteosarcoma** | 2015 | 42 | 2016 | 1 |
| **Undifferentiated sarcoma** | 2012 | 74 | 2022 | 10 |
| **Spindle cell sarcoma** | 2012 | 59 | 2021 | 9 |
| **Chondroblastic osteosarcoma** | 1998 | 30 | 2013 | 15 |
| **Chondroblastic osteosarcoma** | 2007 | 69 | 2015 | 8 |
| **Spindle cell sarcoma** | 2012 | 27 | 2016 | 4 |
| | | mean age: 49.42 years | | mean latency period: 12 years |

Time of onset of radio-induced sarcoma

We analyzed seven cases of radio-induced sarcomas of the head-neck district evaluated in our Center from 2014 to 2023. Seven patients were selected, three women and four men, with a mean age at diagnosis of 49.42 years (range 27-74 years). The mean latency period for sarcoma development was 12 years (range 1-37 years). The histological types of sarcomas were osteosarcoma (57%), undifferentiated sarcoma (14%) and spindle cell sarcoma (29%). The mean dose of radiotherapy treatments performed on the primary tumours was 63.49 Gy. Clinical data on primary diagnosis, radiotherapy treatment and latency period of radio-induced sarcoma development were collected. We reviewed contrast-enhanced MRI images of examinations performed at baseline and non contrast CT acquired before treatment.

**Imaging Findings and Procedure Details:** Patients underwent MRI with 3T system (Skyrafit - Siemens) by employing turbo spin-echo, gradient-eco and diffusion techniques with a head and neck combined coil. We used a standard protocol for the head-neck study with T1-weighted, T2-weighted, DWI and T1-weighted fat-sat isotropic post-contrast sequences.
The slice thicknesses were 3 or 4 mm for the axial and coronal plane, with distant factor 0-10%.
The analysis of the MRI images of the radio-induced sarcoma cases showed a homogeneity of signal characteristics, in particular with hypo-hyperintensity in the T2-weighted sequences and inhomogeneous contrast enhancement predominantly peripheral. These features are overlapping with those described in the literature. [1][2]
The evaluation of oncological and radiotherapy history showed a coincidence of the anatomical region between the site of onset of the radio-induced sarcoma and the site of the radio-treated primary tumour.

| CASES | T1w | T2w | FS | DWI restriction | CE | Non-contrast CT |
|---|---|---|---|---|---|---|
| **Osteosarcoma** | isointense | iso/hyperintense | / | yes | inhomogenous, predominantly peripheral | iso/hypodense |
| **Osteo-chondroblastic osteosarcoma** | isointense | hypo/hyperintense | no | yes | inhomogenous, predominantly peripheral | isodense, with huge calcifications |
| **Undifferentiated sarcoma** | iso/hypointense | hypo/slightly hyperintense | no | yes | inhomogenous, predominantly peripheral | isodense, with huge calcifications |
| **Spindle cell sarcoma** | isointense | hypo/isointense | / | yes | inhomogenous, predominantly peripheral | / |
| **Chondroblastic osteosarcoma** | iso/hypointense | hypo/slightly hyperintense | no | yes | inhomogenous, predominantly peripheral | isodense, with huge calcifications |
| **Chondroblastic osteosarcoma** | isointense | mainly hyperintense | no | yes | inhomogenous, predominantly peripheral | isodense, with huge calcifications |
| **Spindle cell sarcoma** | isointense | slightly hyperintense | no | yes | inhomogenous, predominantly peripheral | isodense |

MR and CT features of radio-induced sarcoma

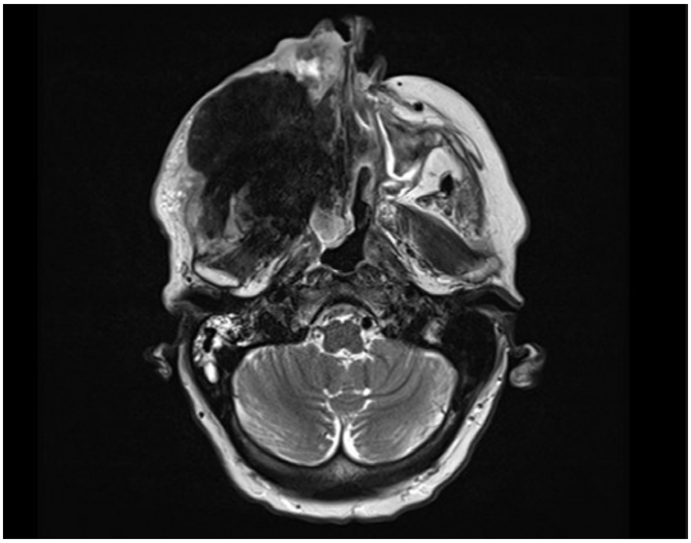

T2w section of radio-induced undifferential sarcoma of right maxillary region (case 3)

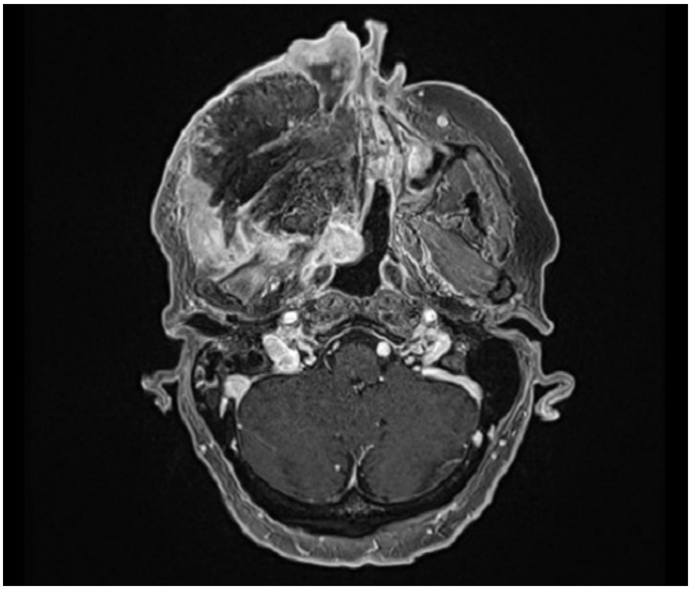

T2w section of radio-induced undifferential sarcoma of right maxillary region (case 3)

**Conclusion:** Radiation-induced sarcomas are rare disease with low prevalence and small number of cases despite center with large case series of patients undergoing radiotherapy. The head and neck region is a rare site for the development of radio-induced sarcomas. Imaging plays an essential role in the identification and characterisation of the neoplasm. The radiologist has to be aware of the radiological aspect and of the different sites where radio-induced sarcoma might occur in order to optimize diagnosis and treatment.[3][4]

References:
[1] Cai PQ, Wu YP, Li L, Zhang R, Xie CM, Wu PH, Xu JH., (2013), CT and MRI of radiation-induced sarcomas of the head and neck following radiotherapy for nasopharyngeal carcinoma., Clinical Radiology, https://pubmed.ncbi.nlm.nih.gov/23477476/
[2] Makimoto Y, Yamamoto S, Takano H, Motoori K, Ueda T, Kazama T, Kaneoya K, Shimofusa R, Uno T, Ito H, Hanazawa T, Okamoto Y, Hayasaki K., (2007), Imaging findings of radiation-induced sarcoma of the head and neck. , British Journal of Radiology, https://pubmed.ncbi.nlm.nih.gov/17908819/
[3] Lou J, Jiang L, Dai X, Wang H, Yang J, Guo L, Fang M, Wang S., (2021), Radiation-Induced Sarcoma of the Head and Neck Following Radiotherapy for Nasopharyngeal Carcinoma: A Single Institutional Experience and Literature Review, Frontiers , https://pubmed.ncbi.nlm.nih.gov/33552942/
[4] Laurino S, Omer LC, Albano F, Marino G, Bianculli A, Solazzo AP, Sgambato A, Falco G, Russi S, Bochicchio AM., (2022), Radiation-induced sarcomas: A single referral cancer center experience and literature review. , Frontiers , https://pubmed.ncbi.nlm.nih.gov/36249019/

### Radiological assessment of post-treatment Head and Neck cancers and the concept of NIRADS: What radiologists should know

*Authors: M.S. Swarup, S. Sree V S, G. Sindhwani, M. Jain; New Delhi/IN*

**Learning Objectives:**
1. To provide an image-based pictorial demonstration of various categories of ACR - NIRADS lexicon in post-treatment cases of head and neck malignancies
2. To discuss imaging surveillance algorithm and category-wise recommendations in treated head and neck cancers
3. To discuss descriptors of the NIRADS lexicon like non-mass-like soft tissue, masses and mucosal abnormality on imaging (CECT/ PET-CT, CEMRI)
4. To provide a NIRADS reporting template for efficient communication of findings with radiation oncologists and surgical oncologists

**Background:** Interpretation of cross-sectional imaging of treated head and neck malignancies is challenging due to the altered and distorted anatomy secondary to complex surgery, in addition to changes from chemo-radiotherapy. Neck imaging reporting and data system (NIRADS) was developed for surveillance imaging in patients with treated head and neck cancers for better care. This provides numerical levels of suspicion of disease recurrence and a standardized approach with linked management recommendations. Radiologists play a critical role in the early detection of particularly deeper tissue recurrences, at a stage when patients remain favourable candidates for intervention.
**Imaging Findings and Procedure Details:** We will provide an image-based review of different categories of the NIRADS lexicon in various post-treated cases of HNC with multi-modality imaging approaches including MRI and CECT/PET-CT. Post-treatment imaging has been typically performed after at least 12 weeks in all cases. In the post-treatment imaging study, the status of disease at the primary site and regional nodes was simplified into five categories of NIRADS Category 1: No evidence of recurrence Category 2: Low suspicion of disease recurrence Category 3: High suspicion Category 4: Definite recurrence
**Conclusion:** The post-treatment neck is often a source of anxiety for diagnostic radiologists, leading to suboptimal reporting and often no standardized guidance is provided for the next management steps. Familiarizing with the use of the NIRADS lexicon will allow the generation of concise and efficient reports to effectively communicate results with linked recommendations.

### Retro-molar trigone: Navigating through the No Man's Land

*Authors: J. Asogan Vaishnavi[1], S. Thiru[1], A. Kamalasanan[2]; [1]Dundee/GB, [2]Glasgow/GB*

**Learning Objectives:**
1. Radiological anatomy of retromolar trigone and its clinical relevance
2. Clinical presentation of retromolar malignancy
3. Role of Imaging in retromolar malignancy
4. Pictorial review of TN staging of retromolar malignancy with a systematic approach

**Background:** Retromolar trigone (RMT), an important oral cavity sub site is a triangular mucosal region located posterior to the last lower molar tooth and pointed backward, towards the maxillary tuberosity. RMT malignancy grows indolently and presents usually with advanced disease. Clinical evaluation often underestimates the actual deep tumour spread and thus imaging plays a vital role in estimating exact extent which is critical for surgical and radiotherapy treatment planning. The main intent of this exhibit is to familiarise the readers with the RMT malignancy with special emphasis on important anatomic relationship with its surrounding structures. Our educational exhibit illustrates with examples various stages of RMT malignancy and various patterns of tumour spread
**Imaging Findings and Procedure Details:** The initial modality of choice for evaluation of RMT malignancy is contrast enhanced CT with soft tissue and bone reformats. Also, "puffed cheek" technique may help to detect small tumours. Further evaluation with contrast enhanced MRI may help to assess marrow involvement and peri-neural spread. Also, MRI is less affected by dental amalgam and helps with accurate T-staging of tumours due to superior soft tissue resolution.
*While reporting, mentioning the following aspects can help in planning the treatment:*
1. Size and extent of the tumour including peri-neural spread
2. Extent of mandible involvement (Extent of bone erosion, marrow involvement, buccal/lingual cortex involvement, Inferior alveolar nerve canal involvement) – *If involved, the surgical treatment is based on mandibulectomy which can be marginal or segmental and depends on the extent of bone involvement.*
3. Nodal involvement (Location, number, size, extra-capsular spread) – *modified radical neck dissection is performed with nodal involvement. In the absence of nodal involvement and if primary is being treated with surgery, selective neck dissection is performed.*
4. Distant metastasis and rule out second primary in upper aero digestive tract as chances are high

**Conclusion:** This exhibit should prompt the Radiologists to consider retro-molar trigone as an important review area, to ensure recognition of early disease and thus allow prompt diagnosis and treatment.

### Review of Skull Base Metastases

*Author: L. van Camp; Sydney/AU*

**Learning Objectives:**
1. Review the epidemiology of skull base metastases
2. Revise the anatomy of the skull base and how it relates to clinical presentation
3. Understand the imaging features of skull base metastases on CT and MRI

**Background:** Compared to other sites of skeletal metastases, skull base metastases are relatively rare with varying incidence reported in the literature ranging from 0.13-5.0% in an autopsy series [1] to up to 23% in certain cancer groups [2]. Haematogenous spread is most common and the most frequently implicated cancers are breast, prostate and lung carcinomas and lymphoma [1]. Presentation ranges from asymptomatic with metastases detected on screening CT or MRI, to symptomatic presentations with pain or cranial neuropathies which can form part of a spectrum of specific skull base clinical syndromes depending on the cranial nerve/s afftected [3]. Most often, treatment is with targeted external-beam radiation therapy, either as a stand-alone modality or in combination with surgery or chemotherapy [4].

**Imaging Findings and Procedure Details:** The skull base forms the floor of the cranial cavity and separates the brain from the extracranial structures of the head and neck. Skull base anatomy is complex namely due to the multiple canals and foramina which allow for the passage of arteries, veins and cranial nerves between the two compartments. It is involvement of these canals and foramina where the cranial nerves traverse which frequently accounts for patient's symptoms. CN VI palsy has been described as the most frequently observed neuropathy [5], however other clinical syndromes such as Gasserian ganglion syndrome (CN V), Jugular foramen syndrome (CN IX, X, XI) and occipital condyle syndrome (CN XII) have also been described (3).
Patients typically undergo both CT and MRI either as a screening examination for cranial metastases or to investigate clinical symptoms. Skull base metastases are most frequently found in the clivus, petrous apex and sphenoid bone due to the higher marrow content of these areas. On CT the metastases are often lytic, but may also have a sclerotic appearance. They are typically T1 hypointense due to replacement of the fatty marrow and can have variable T2 signal hyperintensity but usually demonstrate homogenous enhancement. Fat-suppressed T2 and T1 post gadolinium best demonstrates the lesions. Diffusion imaging has also been shown to be a useful adjunct in detecting skull base disease (2).
Five case examples will be provided to highlight the imaging features of metastases at various locations at the skull base.

**Conclusion:** Knowledge of normal skull base anatomy is essential in evaluating the imaging of asymptomatic patients with primary malignancies, as well as an understanding of cranial neuropathies in those that are symptomatic.

References:
[1] Fandino M, Yu E, & Witterick IJ. , (2013), Skull Base Metastases. , Springer, In Encyclopaedia of Otolaryngology, Head and Neck Surgery , Berlin, Heidelberg, 2474-2483, https://doi.org/10.1007/978-3-642-23499-6_94, 2024-03-22
[2] Nemeth A.J., Henson J.W., Mullins M.E., Gonzalex R.G. & Schaefer P.W. , (2007), Improved Detection of skull metastasis with diffusion-weighted MR imaging., AJNR, 1088-1092, 2024-03-22
[3] Mitsuya K, Nakasu Y, Horiguchi S, Harada H, Nishimura T, Yuen S, Asakura K and Endo M., (2011), Metastatic skull tumors: MRI features and a new conventional classification., J Neurooncol, 239-245, 104, 2024-03-22
[4] Kotecha R, Angelov L, Barnett GH, Reddy CA, Suh JH, Murphy ES, Neyman G, & Chao T., (2014), Calvarial and skull base metastases: expanding the clinical utility of Gamma Knife surgery., J Neurosurg, 91-101, 121, 2024-03-23
[5] Battal B & Zamora C. , (2023), Imaging of skull base tumors, Tomography, 1196-1235, 9, 2024-03-22

### Short case series of different causes of a cerebrospinal fluid rhinorrhoea

*Authors: A. Balodis, K. Migunova, A. Bušs, R.V. Kalğjs, U. Raits; Riga/LV*

**Purpose/Objectives:** Cerebrospinal fluid (CSF) rhinorrhea refers to a CSF leakage extracranially into the paranasal sinuses, then into the nasal cavity, and exits via the anterior nares.
CSF rhinorrhea is often seen whenever there is an osseous or dural defect of the skull base, mostly caused by head trauma or a post-operative complication of skull base surgery, or due to congenital defects.

**Methods and materials:** We present two cases of CSF rhinorrhea caused by diverse etiology and treated with different surgical approaches.

**Result:** A 72-year-old woman with a 6-month history of left-sided intermittent nasal discharge and mild headache. This was identified to be CSF caused by intrasphenoidal meningoencephalocele due to the persistence of the lateral craniopharyngeal canal (Stenberg's canal).The brain MRI identified a herniated temporal lobe through a bony defect that communicates the middle cranial fossa with the lateral recess of the sphenoidal sinus. The patient underwent a functional endoscopic sinus surgery – with the resection of meningoencephaloce and closure of the defect with fascia lata lever. There were no complications related to the surgery and no recurrence of CSF leakage in 6 months.

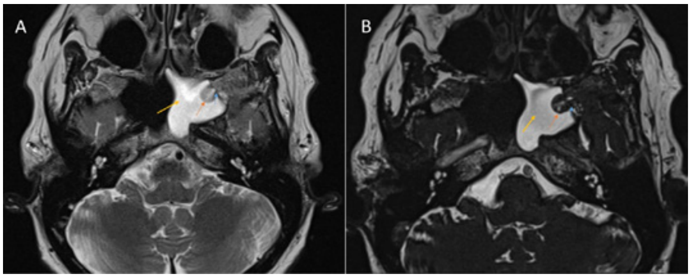

Image A: T2 weighted ax. and B:T2 3D CISS. The orange arrow marked left sphenoidal cavity with fluid, which is most likely CSF. An intrasphenoidal meningoencephalocele marked with a red arrow and the persistence of the lateral craniopharyngeal canal (Stenberg's canal) is marked with a blue arrow. [1]

A 41-year-old male with multiple skull and facial fractures (Le Fort III), a traumatic subarachnoid hemorrhage in the left frontal lobe, and traumatic brain injury resulting from altercations. He was primarily treated with subsequent facial bone osteosynthesis using mini and microplates. A month after this episode, the patient was re-admitted to another hospital due to progressive headache, subfebrile temperature, and persistent watery nasal discharge. Brain CT was repeated demonstrating diffuse pneumocephalus and cerebrospinal fluid circulation abnormalities with signs of transtentorial herniation and brain edema. Progressive widening of cerebral ventricles was noted, as well as periventricular edema, most likely, acute communicating hydrocephalus due to meningitis. The patient underwent a right ventriculostomy with an antimicrobial drain, followed by a ventriculoperitoneal shunting procedure. Shortly after, the CSF rhinorrhea resolved.

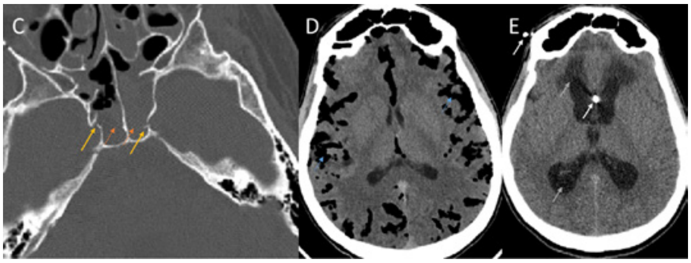

Image C. CT bone window, the yellow and red arrows shows the fractures of the walls of the sphenoid cavity with blood and CSF inside. D,E image. The blue arrow marks extensive pneumocephalus and the gray arrow at follow up images shows the developing communicating hydrocephalus which was treated with ventriculoperitoneal shunting- white arrows [2]

**Conclusion:** CSF rhinorrhoea is a relatively rare condition occurring secondary to different etiology, however, it can be clinically noticeable or insidious. A comprehensive diagnostic assessment of individuals clinically suspected of having CSF rhinorrhea is critical, along with an understanding of CSF components and imaging abnormalities.

References:
[1] Oh JW, Kim SH, Whang K., (2017), Traumatic Cerebrospinal Fluid Leak: Diagnosis and Management. , Neurotrauma., 63-67
[2] Bendersky DC, Landriel FA, Ajler PM, Hem SM, Carrizo AG. , (2011), Sternberg's canal as a cause of encephalocele within the lateral recess of the sphenoid sinus: A report of two cases., Surg Neurol Int

### Spectrum of findings and complication in patients with mesiodens

*Authors: A. Inzerillo, E. Palizzolo, M. De Angelis, F. Cordaro, O. Berlina, V. Pampalone, F. Bencivinni, A. Lo Casto.; Palermo/IT*

**Learning Objectives:** The aim of this paper is to present an analysis of findings in patients with mesiodens, detected by panoramic radiography (PR) and/or CBCT, to define different features and associated complications.
**Background:** Supernumerary teeth represent an anomaly of an excess number of the deciduous or permanent dentition, called hyperdontia. Among them, the most prevalent type is mesiodens, located in the midline between the two central incisors of the maxilla. In Christianity mesiodens represented the “sin tooth”. Also Michelangelo represented in his masterpiece “Pietà”, Christ with a mesiodens, to highlight he had collected on himself all the world sins. Usually discovered incidentally during radiological exams, mesiodens can exhibit normal orientation or appear inverted, impacted within the dental arch, with no symptoms. It may occur in normal or syndromic individuals. Mesiodens shape is distinguished in conical, the most prevalent, tuberculate, supplemental, and infundibuliform. Mesiodens may be solitary or multiple, unilateral or bilateral, with double mesiodens being rare, especially if a double inversion is also present. Triple mesiodens is also rarely reported. Mesiodens may also erupt in oral or, more rarely, nasal cavity.
**Imaging Findings and Procedure Details:** Patients with different types of mesiodens can exhibit both common and unusual imaging findings, which can be detected through PR and/or CBCT, that allows a 3D accurate evaluation of shape, number, location, relation with surrounding anatomic structures, and assessment of complications. PR may show a well-defined radiolucency behind the two central incisors of the maxilla however CBCT shows a tooth typically located palatally, behind the maxillary incisors, often solitary and conically shaped. It commonly displays an angulated orientation, either mesially or distally, and has a standard alignment or an inverted position, i.e. the crown faces the nasal cavity, while the root points toward the oral cavity. Double mesiodens occurrence is less common and may exhibit either a singular or, more rarely, a double inversion. Different complications may arise, including interference with eruption and alignment of the adjacent teeth, delayed or non-eruption of maxillary incisors, radicular resorption, dentigerous cyst formation, and nasopalatine canal encroachment. If mesiodens erupts, it may be susceptible to caries and periapical lesions. If it erupts into the nasal cavity, inflammation and rhinoliths can also occur.
**Conclusion:** Although PR is an important imaging tool for diagnosis, CBCT, thanks to 3D imaging, allows a more accurate evaluation of mesiodens, in order to make a correct therapeutic planning, reducing the risk to damage close dentomaxillary anatomy.

### Sphenoid Bone Lesions: A Practical Approach to Distinguishing Benign from Malignant Pathologies

*Authors: A. Péporté[1], A. Venkatasamy[2]; [1]Frauenfeld/CH, [2]Strasbourg/FR*

**Learning Objectives:**
1) Identify sphenoid bone lesions that can be labeled as “do-not-touch lesions” on imaging, with a high degree of certainty.
2) To propose a practical diagnostic aid for radiologists, integrating clinico-epidemiological data, computed tomography (CT), and magnetic resonance imaging (MRI) features to distinguish between inflammatory and neoplastic lesions.
3) Collaborate with skull base surgeons and pathologists to facilitate the accurate diagnosis of lesions that could be characterized on imaging alone.

**Background:** The sphenoid bone (SB) is an unpaired structure forming a crucial part of the skull base. Despite significant advancements in trans-nasal endoscopic techniques, imaging modalities remain essential for identifying and characterizing pathologies in this region. The SB can be affected by a wide spectrum of pathologic conditions, both primary and secondary[1]. CT and MRI are now the gold standard imaging modalities for assessing sphenoid abnormalities, often complementing each other due to the bone’s difficult accessibility for clinical examination. Given the intricate anatomical connections of the SB and potentially severe complications of invasive procedures, thorough evaluation is essential[2]. This underscores the pivotal role of radiologists in managing SB lesions.
**Imaging Findings and Procedure Details:** The current radiological investigation techniques employed to assess lesions in this area primarily rely on CT and MRI, serving as complementary methods for the assessment of the entire skull base. CT is particularly adept at delineating sphenoid bone profile, detecting sclerosis, lysis, erosions, and identifying intrasinusal content, as well as the extension of disease beyond the sphenoid bone. On the other hand, MRI plays a significant role in defining intracranial and extracranial soft tissue invasions, assessing potential involvement of cranial nerves, and providing further characterization of intrasinusal contents[3][4][5].
**Conclusion:** Given the challenges associated with clinically assessing the sphenoid bone (SB) and the critical importance of carefully evaluating any invasive diagnostic or therapeutic procedures involving it, radiologists bear significant responsibility in guiding towards accurate diagnoses. By possessing comprehensive expertise in imaging features, radiologists can facilitate precise non-invasive identification and characterization of lesions arising in this intricate area in the majority of cases.

References:
[1] Renata Cochinski 1, Mohit Agarwal 1, Jessica Albuquerque 1, Carolina A de Almeida 1, Rafaela P Stricker 1, Marcela F Uberti 1, Ana Paula K Casqueiro 1, Gabriel S Mendonça 1, Galba R S do Nascimento 1, Fernanda Miraldi 1, Marcos Decnop 1, (2022), Anatomy and Diseases of the Greater Wings of the Sphenoid Bone, Radiographics, 1177-1195, 42(4), https://pubmed.ncbi.nlm.nih.gov/35657765/
[2] DS Sethi, (1999), Isolated sphenoid lesions: diagnosis and management, Otolaryngol Head Neck Surg, 730-6, 120(5), https://pubmed.ncbi.nlm.nih.gov/10229601/
[3] Nardi C, Maraghelli D, Pietragalla M, Scola E, Locatello LG, Maggiore G, Gallo O, Bartolucci M. A, (2022), A practical overview of CT and MRI features of developmental, inflammatory, and neoplastic lesions of the sphenoid body and clivus, Neuroradiology, 1483-1509, 64(8), https://pubmed.ncbi.nlm.nih.gov/35657394/
[4] Srubiski A, Csillag A, Timperley D, Kalish L, Qiu MR, Harvey RJ, (2011), Radiological features of the intraosseous lipoma of the sphenoid, Otolaryngol Head Neck Surg, 617-22, 144(4), https://pubmed.ncbi.nlm.nih.gov/21493245/
[5] Bikmaz K, Mrak R, Al-Mefty O, (2007), Management of bone-invasive, hyperostotic sphenoid wing meningiomas, 905-12, 107(5), https://pubmed.ncbi.nlm.nih.gov/17977259/

### T1 Hyperintensity: A useful sign in sellar and para-sellar MRI

*Authors: P. Gupta, M.S. Swarup; New Delhi/IN*

**Learning Objectives:**
1. To provide a case-based pictorial review of various normal variants and pathologic conditions of the sellar and para-sellar region showing T1 hyperintensity on MR imaging
2. To briefly discuss the pathological basis for T1 hyperintense MR signal and classification of the lesions
3. To describe the other pertinent MR imaging findings for correct diagnosis or narrowing down differential diagnosis

**Background:** T1 signal hyperintensity is commonly observed during magnetic resonance imaging of the sellar and para-sellar region. However, the underlying sources of this signal intensity pattern vary, and its clinical significance depends on context.
Normal causes of sellar and para-sellar T1 signal hyperintensity can be attributed to vasopressin storage in the neurohypophysis, presence of bone marrow in normal and variant anatomical structures, hyperactive hormone secretion in the anterior pituitary lobe (e.g., in newborns, pregnant, or lactating women), flow related artifacts and magnetic susceptibility effects.
Pathologic conditions with T1 signal hyperintensity in sellar and para-sellar region may be attributed to clotted blood (e.g., in hemorrhagic pituitary adenoma, pituitary apoplexy, Sheehan syndrome, or thrombosed aneurysm), high protein concentration (e.g., Rathke cleft cyst, craniopharyngioma, or mucocele), fat accumulation (e.g., lipoma, dermoid cyst, lipomatous meningioma), calcification (e.g., craniopharyngioma, chondroma, chordoma) and presense of paramagnetic substances within lesions (e.g., manganese, melanin).
T1 signal hyperintensity in post-treatment setting in this region may result from materials used for surgical packing (e.g., gelatin sponge, fat), compression of the cavernous sinus due to overpacking of the operative bed, and hormone hypersecretion by remnants of normal tissue in the anterior lobe of the pituitary gland.

**Imaging Findings and Procedure Details:** We highlight the usefulness of the T1 hyperintense signal in the evaluation of sellar and para-sellar lesions on MRI by showcasing a myriad of interesting cases which presented to our department. The following normal variants and pathologic entities will be discussed in an image-based approach.
1. Hemorrhagic pituitary adenoma
2. Pituitary apoplexy
3. Sheehan syndrome
4. Thrombosed aneurysm
5. Craniopharyngioma
6. Rathke cleft cyst
7. Sphenoid sinus mucocele
8. Lipoma
9. Dermoid cyst
10. Cholesterol granuloma
11. Melanoma
12. Teratoma
13. Metastasis
14. Post operative change

**Conclusion:** Radiologists should be aware of the importance of T1 hyperintense signal in sellar and para-sellar region MRI to formulate a differential diagnosis and provide an accurate diagnosis.

### Temporomandibular Joint with MRI: Review of Anatomy and Imaging Features

*Author: C. Görürgöz; Bolu/TR*

**Learning Objectives:**
1. Anatomy of the temporomandibular joint: mandibular condyle, articular disc, glenoid fossa, and articular eminence.
2. Common temporomandibular joint disorders and their respective imaging features

**Background:** The anatomy of the temporomandibular joint (TMJ) is an area that is unfamiliar to many radiologists and can be overlooked or misinterpreted in a routine search pattern.
There is a wide range of pathologies that can be present in this relatively small anatomical area, some of which can be diagnosed and evaluated clinically. However, in some situations, imaging may be necessary, allowing the radiologist to provide important information to the referring physician and help guide further treatment and planning.

**Imaging Findings and Procedure Details:**

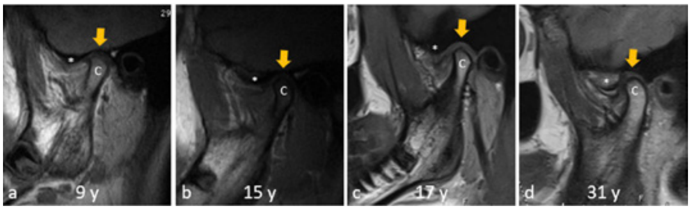

Figure 1: Age-dependent bony configuration of the temporomandibular joint a. Superior articular contour is round with a straight condylar neck. With age and growth (c-d), the condylar neck gains an anterior slope and the head appears more angular with less rounding of the anterior-superior articular surface.

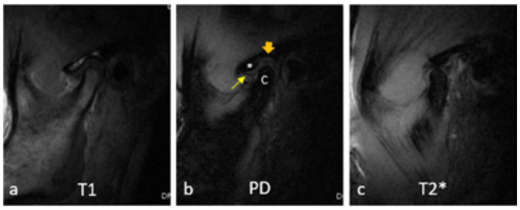

Figure 2: Morphologic features of the normal disc in a 25-year-old female. a-b. Sagittal oblique MR images (closed-mouth position), the anterior and posterior bands are thick and the intermediate zone is thin, creating a biconcave disc shape. c. The disk (arrow) has maintained its normal position during condylar movement

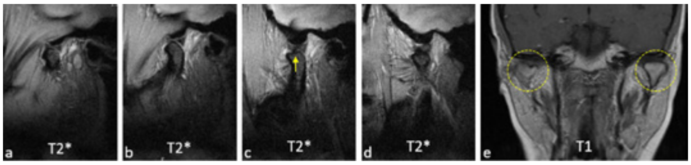

Figure 3: Developmental anomaly. a-d. Sagittal oblique gradient-echo T2*-weighted MR images obtained in the open-mouth position e. Coronal spin-echo T1-weighted MR image (closed-mouth position) shows that the condylar heads (circles). Demonstrating the Y-appearance of the right condyle.
This poster will primarily focus on the MR imaging characteristics of the anatomy and various pathologies. These include disorders of the condyle-disc complex, structural incompatibility of the articular surfaces, and inflammatory disorders of the TMJ.
The potential role of MRI techniques for lesion characterization will be emphasized with a focus on clinical relevance.

**Conclusion:** This poster provides a comprehensive review of the radiologic aspects of the anatomy and relevant pathologies of the TMJ. It is important for the radiologist to detect early signs of dysfunction on MR imaging to prevent this condition from progressing to its final stage, an advanced and irreversible phase characterized by osteoarthritic changes such as condylar flattening or osteophytes.

### The Clinical Presentation and Imaging Findings of Extra Nodal Head and Neck Lymphomas

*Authors: P. Shah, F. Gill, K. Rajaguru; Stevenage/GB*

**Learning Objectives:** To review the different clinical presentations of primary extra nodal head and neck lymphoma with reference to five cases highlighting the image findings and secondary clinical features associated.
**Background:** Lymphomas constitute a heterogeneous group of malignancies subdivided into Hodkins Lymphoma (HL) and Non Hodkins Lymphoma (NHL). Further division of NHL is based on cell lineage including B cell, T cell and natural killer cell lymphomas. Primary lymphoma accounts for 5% of all head and neck malignancies [1].
They most commonly arise as the result of the proliferation of lymphoid tissue with tumour transformation predominantly within nodal sites. Extra nodal lymphomas arise in Waldeyers ring or extra lymphatic tissue within the head and neck [2]. They often present with clinical and diagnostic uncertainty mimicking an epithelial neoplastic process or infection.
**Imaging Findings and Procedure Details:** We discuss the presentation and imaging of five cases of extra nodal primary head and neck lymphomas including:
- A growing subcutaneous lump on the forehead representing a subgaleal high grad B cell lymphoma. The patient additionally had features of angioedema
- A large right neck lump causing difficulty swallowing diagnosed as a high grade thyroid B cell lymphoma.
- A progressive history of headaches and double vision confirmed as an orbital B cell lymphoma of the left superior rectus extraocular muscle.
- A nasal lump diagnosed as a low grade NHL
- A left neck lump with associated breathing difficulty confirmed as a hypopharyngeal B cell NHL

The use of ultrasound, CT, PET-CT and MR imaging was utilised for primary workup and staging, with biopsy for histopathological confirmation. CT imaging characteristics are of that of a soft tissue mass often with contrast enhancement. MRI imaging features are of low signal on T1 weighted imaging, iso to high signal on T2 weighted imaging, variable contrast enhancement and diffusion restriction.
**Conclusion:** Primary extra nodal head and neck lymphomas often present as masses in atypical locations or mimicking tumours/infection in more classical locations. They should always be considered within the differential given the unusual clinical appearance and similar imaging appearance to epithelial tumours, especially in the case of high grade lymphomas.

References:
[1] V Diehl 1, M Sextro, J Franklin, M L Hansmann, N Harris, E Jaffe, S Poppema, M Harris, K Franssila, J van Krieken, T Marafioti, I Anagnostopoulos, H Stein, (1999), Clinical presentation, course, and prognostic factors in lymphocyte-predominant Hodgkin's disease and lymphocyte-rich classical Hodgkin's disease: report from the European Task Force on Lymphoma Project on Lymphocyte-Predominant Hodgkin's Disease, Journal Of Clinical Oncology
[2] Urquhart A and Berg R, (2001), Hodgkin's and non-Hodgkin's lymphoma of the head and neck, Laryngoscope

### The Sphneoid Sinus: The last but not least

*Authors: T. Hancıoglu[1], Y. Pekçevik[2], B. Ergin[2], I.B. Arslan[2]; [1]Manisa/TR, [2]Izmir/TR*

**Learning Objectives:**
- To review the key points of anatomy and embryology of the sphenoid sinus.
- To show clinically relevant variations of the sphenoid sinus.
- To demonstrate isolated sphenoid sinus pathologies from a broad perspective and evaluate them on case-by-case basis.

**Background:** Isolated sphenoid sinus pathologies (ISSP) are relatively uncommon. Although location of the sphenoid sinus and its close relationships with the critical anatomic structures, in the majority of ISSPs symptoms do not become evident at the onset of the diseases. In addition, the symptoms are usually nonspecific. These lead to delayed treatment and life-threatening clinical consequences. [1]
We aim to review key points of anatomy and embryology of the sphenoid sinus with clinically relevant variations, to demonstrate ISSPs case-by-case basis to establish a diagnostic approach.
**Imaging Findings and Procedure Details:**
Embryology, anatomy, and clinically relevant variations
The sphenoid sinus is one of the four paired paranasal sinuses (PNSs) which is not pneumatized at birth. The degree of pneumatization shows different patterns depending on age. According to the evidence obtained, pneumatization generally begins between 4 months and 6 years of age and ends around 12 to 14 years of age.[3]

1-Variation in septations
2-Variation in aeration

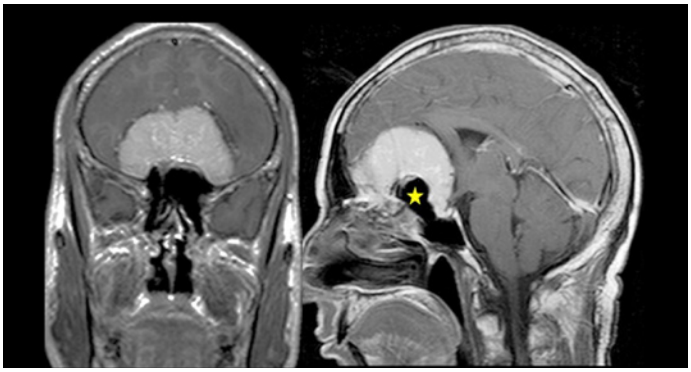

A young man, who was complaint about serious headache, applied neurology department. Contrast-enhanced coronal and sagittal T1-weighted images show anterior skull base meningioma. The mass is located on the planum sphenoidale. Also, the focal enlargement (asterisk) and upward displacement of the roof of sphenoid sinus can be seen.

3-Sphenoethmoidal cells
4-Protrusion of adjacent anatomic structures (vidian nerve, ICA, optic nerve)

Pathologies of the sphenoid sinus:[2]
1-Inflammatory disease
-Bacterial sinusitis
-Fungal sinusitis (invasive and non-invasive forms)
-Mucocele/ Pyocele

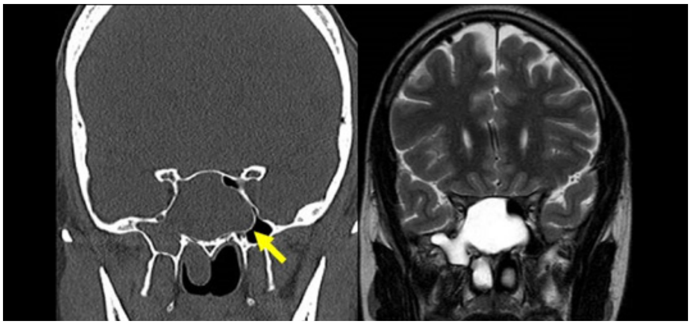

CT of the paranasal sinus shows a low-density opacification in the sphenoid sinus and expansion of the sinus. Always check the margins of the sinus. Note the intact bone with remodeling. T2-weighted coronal MRI Image demonstrates a mass with lobulated contour and high intensity. The signal intensity of the mass is related to content.

2-Tumors
-Primary tumors
Benign and malign tumors
-Secondary involvement
Invasive pituitary adenoma, nasopharyngeal carcinoma, metastatic tumors

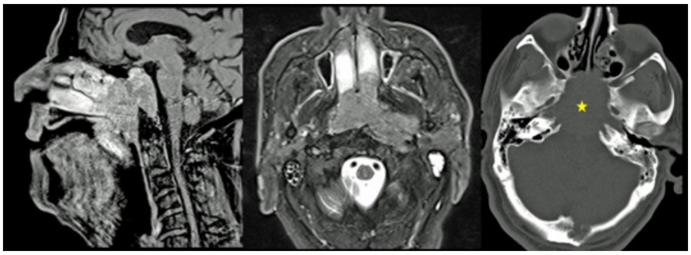

MRI scans of a 65-year-old man with nasopharyngeal carcinoma shows an ill-defined mass arising from the nasopharynx. The mass extends upwards through the nasal cavity and sphenoid sinus. The mass leads to nasal obstruction. It causes extensive bone destruction, notably of the central skull base.

-Systemic diseases
Granulomatosis with poliangiitis

Miscellaneous:
1-Lateral sphenoid cephaloceles
2-Sphenoid pseudo lesions (arrested pneumatization)

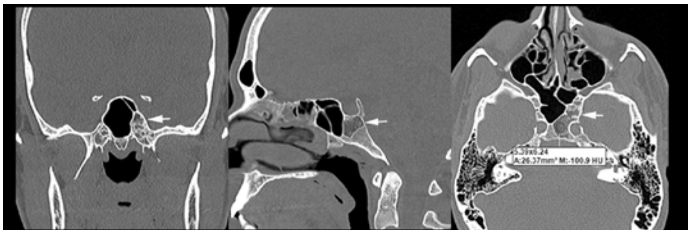

CT features of intraosseous lipomatous lesion. Coronal, sagittal and axial reformatted images of paranasal sinuses demonstrate well-defined margin, slightly expansile lesion with sclerotic border. The content of the lesion is heterogenous. The mean density measured -100 HU, that is, it shows the density of the fat.

**Conclusion:** ISSPs occur rarely and are usually represented by non-specific symptoms. Most of the times, more obvious and diagnostically life-threatening symptoms appear in the advanced stage of the disease. Thus, the sphenoid sinus lesions can be easily overlooked. To make an early diagnosis, radiologists must have sufficient knowledge of the anatomy and pathology of the sphenoid sinus. Awareness and suspicion of ISSPs is the main step in the diagnostic approach especially with clinical findings.

References:
[1] Sethi DS., (1999), Isolated sphenoid lesions: diagnosis and management, Otolaryngology-Head and Neck Surgery, 7306, 5
[2] Fujioka M, Young LW., (1978), The sphenoidal sinuses: radiographic patterns of normal development and abnormal findings in infants and children. , 133, 1
[3] Friedman A, Batra PS, Fakhri S et al, (2005), Isolated sphenoid sinus disease: Etiology and management, Otolaryngology-Head and Neck Surgery, 544-50, 4

### Triton tumours in the head and neck

*Authors:* *J. Flexen, S. Otero; London/GB*

**Learning Objectives:**
- Awareness of the pathophysiology of malignant triton tumours in the head and neck region
- Become familiar with the spectrum of imaging features
- Appreciate the difficulty in diagnosis

**Background:** Malignant triton tumours are a form of soft tissue sarcoma, derived from malignant peripheral nerve sheath tumours that undergo rhabdomyoblastic differentiation. This is a rare tumour, with only around 200 cases reported in the literature and of those cases roughly 1/3 occur in the head and neck (1,2). There is an association with neurofibromatosis 1 although they can also occur sporadically. The mainstay of treatment is surgical resection with wide margins +/- adjuvant therapies (3).

**Imaging Findings and Procedure Details:** We will present a range of cases from our regional head and neck sarcoma centre. We will exhibit ultrasound, computed tomography and magnetic resonance imaging examples of triton tumours in the head and neck. All cases have histopathological confirmation.

**Conclusion:** Malignant triton tumours in the head and neck are rare and can mimic other tumours radiologically. Diagnosis can therefore be difficult, and a multidisciplinary approach is required.

### Usual and unusual lesions presenting with cranial nerve palsies at the region of orbital apex – An image based review

*Authors: P. Gupta, M.S. Swarup, S. Jain; New Delhi/IN*

**Learning Objectives:**
1. To provide an image-based review of various common and uncommon lesions causing cranial nerve palsies at the orbital apex
2. To highlight characteristic imaging findings and discuss the differentials

**Background:** Lesions at orbital apex region are readily identified on cross -sectional images. Patients can present with signs and symptoms deriving from the involvement of structures within the orbital apex, the superior orbital fissure or the cavernous sinus including ophthalmoplegia, ptosis, blurring of vision, visual deficits. The differential diagnosis of the cranial nerve palsies at the orbital apex can be identified on the basis of morphologic characteristics on cross sectional images (attenuation at computed tomography, signal intensity at magnetic resonance imaging, enhancement pattern, shape and margins, extent, mass effect, and adjacent bone reaction).

**Imaging Findings and Procedure Details:** 3T MRI brain with orbit was done in 15 patients presenting with deficits due to involvement of IInd to VIth cranial nerves. Seven patients had inflammatory cause, three each of vascular and infective cause while two had neoplastic etiology.
1. Inflammatory- Tolosa Hunt syndrome, inflammatory pseudotumor, IgG related disease
2. Tumours- Meningioma, metastasis
3. Vascular- carotid-cavernous fistula, cavernous ICA aneurysm cavernous sinus thrombosis
4. Infections- Tuberculosis, staphylococcal, oculo-rhino-cerebral mucormycosis

**Conclusion:** The knowledge of patient's age, signs and symptoms, exact location of lesion, extension and characteristic imaging findings can help the radiologists in narrowing the differential diagnoses.
